# Cellular senescence limits translational readthrough

**DOI:** 10.1242/bio.058688

**Published:** 2021-12-02

**Authors:** Neylen del Toro, Frédéric Lessard, Jacob Bouchard, Nasrin Mobasheri, Jordan Guillon, Sebastian Igelmann, Sarah Tardif, Tony Buffard, Véronique Bourdeau, Léa Brakier-Gingras, Gerardo Ferbeyre

**Affiliations:** 1 Département de Biochimie et Médecine Moléculaire, Université de Montréal C.P. 6128, Succ. Centre-Ville, Montréal, Québec, H3C 3J7, Canada; 2 CRCHUM-Université de Montréal, 900 Saint-Denis, bureau R10.432, Montréal, Québec, H2X 0A9, Canada

**Keywords:** Senescence, Translational readthrough, Translation termination, Retinoblastoma (RB) tumor suppressor

## Abstract

The origin and evolution of cancer cells is considered to be mainly fueled by DNA mutations. Although translation errors could also expand the cellular proteome, their role in cancer biology remains poorly understood. Tumor suppressors called caretakers block cancer initiation and progression by preventing DNA mutations and/or stimulating DNA repair. If translational errors contribute to tumorigenesis, then caretaker genes should prevent such errors in normal cells in response to oncogenic stimuli. Here, we show that the process of cellular senescence induced by oncogenes, tumor suppressors or chemotherapeutic drugs is associated with a reduction in translational readthrough (TR) measured using reporters containing termination codons withing the context of both normal translation termination or programmed TR. Senescence reduced both basal TR and TR stimulated by aminoglycosides. Mechanistically, the reduction of TR during senescence is controlled by the RB tumor suppressor pathway. Cells that escape from cellular senescence either induced by oncogenes or chemotherapy have an increased TR. Also, breast cancer cells that escape from therapy-induced senescence express high levels of AGO1x, a TR isoform of AGO1 linked to breast cancer progression. We propose that senescence and the RB pathway reduce TR limiting proteome diversity and the expression of TR proteins required for cancer cell proliferation.

## Abstract (precis)

**Summary:** We report that senescence and the RB pathway reduce translational readthrough (TR) limiting proteome diversity and the expression of TR proteins such as Ago1X required for cancer cell proliferation.

## INTRODUCTION

Cellular senescence is a tumor suppressor mechanism that prevents proliferation in cells bearing oncogenic stimuli (Collado and Serrano, 2010). Senescent cells can efficiently halt tumor progression by remaining out of the cell cycle permanently as benign lesions (Collado and Serrano, 2010; Vernier and Ferbeyre, 2014). Ideally, they also activate their elimination through immune mediated clearance (Kang et al., 2011; Xue et al., 2007). However, if not cleared, some senescent cells can occasionally escape from their dormancy and progress into malignant tumors (Milanovic et al., 2018; Romanov et al., 2001). There is a great heterogeneity in the senescence response depending on the inducer, tissue type and genetic background (Hernandez-Segura et al., 2017). Nevertheless, most senescent cells activate the p53 and retinoblastoma (RB) tumor suppressor pathways that block cell cycle progression (Collado and Serrano, 2010; Vernier et al., 2011; Vernier and Ferbeyre, 2014). In epithelial cells, reactive oxygen species, defective DNA repair and DNA damage were linked to senescence bypass and malignant progression (Gosselin et al., 2009; Nassour et al., 2016). It is commonly accepted that DNA damage and mutations fuel tumor initiation and progression (Harfe and Jinks-Robertson, 2000; Kandoth et al., 2013) by generating both oncogenic drivers and genetic diversity (Bielas et al., 2006). However, little is known about the contribution of translational errors in tumorigenesis.

In normal cells, translational errors in the form of aminoacid misincorporation are estimated to occur at a reduced frequency and unlikely to significantly affect the proteome (Drummond and Wilke, 2009). In microorganisms, mistranslation contributes to adaptive evolution by purging deleterious mutations and inducing stress adaptation (Bratulic et al., 2017; Ribas de Pouplana et al., 2014; True et al., 2004). Moreover, translational recoding is often used by viruses to increase the coding potential of their small genomes (Bidou et al., 2010). For example, retroviruses use frameshifting to control the synthesis of viral replication proteins (Charbonneau et al., 2012). Overall, translational readthrough (TR), originally discovered in viruses and now extended to metazoans, can add a new C-terminal signal to proteins that may change their function and localization (Jungreis et al., 2011; Schueren et al., 2014; Schueren and Thoms, 2016; Singh et al., 2019). The extensions usually contain potential signals to target proteins to the nucleus, peroxisomes or the membrane (Schueren et al., 2014; Schueren and Thoms, 2016; Singh et al., 2019). Taken together, the available evidence suggests that tumor cells could use such recoding mechanisms to generate new protein variants and evolve. Therefore, tumor suppressor pathways could counteract TR as part of their mechanism of action.

TR results from the recognition of stop codons (UAA, UAG and UGA) by near cognate tRNAs instead of the release factors (RFs) that can also recognize the termination codons to end translation (Tate et al., 2018). The efficiency of translation termination depends on the identity of the stop codon (Firth et al., 2011; Grentzmann et al., 1998; Loughran et al., 2014) and is modulated by metabolic stress in bacteria (Zhang et al., 2020) or oxidative stress in yeast (Gerashchenko et al., 2012). However, little is known about the processes controlling TR in mammalian cells.

Here, we report that TR is significantly reduced during oncogene-induced senescence (OIS) or therapy-induced senescence (TIS), which are anticancer responses controlled by the tumor suppressors RB, p53 and PML. Using defined genetic tools, we also show that senescence-associated readthrough suppression is mostly mediated by the RB tumor suppressor pathway. In addition, we show that cells that escape from senescence increase the expression of AGO1x, an endogenous TR isoform of AGO1 that suppress the interferon pathway stimulating cell proliferation in breast cancer cells (Ghosh et al., 2020). Hence, like DNA mutations, TR can both increase the expression of specific cancer drivers and generate proteome diversity fueling cancer cell evolution.

## MATERIALS AND METHODS

### Cell culture and materials

IMR-90 human diploid fibroblasts were obtained from Coriell Institute for Medical Research (New Jersey, NY). Prostate Cancer cells (PC-3) and MDA.MB.231 breast cancer cells were obtained from ATCC. A certificate of authentication was obtained from Coriell for IMR-90 cells and from ATCC for PC-3 and MDA.MB.231 cells. All cells were mycoplasma free after testing that was performed once a month for all cell lines by immunofluorescence. IMR-90 containing human telomerase reverse transcriptase (hTERT) were generated in our laboratory by transducing cells with the FG12-hTERT lentiviral vector (Voghel et al., 2010). Phoenix ampho packaging cells used for retroviral infections were given by S.W. Lowe (Memorial Sloan Kettering Cancer Center, New York, NY). IMR-90 and MDA.MB.231 were cultured in Dulbecco's modified Eagle medium (DMEM; Wisent) supplemented with 10% fetal bovine serum (FBS; Wisent), 1% penicillin G/streptomycin sulfate (Wisent) and 2 mmol/l L-glutamine (Wisent). PC-3 were cultured in Roswell Park Memorial Institute Medium (RPMI 1640; Wisent) supplemented with 10% fetal bovine serum (FBS; Wisent), 1% penicillin G/streptomycin sulfate (Wisent) and 2 mmol/l L-glutamine (Wisent).

The translation elongation inhibitor cycloheximide and the aminoglycoside gentamicin sulfate were purchased from Sigma-Aldrich (Oakville, ON). The CDK4/6 inhibitor palbociclib (PD**-**0332991) was purchased from Chemietek. Camptothecin and 4-hydroxy-tamoxifen were purchased from Sigma-Aldrich (Oakville, ON). A phthalamide derivative named CDX5-1 was donated by M. Roberge (University of British Columbia, Vancouver, BC, Canada).

### Plasmids and cloning

Retroviral vectors pBabe/pBabe-ER, pBabe-H-RasV12, pWZL/pWZL-H-RasV12 were described in (Ferbeyre et al., 2000), pBabe-PML-IV-ER in (Vernier et al., 2011), pLXSN, pLXSN-E6, pLXSN-E7, pLXSN-E6/E7 and pLXSN-E7 **Δ** 21-24 in (Mallette et al., 2004), pBABE-RPL22(WT)-Myc in (Del Toro et al., 2019), pBABE-RPS14(WT)-Myc in (Lessard et al., 2018). pBABE-CDK4(WT) was a gift from Scott W. Lowe (Memorial Sloan-Kettering Cancer Center, New-York, NY, USA). The genes coding for Renilla luciferase (Rluc) and Firefly luciferase (Fluc) were linked by an intercistronic region in order to obtain a 96 kDa bifunctional protein (Gendron et al., 2005). These reporters were PCR-amplified and subcloned in NotI/NsiI restriction sites to obtain pMSCV-Rluc-Fluc variants. A UGA stop codon, created by site-directed mutagenesis using PfuUltra II fusion HS DNA polymerase (Agilent, Canada), was inserted in the intercistronic sequence. Primers for PCR are provided in Table S1. The readthrough region from Moloney Murine Leukemia Virus (MMuLV) as well as sequences flanking the stop codon from the readthrough sequence of Aquaporin 4 (AQP4, NM_001650.4) (Loughran et al., 2014) were chemically synthesized (Biocorp, Canada) and subcloned in XhoI/ApaI restriction sites between Rluc and Fluc genes. The readthrough region sequences are provided in Table S2. The non-readthrough control was performed by mutating the stop codon TGA to CGA.

### Identification of readthrough candidates in mammalian cells using bioinformatic analysis

In order to look for endogenous readthrough candidates, we looked in silico for peptides encoded after termination codons detected in proteomics experiments. We first built a database of peptide sequences located between the first arginine/lysine after the canonical stop codon and the next stop codon, always in the same reading frame using sequences deposited in RefSeq hg38.2. We discarded sequences shorter than five aminoacids. Candidate readthrough peptides were then matched to the data set of peptides identified by trypsin digestion of human proteins and mass spectrometry published in (Rosenberger et al., 2014). We found 278 candidates for endogenous readthrough above the false discovery rate (FDR) threshold (Table S3). Following a readthrough propensity predictor algorithm (Schueren et al., 2014), four candidates showing the highest readthrough propensity values were chosen. This algorithm assigns regression coefficients to the stop codon and all possible nucleotides in the stop codon context based on experimental data. The stop codon context comprises the nucleotide sequences from −6 to +9 positions surrounding the stop codon. The new readthrough candidates are: Vasodilator-stimulated phosphoprotein (VASP, NM_003370.3), Aspartate beta hydroxylase (ASPH, NM_004318.3), Hepsin (HPN, NM_182983.2) and Fibrillarin (FBL, NM_001436.3). Oligonucleotides containing 30 up-stream and 30 down-stream nucleotides flanking the stop codon, as well as the stop codon of those readthrough candidates were chemically synthesized (Biocorp, Canada) and subcloned in XhoI/ApaI restriction sites between Rluc and Fluc genes of our pMSCV-Rluc-Fluc reporter construct. The sequences of all new readthrough candidates’ regions are provided in Table S2.

### Polysome fractionation

The protocol was performed as previously described (Gandin et al., 2014). Briefly, 60% (w/v) sucrose stock solution was used to make sucrose gradients (5 to 50%) in a buffer containing 200 mM Tris-HCl (pH 7.6), 1 M KCl, 50 mM MgCl_2_, 100 µg/ml cycloheximide, 1X cOmplete EDTA-free protease inhibitor cocktail (Roche) and 200 units/ml of RNase inhibitor (abm-Applied Biological Materials, BC). After 12 days post infection, proliferating and senescent fibroblasts at 80–90% confluence were treated with cycloheximide at a final concentration of 100 µg/ml for 5 min at 37°C. Cells were washed with ice-cold 1X PBS, 100 µg/ml cycloheximide, scratched and lysed in a hypotonic buffer (5 mM Tris-HCl pH 7.5, 2.5 mM MgCl_2_, 1.5 mM KCl, 1X cOmplete EDTA-free protease inhibitor cocktail, 100 µg/ml cycloheximide, 1 mM DTT, 100 units of RNAse inhibitor, followed by addition of 25 μl of 10% Triton X-100 (final concentration of 0.5%) and 25 μl of 10% sodium deoxycholate (final concentration of 0.5%). Cells were vortexed and then centrifuged at low speed (140×***g***) for 15 min at 4°C. Sample supernatants were adjusted so that they contain the same OD (10–20 OD at 260 nm). The lysate was loaded onto the ultracentrifuge tubes containing sucrose gradients, then centrifuged at 22,223×***g*** (36,000 rpm) for 2 h at 4°C using SW41Ti rotor Beckman Coulter (Optima L80 XP ultracentrifuge). After ultracentrifugation, the samples were placed in the UV detector (Brandel #IV-22-117140) and fraction collector (Retriever 500, Teledyne Isco). Each fraction was collected and monitored from the top to the bottom of the ultracentrifuge tube.

### Dual-luciferase assays

IMR-90 transduced cells were washed with 1X PBS, scratched and lysed in 1X Passive lysis buffer supplied in the Dual Luciferase Stop & Glo^®^ Reporter Assay System (Promega). A 20 μl cell lysate sample was used for luminescence measurements with Lumin Triathler-Hidex. After adding 50 μl of the Fluc reagent (Substrate+Buffer) to the samples, luminescence was measured for 10 s. Addition of 50 μl of the Rluc reagent-Fluc quenching (Stop & Glo^®^) preceded another 10 s measurement. The efficiencies of readthrough (% Readthrough) were determined by averaging the relative Fluc activities (Fluc/Rluc ratio) from luciferase reporters UGA or AQP4, divided by the relative luciferase activities of their non-readthrough controls.

We also compared the readthrough fold-changes, relative Fluc activities (Fluc/Rluc) of each condition were normalized to the relative Fluc activities from non-senescent cells:

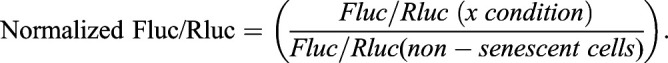


To measure readthrough after therapy-induced senescence, PC3 and MDA.MB.231 cells were retrovirally transduced with luciferase reporter AQP4 or UGA. Transduced cells were treated 24 h using 100 nM of camptothecin or vehicle (DMSO), to induce senescence. Following the treatment, cells were washed with 1X PBS and fresh medium was added. Luciferase activities were measured at day 7 post treatment. All assays were performed in technical triplicates. Normalized relative Fluc fold changes and Fluc/Rluc ratios are provided in Table S4 as well as all biological replicates.

### Immunoblotting and Immunofluorescence

To prepare total cell extracts, cells were washed with 1X PBS containing 1X protease- and phosphatase-inhibitor cocktails (Roche), scratched and collected by centrifugation at low speed (140×***g***) for 5 min, lysed in 200 μl of SDS sample buffer (62.5 mM Tris-HCl, pH 6.8, 10% glycerol, 2% SDS and 5% 2-mercaptoethanol), and boiled for 5 min. Fifteen μg of total cell proteins were separated on SDS-PAGE and transferred to nitrocellulose membranes (Millipore). The primary antibodies used were anti-Renilla Luciferase rabbit polyclonal (1:3000, Code No. PM047, MBL), anti-H-Ras mouse monoclonal (1:250, clone F235, sc-29, Santa Cruz Biotechnology, CA, USA), anti-phospho-H3^S10^ rabbit polyclonal (1:1000, #06–570, Millipore, Billerica, MA, USA), anti-phospho-RB^S795^ rabbit polyclonal (1:500, #9301, lot: 13, Cell Signaling), anti-RB mouse monoclonal (1:1000, clone 4H1, #9309, Cell Signaling Technology), anti-MCM6 rabbit polyclonal (1:1000, A300-194A, Bethyl Laboratories), anti-p53 mouse monoclonal (1:1000, clone DO-1, sc-126, Santa Cruz Biotechnology), anti-p21 (1:500, 556431, BD Pharmingen), anti-RNR (1:500, sc-398294, Santa Cruz Biotechnology), anti-phospho-γH2A.XS139 mouse monoclonal (1:500, JBW-301, lot: 2552645, Millipore, Billerica, MA), anti-c-Myc rabbit polyclonal (1:1000, clone A-14, sc-789, Santa Cruz Biotechnology) and anti-α-tubulin mouse monoclonal (B-5-1-2, 1:20000, Sigma-Aldrich). Signals were revealed after incubation with goat anti-mouse IgG (1:3000, #170-6516, Bio-Rad, Mississauga, ON, Canada) or goat anti-rabbit IgG (1:3000, #170-6515, Bio-Rad) secondary antibodies, and by using enhanced chemiluminescence (ECL, Amersham) or Lumi-LightPLUS (Roche).

Immunofluorescence were done as described (Igelmann et al., 2021) with the following modifications for the AGO1x staining. For blocking 10% goat serum (#16210072, Life Technologies), and 1% BSA (BioShop Canada) in PBS for 30 min. Primary antibodies were AGO1x (1:50, #RBP 1510, Lucerna-Chem.) and KI67 (1:200, Cat# RM9106, ThermoFisher Scientific). Cells were analyzed using upright microscope Zen Imager with 20X air objective. Images were processed using Image J and quantification was done using region of interest (ROI) mean intensity staining. To determine ROI, DAPI staining was used.

### Cell proliferation assay and senescence-associated β-galactosidase assay

Relative density of cells was assessed from estimations of cell number according to crystal violet retention assay (Vernier et al., 2011). The senescence-associated β-galactosidase (SA-β-Gal) activity was measured at day 7, 12 or 35 post-infection as previously described (Vernier et al., 2011). All assays were performed in technical triplicates.

### RT-qPCR

Senescent and non-senescent IMR-90 s were collected at 12- or 35-days post-infection, with either pBabe-H-RasV12 or pBabe control vector in TRIzol reagent (Invitrogen) and total RNA was extracted following manufacturer's instructions. Two μg of total RNA were then used for the RT-qPCR reactions as described in (Igelmann et al., 2021). Primers for qPCR were designed using the Universal Probe Library Assay Design Center (https://www.roche-applied-science.com/sis/rtpcr/upl/index/jsp) and are provided in Table S5. Analysis for indicated genes were done using the ΔΔCT method. HMBS and TBP were used as reference genes (Vernier et al., 2011). All assays were performed in technical triplicates.

### Statistics and reproducibility

Statistical analysis (one-way ANOVA with post-hoc Tukey’s honestly significant difference (HSD) test) was done using the one-way ANOVA test calculator at: http://astatsa.com/OneWay_Anova_with_TukeyHSD/. Two-tailed Student's *t*-tests were performed using GraphPad Prism version 6.0c software. A *P*<0.05 was considered statistically significant. Each experiment was repeated at least three times, except for those in Fig 2C,D, Fig 3B,C (UGA); Fig S1C­­–E and Fig S2D, which were done twice. SA-β-Gal assays were quantified from many fields within one experiment to represent the entire petri dish and confirmed as described in Figs 5F, 6A, 7A and Fig. S1A.

## RESULTS

### Translation termination is improved in oncogene-induced senescence

**Fig. 1.**
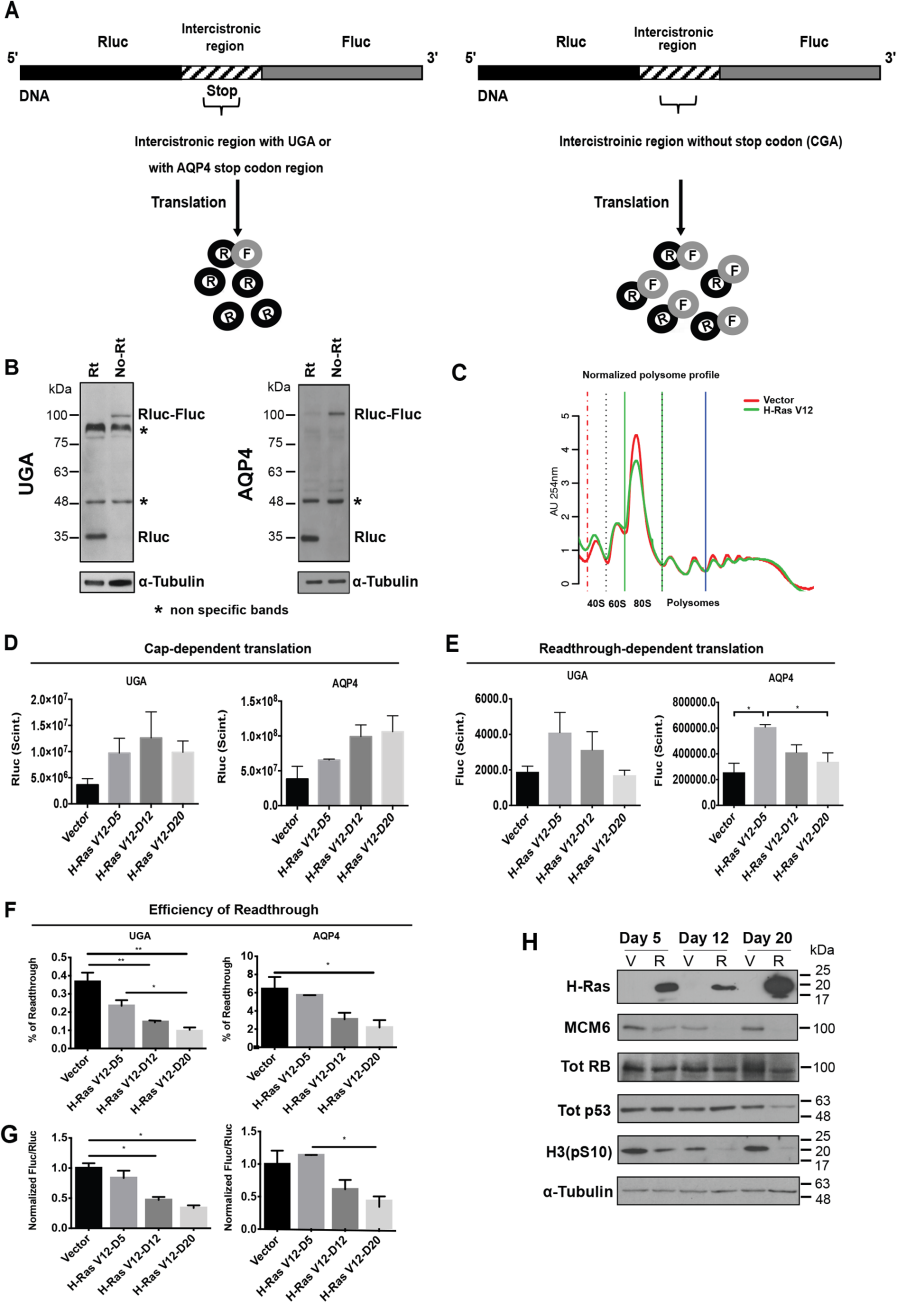
**Readthrough is reduced in OIS.** (A) Rluc (Renilla luciferase) in black is linked to Fluc (Firefly luciferase) in gray, by an intercistronic region in stripes. Rluc is the internal control of the gene, while Fluc is the readthrough sensor. A UGA codon within an artificial context or the AQP4 stop codon region is inserted in the intercistronic region (left). The expression of Fluc indicates the efficiency of readthrough. The intercistronic region from the non-readthrough control lacks the stop codon (TGA mutated by CGA) (right). (B) Immunoblots for Rluc in IMR-90 cells transduced with UGA and AQP4 reporters (Rt) or with the non-readthrough controls (No Rt), 12 days post infection. Alpha-Tubulin was used as a loading control. *, non-specific bands at 48 and 90 kDa; *n*=3. (C) Polysome profiles were performed 12 days post infection with an empty vector (Vector) or H-Ras V12 oncogene (H-RasV12) showing that global translation is similar in senescent and non-senescent IMR-90 cells, *n*=3. (D) Rluc plots indicate cap-dependent translation in IMR-90 cells with an empty vector (Vector) or with the oncogene H-Ras V12 (H-RasV12) at day 5 (D5, still proliferating), 12 (D12, senescent cells) or 20 (D20, senescent cells). Error bars indicate s.d of three independent experiments, *n*=3. (E) Fluc plots indicate the decrease of readthrough-dependent-translation of cells as in D. Error bars indicate s.d. of three independent experiments, *n*=3. (F) The percent of readthrough was calculated in cells as in D by dividing the Fluc/Rluc ratio from UGA or AQP4 luciferase reporters by the Fluc/Rluc ratio from non-readthrough controls multiplied by 100, *n*=3. (G) Fluc/Rluc ratios with data as in F but normalized relative to empty vector-infected cells *n*=3. (H) Immunoblots of H-Ras, total RB (Tot RB), total p53 (Tot p53), MCM6, phosphorylated Histone H3 on serine 10 [H3(pS10)] and tubulin at days 5, 12 and 20 post infection with an empty vector (V) or H-RasV12 oncogene (R), *n*=3, representative blot is shown. For panels E, F and G, significance was tested with one-way ANOVA with post-hoc Tukey HSD tests. Error bars indicate SD of three independent experiments. Tukey HSD *P*-values indicate that *=*P*<0.05 and **P<0.01.

To investigate the effect of cellular senescence on TR efficiency, we first used a model of oncogene-induced senescence (OIS) in human primary cells. Primary fibroblasts were retrovirally transduced with H-RasV12 oncogene (senescence inducer) or the control empty vector. They were also transduced with luciferase reporter plasmids containing either: (1) a TGA within an artificial intercistronic region (UGA readthrough); (2) the stop codon context from AQP4, a well-known mammalian readthrough candidate (Loughran et al., 2014); or (3) the non-readthrough controls (TGA mutated to CGA) between Rluc and Fluc fusion protein gene (Fig. 1A). Immunoblotting shows a band at 35 kDa corresponding to Renilla luciferase, as well as a weak band at 100 kDa indicating the fusion protein Renilla-Firefly luciferase produced after a ribosomal readthrough in cells transduced with the AQP4 readthrough reporter (Fig. 1B, right). For UGA reporter, the fusion protein is not detectable. On the other hand, in cells transduced with the non-readthrough control only the fusion protein can be detected (Fig. 1B, left). At early stages of H-RasV12 oncogene expression, cells present a cancer-like behavior characterized by a hyperproliferation phase. However, around 6 days after introduction of oncogenic *ras*, cells stop proliferation and enter a well-characterized and stable cell cycle arrest (Serrano et al., 1997). We confirmed this senescent cell cycle arrest at day 12 post infection, using an assay for the senescence-associated β-galactosidase activity that allowed visualizing characteristically large and flat cells that stained positive for this biomarker (Fig. S1A). RT-qPCRs were carried out to evaluate the decrease of Ki67 expression, indicating the proliferation arrest, and the activation of p53 targets (CDKN1A/p21, GADD45A) and RB pathway (CDKN2A/p16INK4a, MCM6) in senescent cells (Fig. S1B). Global translation was not affected in senescent cells since their polysome profiles were similar to control growing cells (Fig. 1C). This is consistent with previous reports and with the fact that senescent cells are actively secreting multiple pro-inflammatory mediators (Young et al., 2009). In addition, the non-readthrough (CGA) controls presented similar relative Fluc activities both in senescent and proliferating cells (Fig. S1C). Cap-dependent Rluc expression increased with time (Fig. 1D). Readthrough-dependent translation of the firefly luciferase reporter increased early after introduction of oncogenic *ras* in normal human fibroblasts when cells proliferate rapidly but it decreased when the cells underwent OIS as measured at days 12 or 20 after H-Ras-introduction (Fig. 1E). The relative efficiency of TR was calculated by dividing Fluc by Rluc values, then normalizing to the Fluc/Rluc ratio obtained in the same cells using the non-readthrough (CGA) control. The results clearly show that the senescent program reduced TR efficiency at UGA codons without any particular context and in the sequence flanking the AQP4 translation termination signal (Fig. 1F). Similar results can be seen after plotting the Fluc/Rluc ratio of senescent Ras-expressing cells relative to cells with empty vectors (Fig. 1G). In contrast, control cells did not change TR efficiency during the same time in culture (Fig. S1D). To discard the possibility that TR efficiency might be decreased due to a cell-cycle arrest independent from the senescent phenotype, proliferating cells were starved for a week to induce quiescence. Starved normal cells compared to cells supplied with fetal bovine serum (FBS) showed no differences in the efficiency of TR. Besides, starved and FBS-supplied senescent cells showed similar TR values (Fig. S1E). These observations lead us to conclude that the decrease of TR efficiency is senescence-specific. Immunoblots showed the gradual reduction of phosphorylated RB, the absence of the E2F target MCM6 and the reduction of the mitotic marker H3(pS10) in H-Ras cells, confirming the establishment of senescence. P53 accumulates in senescent cells but decreases after 20 days post-infection in H-Ras cells (Fig. 1H).

**Fig. 2.**
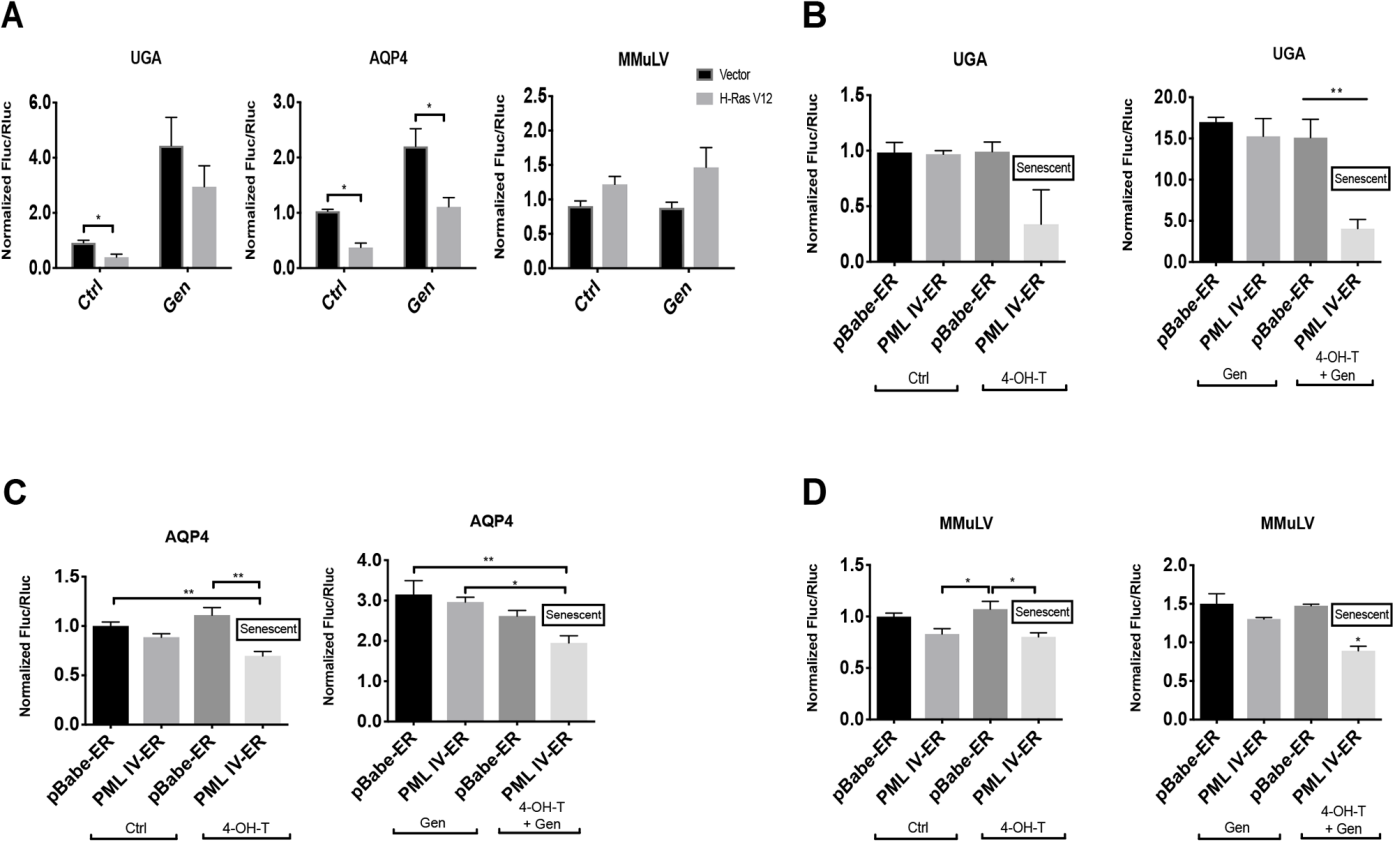
**Gentamicin-dependent translation errors are reduced in OIS and PML-induced senescence.** (A) IMR-90 cells were transduced with either an empty vector (Vector) or H-RasV12 oncogene (H-RasV12), to induce OIS, and with a luciferase reporter containing either: a UGA within an artificial context, a stop codon within the natural context from AQP4 or a MMuLV programmed readthrough region for gag-pol proteins inserted in the intercistronic region of the reporter. Cells were treated with vehicle (Ctrl) or 900 μg/ml of gentamicin sulfate (Gen) for 24 h before measuring luciferase activities at day 12 post infection. Unpaired Student's *t*-test were performed. Error bars indicate SD of biological triplicates. *=*P*<0.05 is significantly different, using two-tailed Student's *t*-test, *n*=3. B-D. IMR-90 cells were transduced with pBabe-ER empty vector (pBabe-ER) or pBabe-PML-IV-ER (PML IV-ER), and with luciferase reporters UGA (B), AQP4 (C) or MMuLV (D). Cells were treated with vehicle (Ctrl) or 100 nM 4-hydroxy-tamoxifen (4-OHT), inducing or not PML-IV nuclear translocation to induce senescence. Moreover, cells were treated with vehicle or 900 μg/ml of gentamicin sulfate (Gen) for 24 h before measuring luciferase activities at day 12 post PML-IV induction. Normalized Fluc/Rluc ratios indicate the efficiency of readthrough. Normalizations are presented as means relative to empty vector-infected cells from three independent experiments with technical triplicates for each experiment, except for (C,D), which are representative of two independent experiments with similar results. B-D: One-way ANOVA with post-hoc Tukey HSD were performed. Error bars indicate SD of biological triplicates (B) or technical triplicates (C, D). Tukey HSD *P*-values indicate that *=*P*<0.05, **=*P*<0.01 are significantly different.

The aminoglycoside gentamicin disrupts prokaryotic protein synthesis by binding to 16S ribosomal RNA, but also affects eukaryotic ribosome proofreading, inducing a conformational change in the ribosome-mRNA complex stimulating the efficiency of TR (Manuvakhova et al., 2000). We sought to investigate whether OIS can decrease TR stimulated by gentamicin. We added 900 μg/ml of gentamicin sulfate to OIS cells 24 h before luciferase assays. The non-readthrough controls were unaffected by gentamicin stimulation (Fig. S2A). Gentamicin increased TR efficiency and this effect was reduced in senescent cells both at UGA stop codons and the natural readthrough sequence of AQP4 (Fig. 2A; Fig. S2B,C). Of note, for the UGA reporter the TR efficiency was very small (0.1%) but increased more than 50-fold for the AQP4 reporter (6.9%). In both cases, the levels of TR were reduced by about a factor of 2 in senescent cells and we obtained similar results after gentamicin treatment (Fig. S2B,C). Strikingly, TR levels were the same in senescent and proliferating cells with the reporter containing the programmed readthrough region from Moloney Murine Leukemia Virus (MMuLV) (Fig. 2A). This region contains a UAG stop codon followed by a pseudo-knot structure (Houck-Loomis et al., 2011) and it allows up to 10% of ribosomes to suppress translation termination in order to synthesize the Gag-Pol polyprotein in cells infected with the virus (Yoshinaka et al., 1985). Also, the aminoglycoside gentamicin did not affect MMuLV TR efficiency.

We also treated cells 72 h before cells lysis with 600 μg/ml of gentamicin and 25 μM of CDX5-1, a novel small molecule that potentiates TR efficiency when combined with aminoglycosides although it has by itself no effect (Baradaran-Heravi et al., 2016). We observed that while single gentamicin treatment increased UGA TR efficiency up to 6.6-fold in control cells, the combination increased it up to 16.5-fold. Surprisingly, the TR efficiency was still limited in senescent cells after the combination, which improved the efficiency only 1.7-fold over gentamicin treatment alone (Fig. S2D). Together, these results indicate that senescence engages a significant barrier for TR.

To investigate more broadly whether senescence affects natural readthrough signals, we made luciferase reporters using the stop codon context of several mRNAs, which we predicted *in silico* as candidates for readthrough (see Materials and Methods). We tested both their basal and gentamicin-stimulated TR. We found that the TR of VASP and HPN were significantly reduced during OIS while the TR of FBL showed a tendency to be reduced (Fig. S2E). ASPH TR was undetectable neither in proliferating nor in senescent fibroblasts. Nevertheless, after gentamicin stimulation, we could observe the TR decrease in senescent cells (Fig. S2F).

### Translation termination is improved in PML-induced senescence

The tumor suppressor PML controls p53 and RB which are central for the establishment of OIS response (Vernier et al., 2011). Also, PML plays a role in antiviral responses (El Asmi et al., 2014; Regad et al., 2001) and, since many viruses use readthrough, we wanted to investigate whether PML modulates this process. Normal human fibroblasts IMR-90 cells were retrovirally transduced with a vector that allows expression of a conditionally inactive PML (pBabe-PML-IV-Estrogen Receptor) as well as with UGA, MMuLV and AQP4 luciferase reporters, in order to analyze TR efficiency in this model. Transduced cells were stimulated with 100 nM of the estrogen antagonist 4-hydroxy-tamoxifen (4-OHT), to induce PML-IV nuclear translocation and senescence (Acevedo et al., 2016). We found that induction of PML reduced basal or gentamicin-induced TR at UGA stop codons (Fig. 2B). Intriguingly, the effect of PML on gentamicin-induced readthrough was much more important than the effect of OIS, suggesting that PML could be one important regulator of TR efficiency. TR was also reduced after PML induction with 4-OHT, with or without gentamicin, in AQP4 and MMuLV transduced cells (Fig. 2C,D). The decrease of TR efficiency in MMuLV reporter further suggest an antiviral role of PML-IV.

### Circumventing senescence decreases the fidelity of translation termination

**Fig. 3.**
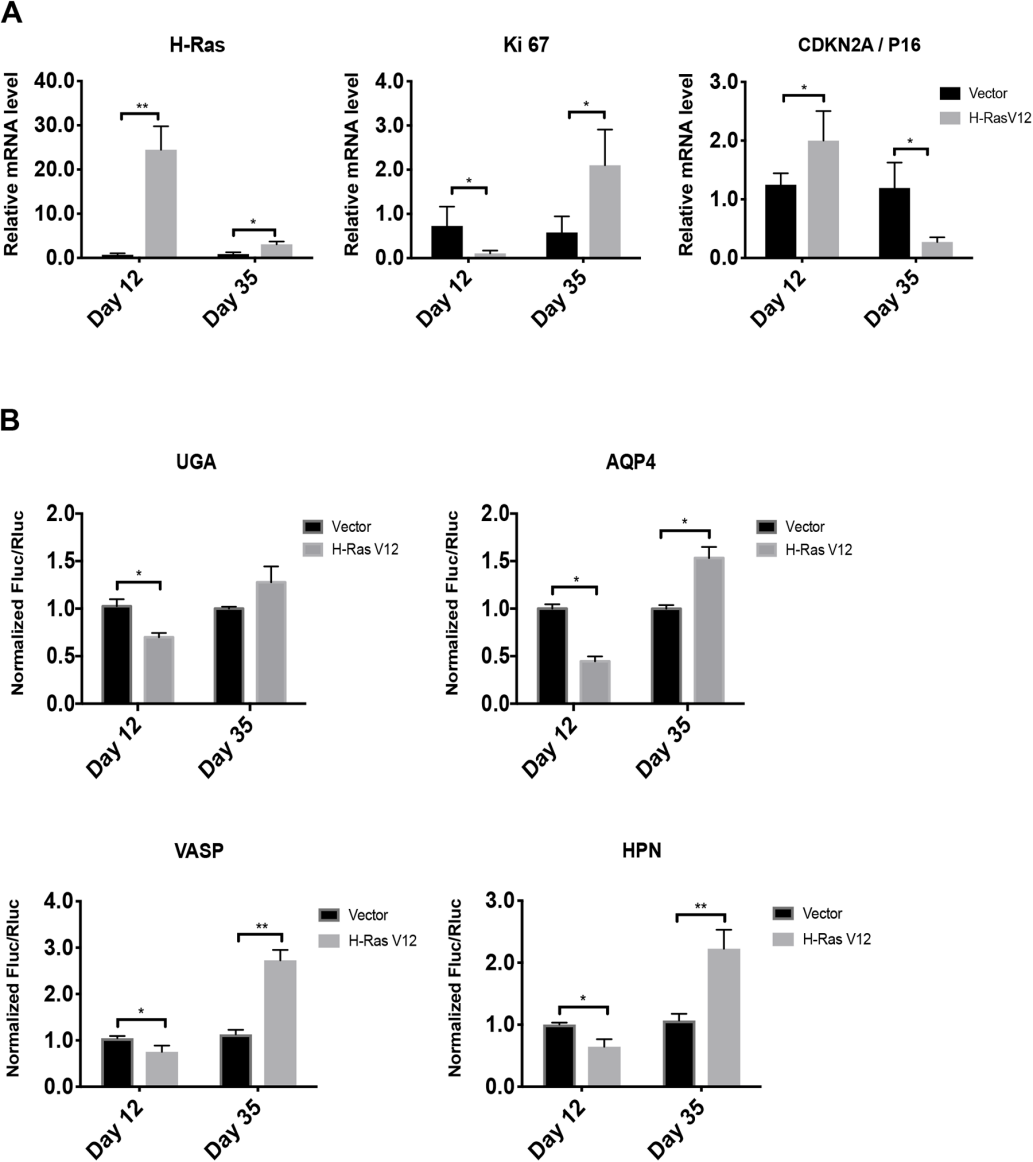
**Cells that bypassed-OIS show increased readthrough.** (A) RT-qPCR for H-RAS and senescent marker mRNAs were performed in IMR-90 cells at day 12 and 35 post infection with an empty vector (Vector) or H-RasV12 oncogene (H-RasV12). Data are normalized over TBP and HMBS, and presented as means relative to vector infected cells, *n*=3. B. Luciferase activities in non-senescent and senescent cells measured at day 12 and 35 post-infection in cells having the indicated reporters. Normalizations are presented as means relative to vector-infected cells from three independent experiments with technical triplicates for each experiment, except for UGA reporter, which are representative of two independent experiments with similar results. Error bars indicate SD of biological triplicates (AQP4, HPN, VASP) or technical triplicates (UGA). *=*P*<0.05, **=*P*<0.01 are significantly different, using two-tailed Student's *t*-test.

Although senescence in response to oncogenes includes a very stable cell cycle arrest, some cells escape from the process and progress towards malignant transformation. Cells that circumvent OIS display a gene expression profile typical of malignant cells and chromosomal aberrations (Deschenes-Simard et al., 2016). We sought to determine whether these cells also lose their tight control over TR. We obtained cell populations that bypassed OIS from long-term cultures (35 days) of IMR-90 cells expressing oncogenic *ras*. Cells that escaped senescence revert their morphology to that of normal growing cells (not shown) and expressed lower levels of oncogenic *ras* (Fig. 3A), indicating a mechanism that allow them to prevent pro-senescence *ras*/ERK signaling. This is consistent with our previous observation that decreasing ERK signaling accelerates senescence bypass in H-Ras-expressing cells (Deschenes-Simard et al., 2013, 2016). Cells that bypassed senescence also had lower levels of the CDK inhibitor p16INK4a and higher levels of the proliferation marker KI67 (Fig. 3A). We next measured TR in these cells using the UGA stop codon or the natural signals for AQP4 (Loughran et al., 2014), VASP and HPN. In all cases, the cells that bypassed senescence displayed an increased TR even higher than control non-senescent cells (Fig. 3B).

### E7 Oncoprotein increases TR

**Fig. 4.**
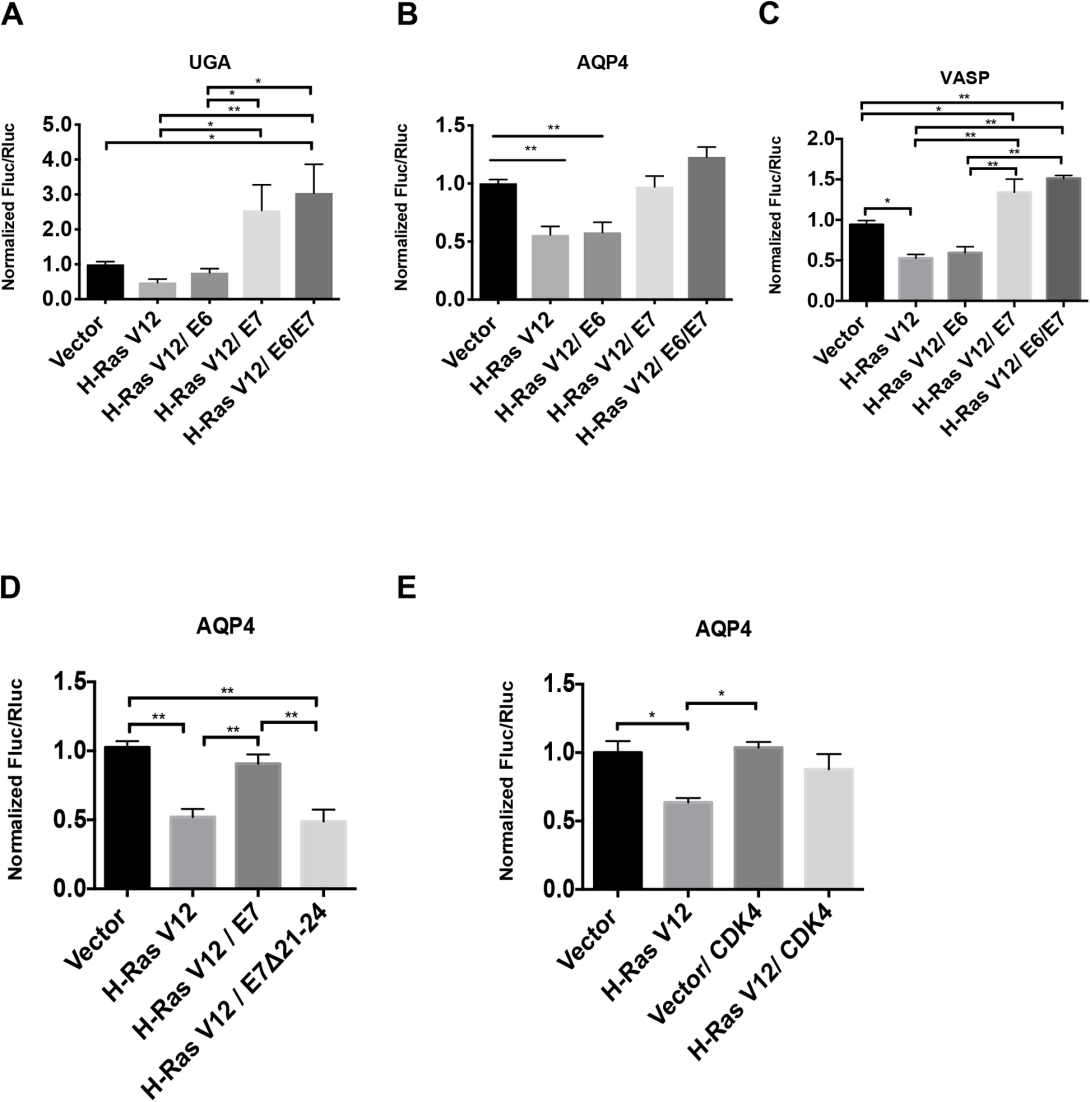
**RB pathway disruption increases readthrough.** (A–C) Readthrough efficiencies expressed as normalized Fluc/Rluc obtained from the luciferase reporters bearing a UGA stop codon within an artificial context (A) or a stop codon within natural contexts from either AQP4 (B) or VASP (B) that were expressed in IMR-90 cells. These cells also expressed HTERT, an empty vector (Vector) or H-RasV12 and an empty control vector (pLXSN), E6, E7 or E6/E7 oncogenes to study the readthrough efficiency in proliferating versus senescent versus transformed-like cells, *n*=3. (D) Relative readthrough luciferase values from IMR-90 cells transduced with the AQP4 readthrough reporter, with an empty vector (Vector) or H-RasV12 and an empty control vector (pLXSN), wild-type E7 or E7 Δ21-24 mutant oncogene. (E) IMR-90 cells were transduced with an empty vector or H-RasV12 or CDK4 and AQP4 luciferase reporters to study the readthrough efficiency variations in proliferating versus senescent versus cells overexpressing CDK4, *n*=3. A–E. Luciferase activities were measured in non-senescent and senescent cells at day 12 post-infection. Normalizations are presented as means relative to vector-infected cells, *n*=3 with technical triplicates for each experiment. One-way ANOVA with post-hoc Tukey HSD were performed. Error bars indicate SD of biological triplicates. Tukey HSD *P*-values indicate that *=*P*<0.05, **=*P*<0.01 are significantly different.

Since low translation fidelity and increased TR are associated to escape from senescence and malignant transformation (Marcel et al., 2013), we next investigated which tumor suppressor pathways activated in senescent cells could modulate TR. To investigate whether p53 and/or RB pathways are implicated in translation termination efficiency, we used E6 (inhibits p53) and E7 (inhibits RB) oncoproteins from HPV-16 virus. Transduction of E6 did not affect the TR levels in senescent cells. However, RAS-senescent cells infected with E7 oncoprotein or with both E6 and E7, showed a significant increase in TR at UGA stop codons and the natural readthrough signals from AQP4 and VASP (Fig. 4A–C). These results indicate that E7 targets, likely the pocket proteins family including RB, p107 and p130, mediate the readthrough decrease in senescent cells. We also knocked down RB mRNAs’ expression with shRNAs in H-Ras cells, but we failed to see the increase of TR levels (data not shown) that we observed in E7 expressing cells, suggesting that RB in coordination with p107 and p130 must influence the reduction in TR.

To further implicate the RB pathway in the control of TR in senescent cells, we used an in-frame deletion mutant of E7 whose interactions with RB, P107 and p130 are disrupted (E7 **Δ**21–24) (Helt and Galloway, 2001). As expected, H-Ras cells transduced with wild-type E7 showed a twofold increase in TR compared to senescent cells but the mutant E7 **Δ**21–24 failed to do so (Fig. 4D). Besides, the proliferating cells transduced with E7 presented a tendency to increase TR, while the TR was not affected in proliferating cells transduced with the mutant E7 **Δ**21–24 (Fig. S3A). In addition, RT-qPCRs were carried out to evaluate the expression of E2Fs target genes in cells transduced with E7 or the E7 **Δ**21–24 mutant. As expected E7 but not E7 **Δ**21–24 increased E2F target genes expression (Fig. S3B). Next, we used a retroviral vector for CDK4 overexpression to block pocket proteins activity in senescent cells (Sherr et al., 2016) and, as expected, we observed an increase in TR (Fig. 4E). Relative cell growth and RT-qPCRs show that CDK4 overexpression does not affect proliferation arrest markers at day 12 post infection in senescent IMR-90 cells (Fig. S3C,D). Taken together, these results indicate that the observed reduction in TR is not a simple consequence of the growth arrest of senescent cells and strongly suggest that the activation of the RB tumor suppressor pathway increases the fidelity of translation termination during OIS.

### RB pathway activation reduces TR

**Fig. 5.**
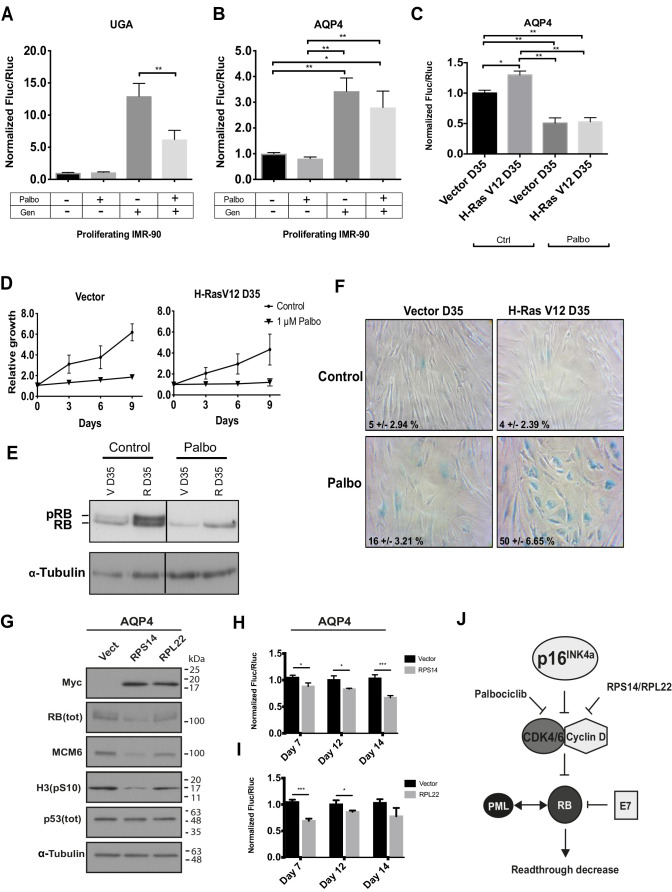
**RB pathway activation reduces readthrough.** (A,B) Normalized Fluc/Rluc ratios of IMR-90 cells transduced with luciferase reporters UGA (A) or AQP4 (B) and treated with vehicle and/or 1 μM of palbociclib (Palbo) and/or 900 μg/ml of gentamicin sulfate (Gen) for 5 days before luciferase measurements. Error bars indicate SD of three independent experiments, *n*=3. (C) Normalized Fluc/Rluc ratios of IMR-90 fibroblasts transduced with an empty vector (Vector) or H-RasV12 oncogene (H-RasV12) to induce OIS but cultured for 35 days until they bypassed the phenotype (D35) and with AQP4 luciferase reporter. Cells were treated with vehicle (Ctrl) or 1 μM of palbociclib (Palbo) for 5 days before measuring luciferase activities at day 35 post-infection. Normalizations are presented as means relative to vector-infected cells, *n*=3 with technical triplicates for each experiment. One-way ANOVA with post-hoc Tukey HSD. Error bars indicate SD of biological triplicates. Tukey HSD *P*-values indicate that *=*P*<0.05, **=*P*<0.01 are significantly different. (D) Growth curves of proliferating (Vector) and *ras* bypassed (H-RasV12 D35) IMR-90 cells treated with vehicle (Control) or 1 μM of palbociclib (Palbo) for 5 days are shown. Data are presented as means normalized to day 0 of each condition and error bars indicate SD of three independent experiments, *n*=3. (E) Immunoblots for total RB (Tot RB) (note: upper band represents phosphorylated RB: pRB) and alpha-tubulin from non-senescent (V D35) and *ras* bypassed cells (R D35) following treatments with vehicle (Ctrl) or 1 μM of palbociclib (Palbo) for 5 days. Blots are representative of three independent experiments with similar results. (F) SA-β-gal of proliferating (Vector D35) IMR-90 cells and IMR-90 cells that have by-passed the senescent stage (H-RasV12 D35) treated for 5 days with vehicle (Ctrl) or 1 μM of palbociclib (Palbo) and fixed at day 35 (D35) post-infection. Data were quantified from many fields within one experiment to represent the entire petri dish. Three independent cell counts up to a total of at least 100 cells are presented as the mean and SD of positive cells. (G) Immunoblots for indicated proteins at day 7 post infection with an empty vector (Vect), pBABE-RPS14(WT)-Myc (RPS14) or pBABE-RPL22(WT)-Myc (RPL22): Myc (Myc-tag), total RB [RB (tot)], MCM6, phosphorylated H3 on serine 10 [H3(pS10)], total p53 [p53(tot)] and alpha-tubulin. Blots are representative of three independent experiments with similar results. (H,I) Normalized Fluc/Rluc ratios of IMR-90 cells transduced with an empty vector (Vector), or pBABE-RPS14(WT)-Myc (RPS14, H), or pBABE-RPL22(WT)-Myc (RPL22, I), and with the luciferase reporter AQP4. Luciferase activities were measured at days 7, 12 and 14 post infection. Normalizations are presented as means relative to vector-infected cells, *n*=3 with technical triplicates for each experiment Unpaired *t*-tests with equal s.d. were performed. Error bars indicate SD of biological triplicates. *=*P*<0.05, **=*P*<0.01, ***=*P*<0.001 are significantly different, using two-tailed Student's *t*-test. (J) Schema showing the RB activation/inhibition factors that modulate readthrough.

Having demonstrated that RB pathway inhibition increased TR we next investigated whether activation of the RB pathway would inhibit TR. First, we used the CDK inhibitor palbociclib (Law et al., 2015). Treatment of proliferating fibroblasts with palbociclib reduced TR of the AQP4 reporter and gentamicin-stimulated TR at the UGA stop codon (Fig. 5A,B). Interestingly, palbociclib also reduces TR in cells that spontaneously escaped from senescence (Fig. 5C). As expected, palbociclib also inhibits cell proliferation and strongly activates the RB pathway in proliferating and H-Ras-escaped IMR-90 cells (Fig. 5D; Fig. S4) restoring their senescent phenotype (Fig. 5E,F).

We recently demonstrated that extra-ribosomal functions of RPS14/uS11 and RPL22/eL22 were linked to the regulation of the cell cycle and senescence. We found that these two proteins interact with the CDK4–Cyclin D1 complex, inhibiting its activity and consequently activating the RB pathway (Bury et al., 2021; Del Toro et al., 2019; Lessard et al., 2019; Lessard et al., 2018). These results suggest that RPS14/uS11 and RPL22/eL22 could, as palbociclib, affect TR. We transduced IMR-90 cells with Myc tagged RPL22/eL22 or RPS14/uS11 as well as with the AQP4 readthrough reporter and measured TR efficiency at day 7, 12 and 14 after transduction. The expression of RPL22/eL22 or RPS14/uS11 was confirmed by immunoblots against the Myc tag (Fig. 5G). We also measured biomarkers of senescence such as RB, the E2F target MCM6 and the mitosis marker phospho-H3 (Fig. 5G). Cells bearing RPS14/uS11 showed a 15% decrease of TR at days 7 and 12 after transduction, and 30% decrease of TR 14 days after transduction compared to control cells (Fig. 5H). In contrast, RPL22/eL22-transduced cells showed a peak of TR reduction at day 7 post infection, and a progressive recovery 12- and 14-days post-infection (Fig. 5I). We already reported that RPS14/uS11 is more efficient than RPL22/eL22 for inhibition of CDK4 and induction of senescence (Del Toro et al., 2019), explaining this difference. These results suggest that RPS14/uS11 haploinsufficiency in the 5q-syndrome (Ebert et al., 2008) and frequent RPL22/eL22 hemizygous gene deletions found in cancer (Ajore et al., 2017) could play a role in proteome diversity and tumorigenesis. Taken together, our results suggest that the RB pathway is implicated in translation termination accuracy (Fig. 5J).

### Therapy-induced senescence and TR

**Fig. 6.**
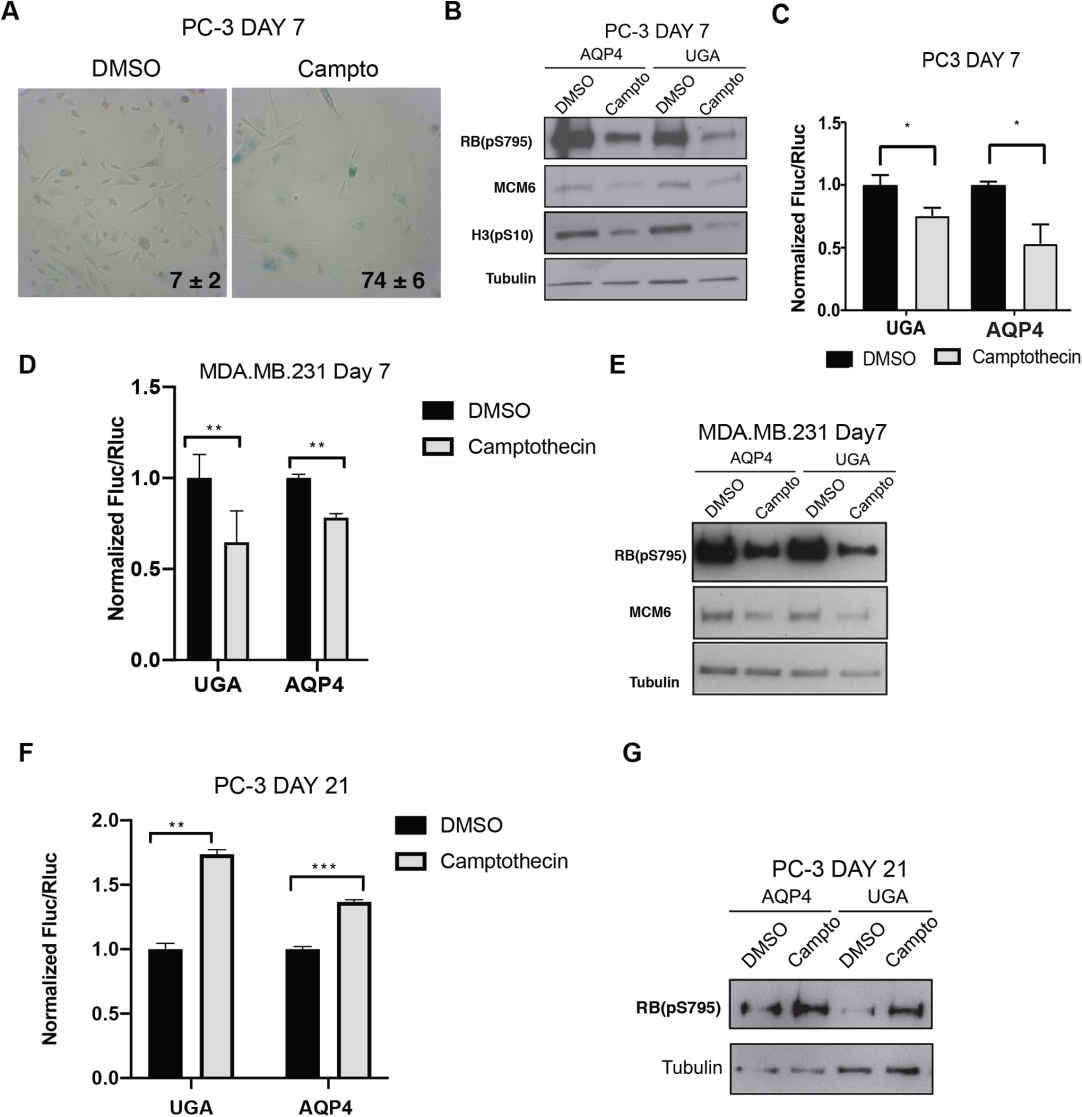
**Therapy-induced senescence reduces readthrough in cancer cells.** (A) SA-β-gal staining of PC-3 cells treated 24 h with vehicle and/or 100 nM of camptothecin and fixed 7 days later (day 7), the percent positive cells ±SD is shown in lower corner, *n*=3. (B) Immunoblots in PC-3 cells transduced with luciferase reporter (AQP4, UGA) and treated as in A for the proteins: RB(pS795) [phosphorylated RB on serine 795], MCM6, H3(pS10) [phosphorylated H3 on serine 10] and tubulin. Blots are representative of three independent experiments with similar results. (C) Normalized Fluc/Rluc ratios of indicated readthrough reporters in cells as in A. Normalizations are presented as means relative to vehicle treated cells from (UGA) *n*=4 and (AQP4) *n*=5 independent experiments each with technical triplicates. (D) Normalized Fluc/Rluc ratios in of MDA.MB.231 cells transduced with the luciferase reporters (UGA, AQP4) and treated as in A. Normalizations are presented as means relative to vehicle treated cells, *n*=3. (E) Immunoblots for phosphorylated RB at serine 795 [RB(pS795)], MCM6 and tubulin in cells as in D, *n*=3. (F) Normalized Fluc/Rluc ratios of indicated readthrough reporters in PC-3 cells as in A, 21 days post-treatment when the cell population bypassed senescence. For all luciferase assays, *n*=3, error bars indicate SD of technical triplicates of each experiment. *=*P*<0.05, **=*P*<0.01, ***=*P*<0.001 using two-tailed Student's *t*-test. (G) Immunoblots for phosphorylated RB at serine 795 [RB(pS795)] and tubulin in cells as in E, *n*=3.

To investigate whether a reduction in readthrough also applies to the senescent response induced by chemotherapeutic drugs in cancer cells (therapy-induced senescence: TIS) we treated PC3 prostate cancer cells with camptothecin for 24 h and confirmed that most of the cell population was senescent using the SA-β-Gal assay and western blots for phospho-RB, MCM6 and pH3(S10) (Fig. 6A,B). As found for OIS, TIS also reduced TR on both the UGA and the AQP4 reporters (Fig. 6C) and similar results were found in MDA.MB.231 breast cancer cells (Fig. 6D,E). TIS is not a stable phenotype (Guillon et al., 2019; Milanovic et al., 2018; Saleh et al., 2020). After 20 days in culture, a population of growing cells emerged from the TIS population and continued to grow without signs of senescence. These cells that bypass TIS are known to be more malignant (Milanovic et al., 2018; Saleh et al., 2020) and they have an increased TR (Fig. 6F) along with higher phosphorylation of RB (Fig. 6G).

### Senescence suppresses the expression of AGO1x, an AGO1 isoform generated by TR

**Fig. 7.**
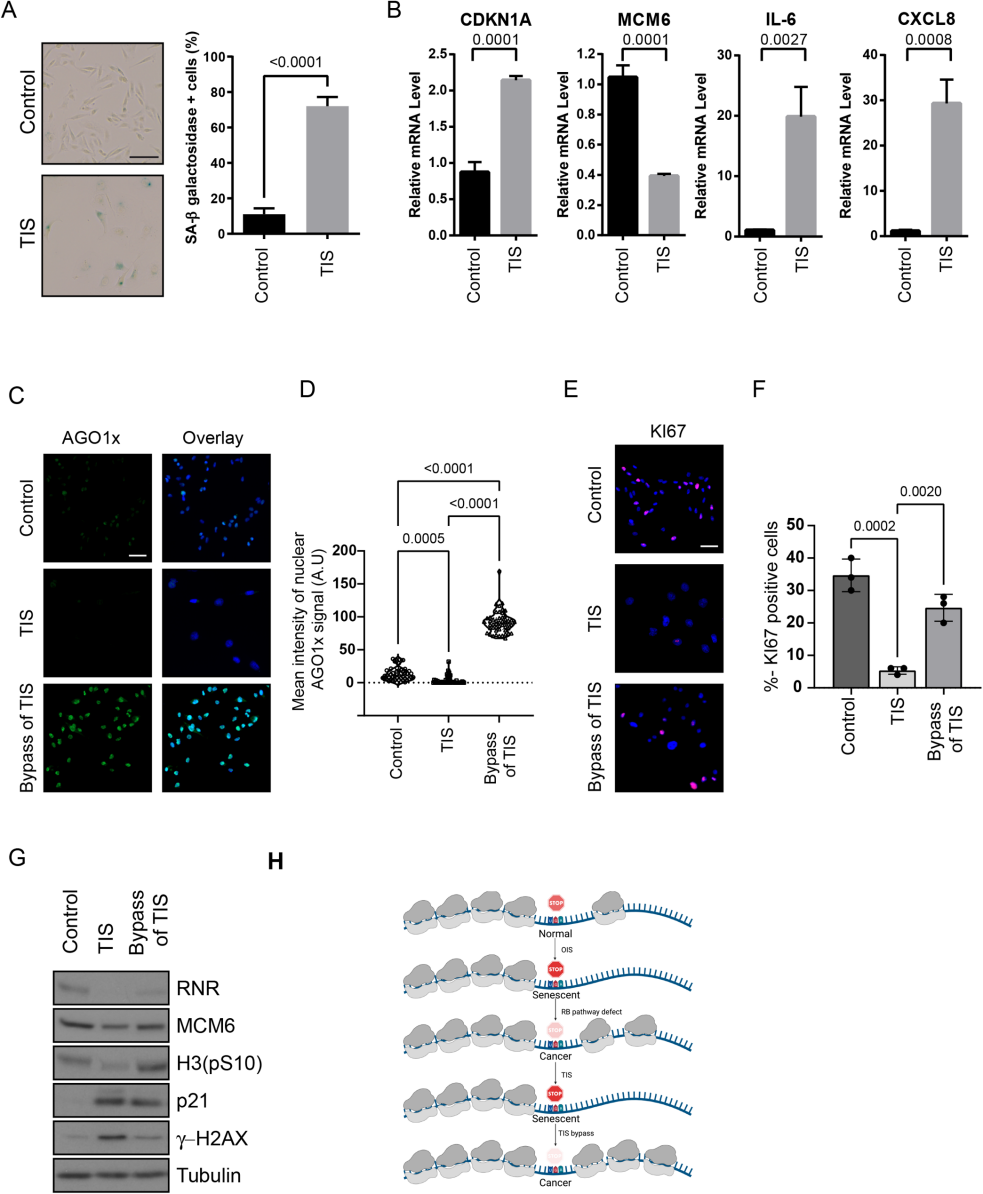
**Senescence affects endogenous TR target AGO1x.** (A) Images of senescence-associated β galactosidase assay and its quantification in MDA.MB.231 treated with 40 nM camptothecin (therapy-induced senescence (TIS)) or Vehicle (control) for 96 h. Data shows mean of three biological replicates and error bars show SD Students-*t*-test was performed and *P* value is indicated. (B) Relative mRNA levels of indicated mRNA in cells as in A. Data shows mean of three biological replicates and error bars show SD Students-*t*-test was performed and *P* value is indicated. (C) Immunofluorescence of AGO1x in MDA.MB.231 cells that were treated with 40 nM camptothecin (TIS) or Vehicle (control) or cells that were treated with camptothecin and left to recover for 14 days to allow bypass of senescence (Bypass of TIS). Scale bar=50 µm. A representative image of three biological replicates is shown. D. Quantification of results in (C). A minimum of 50 cells were scored for nuclear staining intensity using Image J. Statistical analysis was performed by ANOVA with Tukey post-test. A.U., arbitrary units. (E) Immunofluorescence of KI67 from MDA.MB.231 cells treated as in C. Scale bar=50 µm. A representative image of three biological replicates is shown. (F) Quantification of the percentage of cells expressing Ki67 shown in E. Statistical analysis was performed by ANOVA with Tukey post-test. (G) Immunoblot against the indicated proteins in MDA.MB.231 cells treated as in C. H3(pS10): phosphorylated Histone H3 at serine 10, and γH2AX: phosphorylated at serine 139 of histone H2AX variant. (H) Regulation of readthrough in senescent cells.

AGO1x is an isoform of AGO1 that is expressed by TR of the AGO1 mRNA (Eswarappa et al., 2014; Ghosh et al., 2020; Singh et al., 2019). AGO1x acts as an inhibitor of the miRNA pathway (Singh et al., 2019) and in breast cancer cells it is required to maintain cell proliferation through inhibition of interferon gene expression (Ghosh et al., 2020). Senescent cells upregulate interferon gene expression (Fridman and Tainsky, 2008; Frisch and MacFawn, 2020; Moiseeva et al., 2006) suggesting that a reduced TR of AGO1 mRNA may lead to low levels of AGO1x and high levels of interferon genes in these cells. To test this idea, we triggered therapy induce senescence (TIS) in MDA.MB.231 breast cancer cells with Camptothecin (Fig. 7A,B). As expected, the expression of AGO1x was reduced in these TIS cells (Fig. 7C) but was dramatically induced after cells escaped from senescence (Fig. 7C,D). Senescence and the escape from senescence was characterized using KI67 (Fig. 7E,F) and immunoblots for RNR (a repression target of p53), p21 (an activation target of p53), MCM6 (a repression target of RB), phospho-H3 (a marker of cell proliferation) and γH2AX (a marker of DNA damage) (Fig. 7G). These results demonstrate that senescence can lead to repression of cancer driver proteins like AGO1x by reducing TR.

## DISCUSSION

We show here that cellular senescence induced by oncogenic *ras*, the tumor suppressor PML, CDK4 inhibitors or chemotherapy reduces TR. Conversely, we also report that cells that bypass senescence have an increase in translational TR (Fig. 7H). The effect of senescence was demonstrated in a variety of reporters containing stop codons in a common context, within regions that stimulate endogenous TR and on an endogenous RT target, AGO1x. We propose a novel caretaker tumor suppressor activity for cellular senescence controlled mainly by the RB pathway in preventing TR and the generation of C-terminally extended proteins (Schueren et al., 2014; Stiebler et al., 2014). The potential for TR to confer advantages to cells in stress was previously reported in yeast cells expressing the prion PSI that enhances both TR and resistance to several stresses (True et al., 2004).

Senescence also reduced the stimulatory effect of aminoglycosides and the enhancer effect of CDX5-1 on TR (Manuvakhova et al., 2000). It has been reported that CDX5-1 potentiates G418-induced TR up to 180-fold compared to the aminoglycoside G418 alone (Baradaran-Heravi et al., 2016). Green and Goff reported that aminoglycosides can increase TR of the MMuLV gag-pol junction in HEK-293 cells (Green and Goff, 2015). However, using normal fibroblasts, we found that the same drugs did not have such effect. HEK-293 cells have an inactivation of the RB and PML tumor suppressor pathways due to expression of adenovirus E1A oncoprotein. In our normal cells, disruption of these pathways increases TR. It is thus plausible that in the context of an altered control in HEK293 cells, the MMuLV gag-pol junction TR is stimulated by aminoglycosides. In addition, Green and Goff used several aminoglycosides in their studies, gentamicin being the less active and its effects were reported as dose-independent and not consistent. Since gentamicin does increase TR in other stop codon contexts, we suggest that the pseudoknot structure of the viral signal may interfere with the binding or the action of gentamicin on the ribosome.

Our results indicate that translation termination tends to be inaccurate in cancer-like cells where the RB pathway is dysfunctional but is particularly efficient in senescent cells. Early work by Stanners and colleagues showed that SV40-mediated transformation increased mistranslation in mammalian cells (Pollard et al., 1982). SV40 encodes for the large T antigen that binds and inactivates the RB family of tumor suppressors (DeCaprio et al., 1988) like the E7 oncoprotein we used in this study. Recently, Ruggero et al. found that loss of SNORA24, a small nucleolar RNA that mediates pseudouridylation of rRNA, leads to bypass of RAS-induced senescence in the liver promoting hepatocellular carcinoma. Intriguingly, cells lacking SNORA24 had an increase in TR and mistranslations (McMahon et al., 2019). Also Diaz and colleagues found that translational fidelity is decreased in the most aggressive breast cancer cell lines (Belin et al., 2009). They also reported that the p53 tumor suppressor controls translational fidelity and in particular the translation of oncogenic proteins from IRES (Marcel et al., 2013). In our experimental system, the RB pathway was more important to control TR levels in OIS. Consistent with this notion, we show that TR was reduced after TIS in p53 mutant PC-3 and MDA.MB.231 cells. However, we did notice that inactivation of p53 in cells where RB is also inactivated further increased TR, suggesting also an important role for p53 (Fig. 4A,C). Taken together, the evidence indicates that preventing translational errors and TR is a tumor suppressor mechanism.

The RB tumor suppressor pathway may repress genes that modulate TR. TR occurs when near cognate tRNAs outcompete the translation termination factors eRF1/eRF3 (Beissel et al., 2019; Cassan and Rousset, 2001). RB represses tRNA expression (Gjidoda and Henry, 2013) and this could prevent TR by near cognate suppressor tRNAs. Also, the translation initiation factor eIF3 interacts with post-initiation ribosomes and controls TR (Beznosková et al., 2013, 2015). RB could repress the expression of eIF3 subunits or sequester them in PML bodies (Vernier et al., 2011). Consistent with the latter mechanism the eIF3 subunits EIF3K and EIF3E (Int-6) localize to PML bodies (Morris-Desbois et al., 1999; Salsman et al., 2013). Viral proteins such as HTLV-I Tax can delocalize EIF3E from PML bodies (Desbois et al., 1996), an event that could facilitate TR.

Of special interest for a plausible pharmacological modulation of readthrough levels, we found that the CDK4 inhibitor palbociclib was able to reduce TR and re-induce a stable cell cycle arrest in cells that bypassed senescence. If the phenotypic plasticity conferred by C-terminal extended proteins plays a causal role in the origin of human cancers or their resistance to chemotherapy, palbociclib could be used to halt the progression of these lesions.

## Supplementary Material

Supplementary information
