# Supplementary material for: Cellular senescence limits translational readthrough
Source: Biol Open. 2021 Dec 2;10(12):bio058688. doi: 10.1242/bio.058688 (PMC8649927; doi:10.1242/bio.058688)
Supplement: Supplementary information [file biolopen-10-058688-s1.pdf]

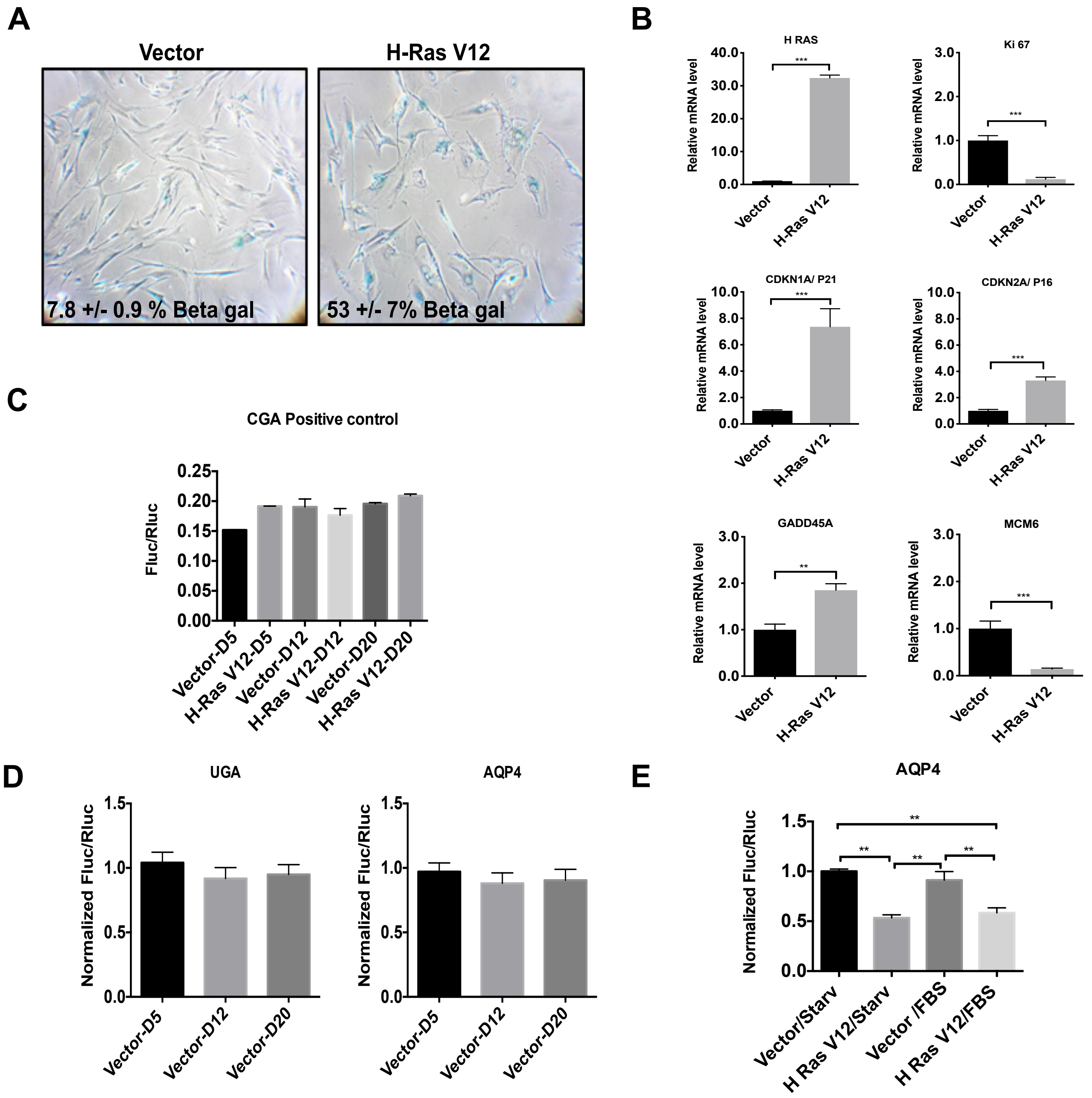

Fig. S1.Readthrough is reduced in OIS.A. SA-β-gal of proliferating (Vector) and senescent IMR90 (H-RasV12 oncogene) cells, fixed at day 12 post-infection. Data were quantified from many fields within one experiment to represent the entire petri dish. Three independent cell counts up to a total of at least 100 cells are presented as the mean and SD of positive cells. **B.** RT-qPCRs for senescent marker mRNAs were performed in IMR-90 cells at day 12 post-infection for cells as in **(A)**. Data are normalized over TBP and HMBS, and presented as means relative to vector infected cells. Error bars indicate SD of technical triplicates. ** = $p<0.01$, *** = $p<0.001$ are significantly different, using two-tailed Student's t-test. **C.** Luciferase activities from non-readthrough control (CGA-Positive control) in proliferating (Vector) and H-RasV12 senescent cells measured at days 5 (D5), 12 (D12) and 20 (D20) post-infection. Assays are representative of 2 independent experiments with similar results with technical triplicates for each experiment. One-way ANOVA with post-hoc Tukey HSD were performed. Error bars indicate SD of technical triplicates. **D.** Normalized Fluc/Rluc ratios indicate that readthrough level does not change in non-senescent cells (Vector) at day 5 (D5), 12 (D12) or 20 (D20) post-infection. Error bars indicate SD of biological triplicates. **E.** Luciferase activities from AQP4 reporter were measured at day 12 post-infection. Normalized Fluc/Rluc ratios from non-senescent (Vector) and senescent cells (H-RasV12), starved (Starv) or supplied with fetal bovine serum (FBS) are represented. Starvation was performed for a week to induce quiescence. Data are presented as means relative to empty vector-infected cells, which are representative of 2 independent experiments with similar results with technical triplicates for each experiment. One-way ANOVA with post-hoc Tukey HSD were performed. Error bars indicate SD of technical triplicates. Tukey HSD p-values indicate that * = $p<0.05$, ** = $p<0.01$, *** = $p<0.001$ are significantly different.

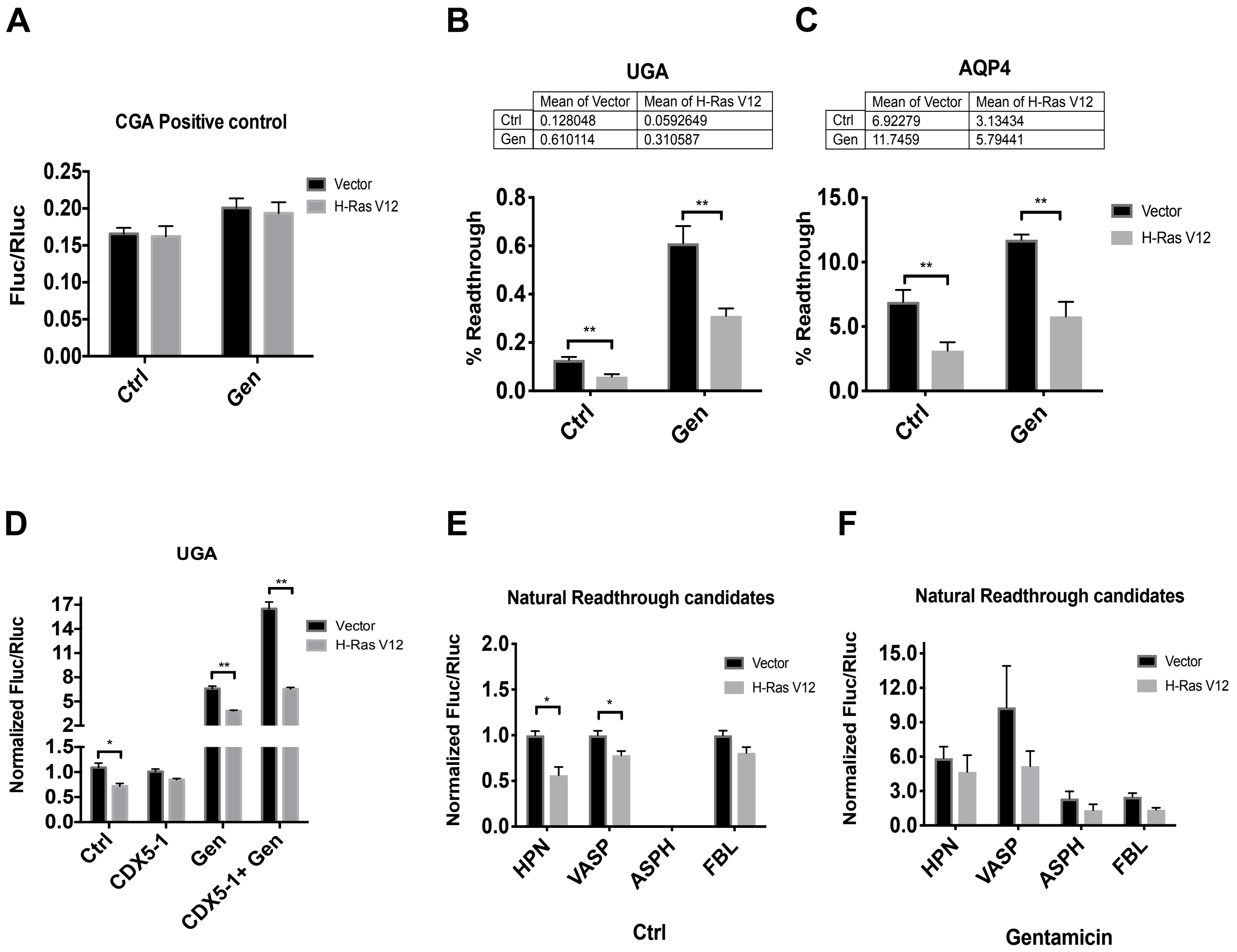

Fig. S2. Senescent cells are resistant to gentamicin-induced readthrough.A. IMR-90 cells were transduced with an empty vector
(Vector) or H-RasV12 oncogene to induce OIS and with indicated non-readthrough control luciferase reporter. Cells were treated with vehicle (Ctrl) or with 900 µg/ml of gentamicin sulfate (Gen) 24 hours before measuring luciferase activities at day 12 post-infection. Error bars indicate SD of biological triplicates. **B-C.** The percent of readthrough was determined by dividing Fluc/Rluc ratio from UGA or AQP4 luciferase reporters by Fluc/Rluc ratio from non-readthrough controls multiplied by 100. Error bars indicate SD of biological triplicates. ** = p<0.01 is significantly different, using two-tailed Student's t-test. **D.** IMR-90 cells were transduced with an empty vector (Vector) or H-RasV12 oncogene to induce OIS and with a UGA luciferase reporter. Cells were treated with vehicle (Ctrl) and/or 600 µg/ml of gentamicin sulfate (Gen) and/or 25 µM of CDX5-1 72 hours before measuring luciferase activities at day 12 post-infection. Error bars indicate SD of biological triplicates. * = p<0.05, ** = p<0.01 are significantly different, using two-tailed Student's t-test. **E.** A stop codon within natural contexts from VASP, ASPH, HPN and FBL, was inserted in the intercistronic region of Rluc-Fluc luciferase reporter. IMR-90 cells were transduced with an empty vector (Vector) or H-RasV12 oncogene to induce OIS and with luciferase reporters. Luciferase activities were measured in non-senescent and senescent cells at day 12 post-infection. Error bars indicate SD of biological triplicates. * = p<0.05 is significantly different, using two-tailed Student's t-test. **F.** Cells as in **(E)** were treated with 900 µg/ml of gentamicin sulfate (Gen) 24 hours before measuring luciferase activities at day 12 post-infection. Normalized Fluc/Rluc ratios indicate the efficiency of readthrough. Normalizations are presented as means relative to vector-infected cells from three independent experiments with technical triplicates for each experiment. Unpaired Student's t-test with equal SD were performed. Error bars indicate SD of biological triplicates.

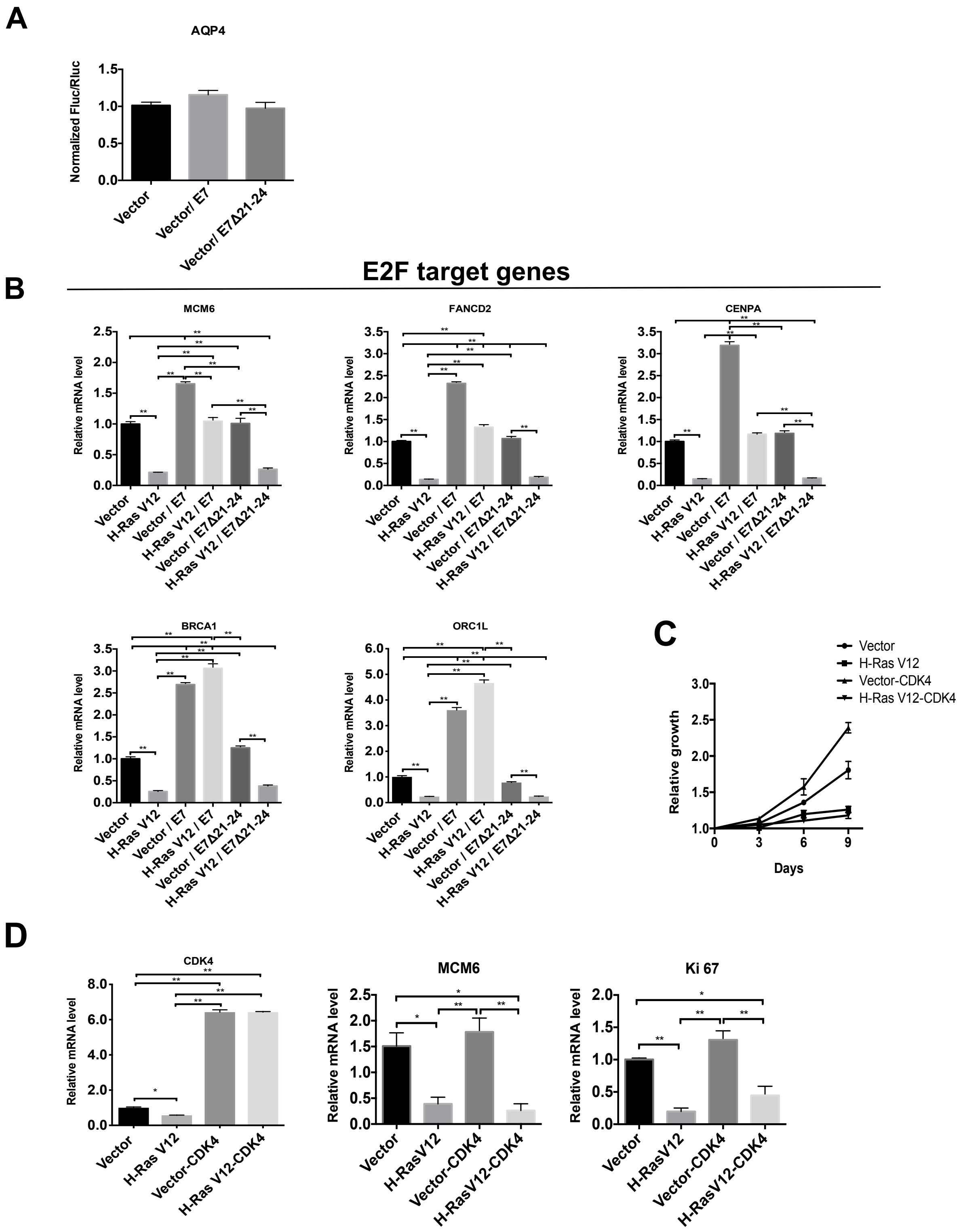

Fig. S3. RB pathway disruption affects the efficiency of readthrough. A. IMR-90 cells were transduced with luciferase reporter AQP4 and an empty vector (Vector), wild-type E7 or E7 Δ21-24 mutant oncogene. Luciferase activities were measured at day 12 post-infection. Normalized Fluc/Rluc ratios indicate the efficiency of readthrough. Normalizations are presented as means relative to vector-infected cells from three independent experiments with technical triplicates for each experiment. Error bars indicate SD of biological triplicates. **B.** Representative RT-qPCR from E2Fs' target mRNAs were performed in IMR-90 cells transduced with an empty control vector (pLXSN), wild-type E7 or E7 Δ21-24 mutant oncogene, and with an empty vector (Vector) or with H-RasV12 oncogene to induce OIS at day 12 post-infection. Data are normalized over TBP and HMBS, and presented as means relative to vector infected cells. Experiments were done three times with technical triplicates for each experiment. Error bars indicate SD of technical triplicates. Tukey HSD p-values indicate that ** = $p<0.01$ is significantly different. **C.** Growth curves of non-senescent (Vector) and H-RasV12 senescent IMR-90s overexpressing or not CDK4. Data are presented as means normalized to day 0 of each condition. Assays are representative of 2 independent experiments with similar results with technical triplicates for each experiment. Error bars indicate SD of technical triplicates. **D.** RT-qPCRs for CDK4, MCM6 and KI67 mRNAs were performed in IMR-90 cells as in **(C)** and at day 12 post-infection. Data are normalized over TBP and HMBS, and presented as means relative to vector infected cells from three independent experiments with technical triplicates for each experiment. One-way ANOVA with post-hoc Tukey HSD were performed in **(A, B, D)** and Unpaired Student's t-test with equal SD was performed in **(C)**. Error bars indicate SD of biological triplicates. Tukey HSD p-values indicate that * = $p<0.05$, ** = $p<0.01$ are significantly different.

Biology Open • Supplementary information

Biology Open (2021): doi:10.1242/bio.058688: Supplementary information

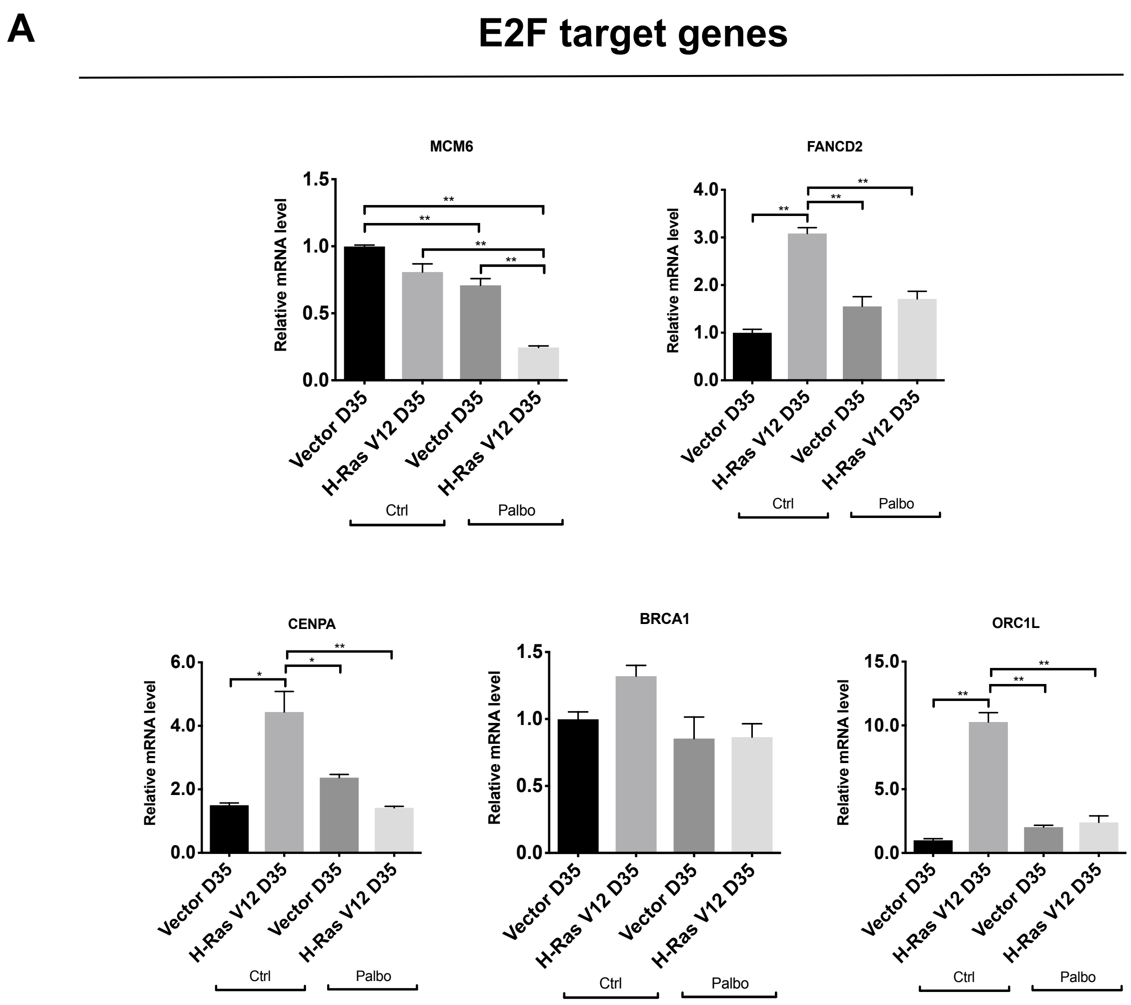

Fig. S4. E2Fs target genes.A. RT-qPCR for E2Fs' target mRNAs were performed in IMR-90 at day 35 post-infection with an empty vector (Vector D35) or with the oncogene H-RasV12 (H-RasV12 D35) and treated with vehicle (Ctrl) or with 1 µM of palbociclib (Palbo) for 5 days before cell lysis. Data are normalized over TBP and HMBS, and presented as means relative to vector infected cells from three independent experiments with technical triplicates for each experiment. One-way ANOVA with post-hoc Tukey HSD were performed. Error bars indicate SD of technical triplicates. Tukey HSD p-values indicate that * = p<0.05, ** = p<0.01 are significantly different.

Table S1. PCR cloning primers

Name	5' forwad primer (5'-3')	3' reverse primer (5'-3')
pMSCV-RLUC-FLUC	GGAAGAAAGCGGCCGCTTCAGATCCGCTAGAGCC	TGGATGCATGAATTTCGTCATCGCTGAATAC
UGA STOP CODON	CCAAGCTTGCACCAGTGACCAGCAAGGTGTACG	CGTACACCTTGCTGGTCACTGGTGCAAGCTTGG

Table S2. Readthrough region

Gene name			Readthrough context
AQP4	Aquaporin 4	NM_001650.4	ATCTGGAGAGGTATTGTCTTCAGTA**TGA**CTAGAAGATCGCACTGAAAGCAGACAAGACTCCTTAGAA
ASPH	Aspartate beta hydroxylase	NM_004318.3	AGAACAGCAGGAAGTACCACCAGATACT**TAA**AGCTTCAAAAAGACTGCCCCTACCACCGGGCC
FBL	Fibrillarin	NM_001436.3	GTACAGGCCACCCCCCAAGGTGAAGAAC**TGA**AGTTCAGCGCTGTCAGGATTGCGAGAGATGGG
HPN	Hepsin	NM_182983.2	TCGAGGCCAGCGGCATGGTGACCCAGCTC**TGA**CCGGTGGCTTCTCGCTGCGCAGCCTCCAGGG
MMuLV	Moloney Murine Leukemia Virus		C**TAG**GGAGGTCAGGGTCAGGAGCCCCCCCTGAACCCAGGATAACCCTCAAAGTCGGGGGGCC
VASP	Vasodilator-stimulated phosphoprotein	NM_003370.3	GGAGCTGAGGAAGCGGGGTTCTCCC**TGA**CCACAGGGACCCAGAAGACCCGCTTCTGGGCCCCA

Biology Open • Supplementary information

Table S3. Readthrough candidates

Readthrough code	Gene name	peptides found by MS analysis			peptides « confi	stop- DNA 3'UTR	RTP (readthough propensity)(Ref 22)	RTP Linfs3 (Ref 22)
RT_012331.1	FAM19A5 family with sequence similarity 19 (chemokine (C-C motif)	APSEDGLRLR	NP_001076436		3608	[illegible]	0.309	0.646
RT_006854.1	ZNF182 zinc finger protein 182	YGSKFSLSSFHLSHNPK	NP_001171570.1, NP_001		3732	[illegible]	0.143	0.215
RT_020801.1	ACADS acyl-CoA dehydrogenase, C-2 to C-3 short chain	LGGPAGAPWGPQMGSVLPPR	NP_000008.1, N		8117	[illegible]	-0.077	0.066
RT_017649.1	ACSL5 acyl-CoA synthetase long-chain family member 5	VLKYLPAHCALLVR	NP_976313.1, NP_97631		690	[illegible]	-0.08	-0.226
RT_009441.1	ADCK2 aarF domain containing kinase 2	AGGHSQEPLLWQLGR	NP_443085.2	XP_01151	464	[illegible]	0.237	0.065
RT_013024.1	ADCY7 adenylate cyclase 7	GLLLDSEK	NP_001105.1	XP_01152	9511	[illegible]	-0.029	0.028
RT_020165.1	ADGRE5 adhesion G protein-coupled receptor E5	MVLDGPAAPVATAALYTK	NP_510966.1, N		6737	[illegible]	0.084	0.12
RT_004627.1	AES amino-terminal enhancer of split	LGGGTEGRQR	NP_945320.1, N	XP_00672	402	[illegible]	0.232	0.107
RT_024040.1	AGAP2 ArfGAP with GTPase domain, ankyrin repeat and PH domain 2	EGLVHGR	NP_055585.1, N	XP_00526	6563	[illegible]	0.059	0.072
RT_009047.1	AK1 adenylate kinase 1	QRWSRFPSSEPRPTPS	NP_000467.1,	XP_01151	5273	TAGcaacgctggagccgcttccccagctcagagccccgccccaccccgtcctga	0.054	0.424
RT_002208.1	AKAP8L A kinase (PRKA) anchor protein 8-like	PELGAGGAR	NP_001278407	XP_00525	924	TGAcccgagctcggggcgggcggagcccgcgtggccgaagctggaaaccaaacctaa	0.42	0.432
RT_017612.1|	alpha-(1,3)-fucosyltransferase 9	NFSSLAHLINILMR	NP_006572.2	XP_01153	652	[illegible]	-0.081	-0.089
RT_022128.1	AMOT angiomotin	SRAADYQQK	NP_573572.1, N	XP_01152	3141	TAAacggccaaatcaagagctgcagattatcagcaaaaatgcttttaa	0.051	-0.11
RT_021756.1	ANKLE2 ankyrin repeat and LEM domain containing 2	EGRVICLLLNCQK	NP_055929.1,	XP_00526	2085	[illegible]	-0.028	-0.215
RT_002681.1	ANKRD13D ankyrin repeat domain 13 family, member D	AIAPGGLARPLPAR	NP_997237.2,	XP_01154	10572	[illegible]	0.041	0.066
RT_001281.1	ANO10 anoctamin 10	GLCLCHTCHGGRGGTRGSIVAPEPF	NP_001191762	XP_01153	7239	[illegible]	0.236	0.257
RT_005833.1	APLP1 amyloid beta (A4) precursor-like protein 1	AQTSPLPGAPEPQLPA	NP_001019978.1, NP_005		2785	[illegible]	0.271	0.432
RT_021610.1	APPL2 adaptor protein, phosphotyrosine interaction, PH domain and l	LTCACGGR	NP_060641.2, N	XP_00671	20075	[illegible]	0.027	0.147
RT_023876.1	AQP12B aquaporin 12B	ALGSSKAAARSPVAR		XP_01150	9755	[illegible]	0.214	0.066
RT_007575.1	ARHGAP17 Rho GTPase activating protein 17	RTWRPPLK	NP_060524.4, N	XP_01154	3300	[illegible]	0.319	0.437
RT_018159.1	ARHGEF1 Rho guanine nucleotide exchange factor 1	IGGLFFNIYYLR		XP_00525	8679	[illegible]	0.186	0.382
RT_023604.1	ARPP21 cAMP-regulated phosphoprotein 21kDa	KLQMERNSK	NP_001254545	XP_00671	4165	[illegible]	0.28	0.373
RT_026723.1	ASB14 ankyrin repeat and SOCS box containing 14, predicted transcri	SSSAWEAEAGGLLHLGRSR		XP_00526	2744	[illegible]	0.161	0.151
RT_011768.1	ASPH aspartate beta-hydroxylase	KTAPTTTGGPA	NP_001158227.1, NP_001		5997	[illegible]	0.123	0.033
RT_021372.1	ASTN2 astrotactin 2	GRVLPSVSTETLHGR	NP_054729.3, N		235	[illegible]	0.001	0.028
RT_013285.1	ATG9A autophagy related 9A	DSSLIPR	NP_076990.4, NP_00107		11636	[illegible]	0.105	0.162
RT_006517.1	ATP10D ATPase, class V, type 10D	DFEEVSLQAR	NP_065186.3,	XP_00524	2260	[illegible]	0.203	0.444
RT_003127.1	ATP11C ATPase, class VI, type 11C	QNPNLELPMLLSYK	NP_775965.2	XP_01152	3127	[illegible]	0.129	0.152
RT_007887.1	ATP13A1 ATPase type 13A1	CLPEMAVLVPTAHPGCR		XP_01152	666	[illegible]	-0.032	0.056
RT_027025.1	ATP6V0B ATPase, H+ transporting, lysosomal 21kDa, V0 subunit b	LGVQGPPCTPLLLR	NP_004038.1, NP_00103		2970	[illegible]	0.123	0.127
RT_025167.1	BCORL1 BCL6 corepressor-like 1	LQNEAIIR	NP_068765.3	XP_00526	6940	[illegible]	0.176	0.341
RT_020389.1	C10orf105 chromosome 10 open reading frame 105	WGVHKHAPTSP	NP_001161862	XP_01153	9533	[illegible]	0.056	-0.113
RT_023564.1	C1GALT1 core 1 synthase, glycoprotein-N-acetylgalactosamine 3-beta	KKIMNEQR	NP_064541.1	XP_00524	1008	[illegible]	0.112	0.056
RT_024012.1	C1orf186 chromosome 1 open reading frame 186	IPNIFNGVQFSMDSYI	NP_001007545	XP_00527	10908	[illegible]	0.197	0.382
RT_021492.1	C1S complement component 1, s subcomponent	YIPPASPR	NP_958850.1, N	XP_00525	4939	[illegible]	0.001	-0.075
RT_007816.1	C22orf15 chromosome 22 open reading frame 15	GDGLHTVVR	NP_872326.2,	XP_00526	8506	[illegible]	0.116	-0.15
RT_019768.1	C22orf23 chromosome 22 open reading frame 23	LTAPSAEK; QSPSIPTRTCV	NP_001193991	XP_00526	1593	[illegible]	0.131	-0.134
RT_013060.1	C5orf60 chromosome 5 open reading frame 60	VTGITGLCHR		XP_01153	7069	[illegible]	0.364	0.122
RT_024351.1	CACNA1A calcium channel, voltage-dependent, P/Q type, alpha 1A su	THPTRGAAQRPR	NP_001120694.1, NP_075		2949	[illegible]	-0.086	-0.112
RT_018787.1	CAPN1 calpain 1, (mu/l) large subunit	QPRPPVPSPPSHHR	NP_001185797	XP_00671	1683	[illegible]	0.169	0.119
RT_005409.1	CASK calcium/calmodulin-dependent serine protein kinase (MAGUK f	ASLLSALCQQVMVPR	NP_001119527	XP_00672	1200	[illegible]	-0.161	-0.156
RT_010840.1	CCDC58 coiled-coil domain containing 58	REILFFFFFFFFLRTGSSHK	NP_001017928	XP_00524	3768	[illegible]	0.285	0.259
RT_004671.1	CCL3 chemokine (C-C motif) ligand 3	GVQKLR	NP_002974.1, N		287	[illegible]	0.291	0.028
RT_012415.1	CCNH cyclin H	PFEVDFSMLTNQEK	NP_001230.1, NP_00118		7561	[illegible]	0.402	0.48
RT_004395.1	CD72 molecule	NWTVPGK		XP_00671	1058	[illegible]	0.231	0.147
RT_016795.1	CD82 molecule	TLLPILTESRGLSGA	NP_002222.1, N	XP_00671	4030	[illegible]	0.065	0.119
RT_006924.1	CDH23 cadherin-related 23	FILEGGK	NP_001165402	XP_01153	7762	[illegible]	0.136	0.035
RT_019066.1	CDK2AP2 cyclin-dependent kinase 2 associated protein 2	LDLSGHCRAPASPWPSSR	NP_005842.1, N		4819	[illegible]	0.153	0.152
RT_008545.1	CEACAM4 carcinoembryonic antigen-related cell adhesion molecule	KEGALWLFWTHLG		XP_01152	3977	[illegible]	-0.015	-0.122
RT_022767.1	CEP57L1 centrosomal protein 57kDa-like 1	QNSKTVTLMNFVSETLNCLKWF	NP_001077004	XP_00526	8129	[illegible]	0.29	0.468
RT_002593.1	CFAP54 cilia and flagella associated 54	ISYTRDDKTEK		XP_01153	1470	[illegible]	0.333	0.666
RT_023936.1	CFHR3 complement factor H-related 3	GSIVTLNVCPTSTFPLLTLMVSK	NP_066303.2, NP_00116		9546	[illegible]	0.059	-0.059
RT_003463.1	CHRNA1 cholinergic receptor, nicotinic, alpha 1	AENELSLALPWNLPEQRRAGEEDLS	NP_000070.1, N		15187	[illegible]	0.219	0.292
RT_021669.1	CLEC12A C-type lectin domain family 12, member A	SVGGGGSRL	NP_001193939	XP_01151	2968	[illegible]	0.238	0.119
RT_010570.1	COG3 component of oligomeric golgi complex 3	SLAVCRTPR	NP_113619.2,	XP_01153	3531	[illegible]	0.213	0.114
RT_016946.1	COL1A1 collagen, type I, alpha 1	TPSIPTWLPPTQPTFPPTR	NP_000079.2	XP_01152	3390	ctga	0.046	-0.078
RT_004138.1	COL4A4 collagen, type IV, alpha 4	RMRNSPTR	NP_000083.3,	XP_00524	886	[illegible]	0.2	0.215
RT_014034.1	CORO2A coronin, actin binding protein, 2A	AETSALLTLR	NP_438171.1, N	XP_01151	20475	[illegible]	0.259	0.292
RT_024782.1	CPS1 carbamoyl-phosphate synthase 1, mitochondrial	HPSPIIK	NP_001116105	XP_01150	5966	[illegible]	0.222	0.215
RT_011692.1	CREB3L4 cAMP responsive element binding protein 3-like 4	AGTDLPGPLPDHK	NP_570968.1, N	XP_00671	671	[illegible]	0.075	0.008
RT_021761.1	CUX1 cut-like homeobox 1	GAAALGRAAR	NP_001189472	XP_01151	2074	[illegible]	0.237	0.028
RT_013213.1	CYB561 cytochrome b561	RLQDCRLR	NP_001906.3, N	XP_00525	12262	[illegible]	0.292	0.156
RT_019282.1	CYB5D2 cytochrome b5 domain containing 2	AVASSVNNTQRALPST	NP_001241685	XP_01152	4632	[illegible]	0.057	-0.112
RT_011989.1	CYGB cytoglobin	APVNQKHMAQVPQHAARPALPTLP		XP_01152	12296	[illegible]	0.393	0.658
RT_016292.1	DAAM2 dishevelled associated activator of morphogenesis 2	PGELATQEAGRQGLVR	NP_001188356	XP_00671	12479	[illegible]	0.226	0.373
RT_018283.1	DACH1 dachshund family transcription factor 1	VLLRELPEFCVLMFFK	NP_004383.4, N	XP_01153	5689	[illegible]	0.121	0.44
RT_013666.1	DENND2A DENN/MADD domain containing 2A	LGSSLDEDLGAAWEDSPRKGAR	NP_056504.3	XP_00525	6495	[illegible]	0.12	0.536
RT_013306.1	DGKD diacylglycerol kinase, delta 130kDa	RLASWALLAWPR	NP_690618.2, N	XP_01151	2001	[illegible]	0.227	0.151
RT_022377.1	DHODH dihydroorotate dehydrogenase (quinone)	LSSVPWSDHK	NP_001352.2,	XP_00525	13137	[illegible]	0.035	0.345
RT_007395.1	DOCK11 dedicator of cytokinesis 11	GNADVRDNETDLSQEYLELCK	NP_653259.3	XP_00526	4740	[illegible]	0.306	0.345
RT_023489.1	DOCK4 dedicator of cytokinesis 4	TFSVGTLITYSAPSMNGIK	NP_055520.3,	XP_01151	786	[illegible]	0.054	0.17
RT_013182.1	DOCK8 dedicator of cytokinesis 8	EKPSSFVETVALQPWR	NP_982272.2, N	XP_01151	2611	[illegible]	0.054	0.103
RT_017352.1	DOHH deoxyhypusine hydroxylase/monooxygenase	SSPAELWR	NP_001138637	XP_01152	1607	[illegible]	0.047	-0.103
RT_009530.1	DPPA4 developmental pluripotency associated 4	ATSMVINGRK	NP_060659.3	XP_01151	23756	[illegible]	0.046	-0.134
RT_024102.1	DRAM1 DNA-damage regulated autophagy modulator 1	NSVSLSECR	NP_060840.2,	XP_00526	626	[illegible]	0.267	0.437
RT_014648.1	DUSP18 dual specificity phosphatase 18	AIPRAPALESEVQIYC	NP_689724.3, N	XP_00672	4973	[illegible]	0.109	0.066
RT_024715.1	DUSP22 dual specificity phosphatase 22	LGRGAVVVADEDR	NP_001273484	XP_01151	3721	[illegible]	0.23	0.307
RT_006993.1	EDN3 endothelin 3	FPPLYRQEVNLPGAEHPPK	NP_996917.1, N	XP_01152	11113	[illegible]	0.153	-0.258

Biology Open • Supplementary information

RT_023670.1	EFCAB6 EF-hand calcium binding domain 6	TPLLCAQRDDSHR	NP_942153.1, N	XP_00526	10512	TAGacacccctgctgtgtgcccagcgggacgacagcccacagggcctgtttcaaggctaataaaatcctataa	-0.017	0.162
RT_001248.1	EPHA1 EPH receptor A1	TLLCTPENLFVLK	NP_005223.4	XP_00671	5193	TGAtccctcctctcaccccatgcccaatcagggtgcaaggagcaaggacggggccaaggtcgctcatggtcactccctgc<br>gccccttcccacaacctgccagactaggctatcggtgctgcttctgcccactttcaggagaaccctgctctgcaccccag<br>aaaacctctttgttttaaaagggaggtgggggtag	0.158	0.103
RT_026532.1	ERCC4 excision repair cross-complementation group 4	TVMAVFLSHACTFQRLLAR	NP_005227.1,	XP_01152	10931	TGAacagtgatggctgttttcttatcccatgcctgtacttttcagcggctccttgccagacatcataggtcattattaattattggtttgctatttcattcttttccaatgctcttaatgattgtacggtggaccagaagccaggattcctctctgaactctgcagttaggcatcacttgaacttgcctgtgcctgctctttt	0.218	0.384
RT_003934.1	ESRP1 epithelial splicing regulatory protein 1	MYGDLETSR	NP_001116297		8815	TGAaagatgtatggtgatcttgaaacctccagacacaagaaaacttctagcaaattcaggggaagtttgtctacactcag<br>gctgcagtattttcagcaaacttga	0.122	0.056
RT_002612.1	EVPL envoplakin	TPLASEQPHPR	NP_001979.2	XP_01152	9163	TGAcacgggccaaggagccagtggggaagtgcgtgtgttgggccaggtaggatacgtacacctcttgcctcagagcagcc<br>tcatcccaggcagtgggtcttccctctgtccaaccactgttttattattttactaacatggtga	0.216	0.421
RT_018437.1	FAM192A family with sequence similarity 192, member A	TVHAAYR	NP_079222.1,	XP_00525	8802	TAGtttctccgtccctacacagggagctcctccccaagggtagatcggaccgttcatgctgcctataggcattatgtccc<br>tcaaaaaaaaactcctttgcctgcatcctgtgtacaacatgacatttttaaccaatccaatctaaaaatgtgccagaatc<br>cacctgtggcccgaatcgtgtttggttcctctttctactccactgcagatgaccaaacctgtcccgctgccactttcctc<br>actgatattgggaggagggcaaggcccagccgaagttccactaaaaatgccccaggagaatag	0.236	0.104
RT_011736.1	FAM213B family with sequence similarity 213, member B	AEMGLIPPGGVGGPWRLSGDGPA(	NP_001182667	XP_01153	21033	TGAcagagaggtgtgagggaggcgaaggccctggcctccgaggatctgggtggcgtctgctgccctaggtgtgctggaag<br>tccacttggaagaactgttccggaggcgctgggtcgggatgccgaacctctcctgatccgccggcagcaacgagccatta<br>aaactgcagttcctgaccacgcactgcttcgcaggctccgagccctgcatcctccacagcccccgccttgctcactgttg<br>ggccctcagggcgggcagggtggctgtgagtcccaggtgtcatcttcctgctctgcgactttctctggagaccttgggcc<br>tttggcctgtggggcactggggatggtaacctcactgccccgtcactcccttcaaaggcgacagacccaagcccacgtca<br>ggagaggagcgtggggtcagctccagcagggtcggggtcagtgcctgtgtctggtgggggccactcaggagagttgggtt<br>ttcagggcagagatgggcttgattccacctggtggggtgggtggaccctggagactctcaggtgatgggccagcctgccc<br>caaggctctttactga	0.316	0.33
RT_012764.1	FANCE Fanconi anemia complementation group E	GPPSWCSITSFLK	NP_068741.1,	XP_00524	4680	TGAccatccaccaagggaccaccctcttggtgctccatcaccagcttcctgaagggcatttcttcttcaccaccttgcttga	0.371	0.658
RT_025932.1	FBL fibrillarin	SSALSGLR	NP_001427.2,	XP_00525	120	TGAagttcagcgctgtcaggattgcgagagatgtgtgttgatactgttgcacgtgtgtttttctattaa	-0.004	0.153
RT_026099.1	FBXO10 F-box protein 10	GHRPASGPSLDGCSEELRWK	NP_036298.2,	XP_01151	4883	TGAggacacagacctgcttcagggccaagcctggatgggtgctcagaggagctaaggtggaaatactgcctgaaaagact<br>ccgcccctcccccctcccccctcgaacaaatgggtcccgggcctccaaaacacggtacctccagaacacttcagctccacag<br>cccatagcaagaagaaagtgggcaatgttttttcagggagaagggatagaagctggtgggtttccagtcactcgaagtctataa	0.195	0.345
RT_024737.1	FBXO21 F-box protein 21	LHGALGTCCTRK	NP_055817.1, N		2900	TAAagtctagagaggacattgcacctttgctgctgctgctatcttccaagagaacgggactccggaagaagacgtctcca<br>cggagccctcgggacctgctgcaccaggaaagccactccaccagtagtgctggttgccctcctactaa	0.081	-0.025
RT_011219.1	FGFRL1 fibroblast growth factor receptor-like 1	GPMARRNGQHPR	NP_001004356	XP_01151	7158	TAGacggcaccgtatctgcagtgggcacggggggccggccagacaggcagactgggaggatggaggacggagctgcagacgaaggcaggggacccatggcgaggaggaatggccagcaccccaggcagtctgtgtgtgaggcatag	-0.033	-0.155
RT_007474.1	FMNL2 formin-like 2	TCVLLMVPK		XP_01150	2309	TGAggagaagcgtcaggcggcgctttgatgatcagaacttgcgttctgttaatggtgccgaaataa	0.136	0.345
RT_020024.1	FRK fyn-related Src family tyrosine kinase	TLEKNIK	NP_002022.1	XP_01153	386	TGAacactggagaagaatatcaaataa	0.046	0.384
RT_019443.1	FRMD4B FERM domain containing 4B	SHTSVPCKLYLSSERMFVLET	NP_055938.1,	XP_00671	6767	TGAactgcagttggacctcctgaccaagcactgcgttctcacactagtgtgccttgcaaattgtatttgtcttccgagag<br>gatgttcgttttagaaacctaa	0.206	0.1
RT_026450.1	GDF9 growth differentiation factor 9	QMVLLKP	NP_001275753	XP_01154	7924	TAAcaaatggtcctcttaaaaccttga	0.24	0.468
RT_014494.1	GGT5 gamma-glutamyltransferase 5	TVARTRPR	NP_001289393	XP_01152	6149	TAAgactgtaggaggtgcagaggggactccaagaccgtggccagaaccagacccagaggcccttcttcctga	-0.034	-0.123
RT_003442.1	GLB1L2 galactosidase beta 1-like 2	SISLSSNLR	NP_612351.2,	XP_00671	5181	TGAgcggtggcacccccctcctgctggtgccagtgggagactgccgcctcctcttgacctgaagcctggtggctgctgccc<br>cacccctcactgcaaaagcatctccttaagtagcaacctcagggactgggggctacagtctgcccctgtctcagctcaaa<br>accctaagcctgcagggaaaggtgggatggctctgggcctggctttgttgatgatggctttcctacagccctgctcttgt<br>gccgaggctgtcgggctgtctctagggtgggagcagctaatcagatcgcccagcctttggccctcagaaaaagtgctgaa<br>acgtgcccttgcactggacgtcacagccctgcgagcatctgctggactcaggcgtgctctttgctggttcctgggaggct<br>tggccacatccctcatggccccattttatccccgaaatcctgggtgtgtcaccagtgtagagggtggggaagggggtgtct<br>cacctgagctga	0.044	-0.025
RT_007096.1	GLYATL3 glycine-N-acyltransferase-like 3	PSKKLQWFYYFP	NP_001010904	XP_01151	11307	TAGcccagtaaaaaactgcagtggttttattactttccctga	0.129	0.21
RT_024798.1	GPR17 G protein-coupled receptor 17	AGGAVQAER	NP_001154887		1128	TGAgcggggggcgccgtccaggccgagcgcagactgtttaggactcagcagacccagcaagaggcatctgccctttcccc<br>agccacctccccagcaagcaacctgaaatctcagcagatgcccaccatttctctagatcgcctagtctcaacccataaaa<br>aggaagaactgacaaaggggatcatcggccaccccctctgcaggggcttgtga	0.133	-0.025
RT_023626.1	GRID2 glutamate receptor, ionotropic, delta 2	SLHCFFFR	NP_001501.2, N	XP_01153	5438	TGAgcatcaaacaaatcttcactgtttctttttaggactccctttgcaaggagcaactgtaatattgtgggactaac<br>atggatgtaa	0.19	0.292
RT_000656.1	GRIK3 glutamate receptor, ionotropic, kainate 3	AQLGWGPQAWGLGRGK	NP_000822.2,	XP_01153	865	TAGgcacagctggggtggggacctcaggcctgggggctgggcagaggaaagcaaaggagattggaaggaacgtcccctgt<br>ccccgcactgggcttggggaccagagctgccacctgcctgttgggccaggagcctcctgcccttacctgccaggaagcca<br>gcaggctctcaggccagctgcttgggcttcatcctcctcagatcttctgtgggtttctaaagctgccagccgagatagcc<br>aaggccaaaggaagcacatgcctctctcaggccaaactcacctgcccctcaactctcctccagagtcagaagtttctgcc<br>gcagccctgcagagggcacagaaaatggaagacagctcttatattgccatttcttccacaagagcccaggcctcctacag<br>cttga	0.122	0.07
RT_006083.1	GRK6 G protein-coupled receptor kinase 6	APFTVLHSDLPHCPLK	NP_002073.2,	XP_00671	3763	TAGcagctactccgagcgccgtttacagttttgcacagtgatcttccccattgtccactcaagtcgtggcctggggaaca<br>cagacggagctgtccccagtgtcctccgtccctcagcccctggcctggctga	0.05	0.107
RT_002849.1	H2AFV H2A histone family, member V	RDALTNPLPPR	NP_958844.1, N		14036	TAGagggatgctttaaccaaccctcttcctccccgtcattgtactgtaactgggacagaagaaataatggggatatgtgg<br>aatttttaacaacagttaa	0.214	-0.102
RT_012910.1	HAGH hydroxyacylglutathione hydrolase	GRPAPSADLGIR	NP_005317.2,	NP_001035	2154	TGAggccgccctgcaccttcagcggatttggggattaggctcttttag	0.028	0.119
RT_012805.1	HAUS2 HAUS augmin like complex subunit 2	SSTLFLLNYV	NP_060567.1, N	XP_00672	7221	TAGtcatcaactttatttttgcttaattatgtgtag	0.019	0.106
RT_006180.1	HEATR9 HEAT repeat containing 9	QSILIFR	NP_689994.2	XP_00527	5817	TAAgtcaattctttgggcctactccccgactccctccctgaagatgcttctcaggctcccatgagaaaacaatctatact<br>tatcttccgtgaataa	0.115	0.17
RT_023343.1	HOXB8 homeobox B8	ASAGTARAAAAR	NP_076921.1	XP_00525	1334	TAGgcttcagctgggactgccagggccgcggccgcccgcacgtccgcgggtcccggccgcgccgccgccgcgcgcccctg<br>cccgagagagctctggccccgctag	-0.2	-0.215
RT_007294.1	HPN hepsin	STGPRTPSLQGPLFHSGGPTQPRDH	NP_892028.1, N	XP_00672	1019	TGAccggtggcttctcgctgcgcagcctccagggcccgaggtgatcccggtggtgggatccacgctgggcctaggatggg<br>acgtttttcttcttgggcccggtccacaggtccaaggacaccctccctccagggtcctcttctccacagtggcgggccca<br>ctcagccccgagaccacccaacctcaccctcctgac	0.314	0.341
RT_010197.1	HSPA12B heat shock 70kD protein 12B	VGAGETIVLQSAPFHALQPR	NP_443202.3, N	XP_00526	3550	TGAgggcgcgccggcgcggtgccagcgccgtctgcccggccccgccctctttcggttcaggggcctgcggagcgggttgg<br>ggcgggggaaacgatagttctgcagtctgcgcctttccacgccctccagccccgggggagataa	0.142	0.028
RT_012273.1	IDS iduronate 2-sulfatase	VLPTMDGK	NP_001160022.1, NP_000		3218	TGAgttttgccaaccatggatggcaaatgtgatgtgctcccttccagctggtga	0.075	0.289
RT_004577.1	IFFO1 intermediate filament family orphan 1	GRGWGCTEGNISCSSAPGR	NP_542768.2, N	XP_00671	3036	TAGagacctgcctccccttgcacccgaggccctgcagcagggagctcagcgaggcagagggtggggctgcacagaggg<br>gaacatcagctgcagctctgcaccaggccggtccctggggactggggcgctcctccctcaggctttctccctcagtcttg<br>gcttctccagggctctggggtgtctggagctaggcttggccctaccattctggggccatttccaccacagttggggctct<br>cctgccttcacgcgtgggtgtctgctacttccccatctttaa	-0.066	0.215
RT_016397.1	IGHMBP2 immunoglobulin mu binding protein 2	WQTMLRLHQGHRGAEGPMGEER	NP_002171.2,	XP_00527	4725	TGAccggccgcatccttgcacgccccgcggagctctctccatggtagcccagggcgctggcagaccatgctccgcctcca<br>ccagggccacagaggagcggaggggcctatgggggaggagcggagggccctgttggggaaggttgggtttttggaccca<br>gggataagcttttccgatgtcacaatgtggaggaaagcacctgggggacacagtgctcgtgcaggtggggcttgggaaa<br>tgcacgtcccttccccttactccccgccaaaacccacatcccagcctctggatcctggggaaggttccagtccctggaga<br>atacccagggcctcaaacttga	0.278	0.341
RT_011586.1	IKZF1 IKAROS family zinc finger 1 (Ikaros)	KSTRTAAFSLPPAA	NP_001278768	XP_01151	10190	TAAagccctcccgcgcccccaccccagaccccgagccacccccaggaaaagcacaaggactgccgccttctcgctcccgcc<br>agcagcatag	-0.107	0.033
RT_023045.1	IL17RE interleukin 17 receptor E	IPCLTGRKSQPSPR	NP_705616.2, N	XP_00671	1412	TGAgcagagctccaccgcagtcccgggtgtctgcggccgcaacgcaacggacactggctggaaccccggaatgagccttc<br>gaccctgaaatccttggggtgcctcgaggacgactggccgaaaagccgcattccctgcctcacaggccggaagtcccagc<br>ccagtccccgcgcgcgtccctcttcctcctcatactttcccttgactgagagctcctctaacccctgttctgatggggga<br>gggcggtcttcccacttcctctccagaactccagaaagagcagtgtgcttatgcttcagtccaggctggagaggttgggg<br>ccggggtagggaggcaggagccatgtcagttctgaaggagggtgaggcggtgggggattgcagggggcggctgagagaaa<br>acctccttggggccagggattccctttcccactctga	0.154	0.292
RT_008965.1	IL33 interleukin 33	RELGLPDPLK	NP_254274.1, N	XP_00525	770	not found		
RT_025506.1	IL5RA interleukin 5 receptor, alpha	LTSSCIYFWLCPFGCLSCQL	NP_783853.1, N	XP_01153	4995	TGActgtcactttggcatcctctgatgaactcacacatgcctcagtgcctcagtgaaaagaacagggatgctggctcttg<br>gctaagaggtgttcagaatttaggcaacactcaatttacctgcgaagcaatacacccagacacaccagtcttgtatctct<br>taaaagtatggatgcttcatccaaatcgcctcacctacagcagggaagttgactcatccaagcattttgccatgtttttt<br>ctccccatgccgtacagggtagcacctcctcacctgccaatctttgcaatttgcttgactcacctcagacttttcattca<br>caacagacagcttttaaggctaacgtccagctgtatttacttctggctgtgcccgtttggctgtttaagctgccaattgt<br>ag	0.208	0.623
RT_020939.1	JMJD8 jumonji domain containing 8	GGQEHKLCIGAGGFCPGLPWTR	NP_001005920	XP_00525	7736	TAGccaaaacagctggcaggactgccggtcacacaccagcacgtcccacctcgtgctcacggatttattacacagatag<br>tggcggcaatggcctcagcccagcccacccctcacctgcttttccagcccacaaagggggacgatcacggcccagcaaaag<br>cgatgctgagaggggaaacagtccagagtccaacagcagaacttgggggaagcggtcggggtggccaggaacataaacta<br>tgtatagggcgggggcttctgcccagggctcccctggaccaggacgccaggtagggcagggaacctcagtagtcctcc<br>acccagcattctcagagatgaatgcgtcaataaccttccttcatagccaagttggggatga	0.182	0.435
RT_010302.1	JUND jun D proto-oncogene	ARPPGIPPPRGCPGLDK	NP_005345.3, N		752	TGAgtccgcgcgcggggcgcatgcgcggccaccctccccaaggggcgggctcgcggggggtgtcgtgggcgccccggacttggagagggtgcggccctggggaccccccctccccgagtgtgcccaggaactcagagagggcgcggcccccggggattc<br>cccccccccgagggtgcccaggactcgacaagctggacccctgctcccgggggggcgagcgcatgacccccgccctc<br>gcgctgcctctttcccccgcgcggccgccccgtgttgcacaaacccgcgcgtctcggctgccccctttgtacaccgcgccgcggaaggggggctccgagggggcgcagcctcaaaccctgcctttcctttacttttactttttttttttttctttggaagagagagaagaacagagtgttcgattcgtgccctatttatgtttc	0.187	0.348
RT_009378.1	KCNG2 potassium channel, voltage gated modifier subfamily G, mem	RPHGDPLPPPAAASGPPR		XP_01152	16826	TGAcgcctgcgcgcccacacggagaccccctgcccccctccagctgcagcgtcgggaccccgaggtgcgccaaggggtg<br>gggggcgtctggcctgggggagcggctcctgccggccgcgtcctcggccctcgtgcgtgagcagccccagaacttggcggggccctgcctgactccccgtggcagcgctgggcaaagtcactggcctttgtcctcctgccccaccccttttcttggatttttaattttctacgcctag	0.195	0.485
RT_000486.1	KCNU1 potassium channel, subfamily U, member 1	KILPTFTLLNSLVDWK		XP_01154	14563	TGAaaaatccttccaacattcactttattgaacagcttggtggactggaagggtccctccaagaaacaaatctgcatctc<br>agcactgcctttt	0.108	0.373
RT_003017.1	KIAA0513	RTEAMCHSPGPSARPSPHPMTCMI	NP_001273495	XP_00525	10909	TAGgccccagaggtcgcactccgcaggaggactgaggccatgtgccattctcccgggcccagcgcccggccgtcacccca<br>cccgatgacctgcatgaagccagcagcacccagagccactcctgctgccctagaactagcggttagaagaatccgctgtt<br>cctccctcatctcctctgcctgtgtctgcgacccccatccatgtgccaaagtgtcccttgggtcacacagctaa	-0.046	-0.156
RT_021510.1	KIAA1191	SPLSRDSGK	NP_001274264	XP_00526	8368	TAGagccctcttccaggattctggtaaaggtggtttcttgcatcccactcccctttaccttggctttga	0.014	-0.011
RT_003800.1	KIAA1683	DPGSLQWGLRGR	NP_079525.1, N	XP_00526	3408	TAGgaccctggctccctgcagtggggacttcgtgggaggcactcatggctctctgggtctaatgaataa	-0.065	-0.167
RT_010079.1	KMT2D lysine (K)-specific methyltransferase 2D	RGSSQSQDPGLGLPAD	NP_003473.3,	XP_00526	6602	TAAgaagctttgaggctaccaggcaggggagtccccctacccacaacctcttccctgaaagggatgaggggaagagagg<br>tagcagccagagccaggacccaggttggggctgccggctgacccggagccctggagcaggaggctggggcagagggcc<br>ctaggccaagcccacccttgggcaccagggacaatccctcttccccaccaccggccctcaggctggcatctctgcccccagc<br>tccaggagggggccagacagaagcagccattgggcatctcaggtttgagggggatatgggccgggaactacccagaagcat<br>ctgggaggcagcagggtgggggaagaggatgtgtggccgggcctcacagccctgctgctcccactga	0.021	0.103
RT_005240.1	LAMTOR2 late endosomal/lysosomal adaptor, MAPK and MTOR acti	NDHLEGR	NP_054736.1,	NP_00113{	2906	TAAcggcattggtggaagctggggtcagaaaagagaaatgaccatttggaggggcggggcctcctagaagaaccttcttag	0.198	0.216
RT_017114.1	LIPG lipase, endothelial	TSCYPSPWRKVTAEDPPNGR	NP_006024.1,	XP_00525	5528	TGAgggtgcccgggcaagtcttgccagcaaggcagcaagacttcctgctatccaagcccatggaggaaagttactgctga<br>ggacccacccaatggaaggattcttctcagccttgaccctggagcactgggaacaactggtctcctgtga	0.063	0.028
RT_023299.1	LMTK2 lemur tyrosine kinase 2	VAANAHARVRGCSPGAAPLRPQPE	NP_055731.2	XP_01151	13730	TAGgtggctgccaacgcgcacgctcgggtccgaggctgctcccctggagcggcgcccctgcgccctcagcccgagcagcg<br>acatccactcgccatttgctga	0.16	0.035
RT_001154.1	LOC102723730 adhesive plaque matrix protein-like	SPWPTGLR		XP_01150	17842	TAAttccctatttctcagcctctgctggtgaagtttctgcctcttacttcagaagtccaagtggtgaagagcccttggcccacaggtctcagggtggtaggaacgaaaaggccgctctga	-0.025	0.207
						TGActggtccacgcacggccaaagcatgaggaagctcatcgtgggattcatttttcttacattttggacttacacagtaa		

Biology Open • Supplementary information

RT_013953.1	LOC105373926 putative HERV-K_Xq28 provirus ancestral Pol protein	GIHFSYILDLHSKGFN		XP_01151	8461	gggcttcaactga	0.399	0.623
						TGAcggctcaccataaaagccaaaatggggacctgtcaaaaatgttttagccaaactatggcttggctcaggaagatgcc		
RT_000939.1	LOC643355 uncharacterized LOC643355	KMPRPK	NP_001257874	XP_00527	8225	aagaccaaaggctgctgaactttaa	0.209	0.394
						TGAacaacccaggccatggggtggaaacggcttgggcttcaagcctctgaaaccaaccctctgcagcagctccatccatt		
RT_024143.1	LRIT2 leucine-rich repeat, immunoglobulin-like and transmembrane	TTQAMGWK	NP_001017924		801	tatccagcaagtatatattgagcacccactgtgttccaagcactga	0.205	0.384
						TGAggcggcggggccgggcgggcgaggggcgtggagccccccacccaggtcccagcccgggcgcagcctgaccgggacccctccctcccacagcccagcccaccttctgggaccacgcagggaattggggagaggtggcttccagccccatctggggctc		
RT_007580.1	LRRC4B leucine rich repeat containing 4B	GGGAGRARGVEPPTQVPAR	NP_001073926	XP_00672	1548	ggacccccagtaaggacagggtggggctccaggagcggagtctctgggatcctcgcctcacccccgccaatccccggtga	0.16	0.119
						TAAattccagctctgatcaggcaactggttggtgactggagagcattgtttcataggcttttctctgtcctatctgggag		
						cgctgcttcttcccctgccttccgagagacgaagaccgtggcgagcttggcgcttaggggctcccgtgccatggctcacc		
						ccagggagccccagcagccaccaggtgcctctgtctgcagaccccctggcccgggctggcgccgacgctcagaacctgcag		
RT_026848.1	LRRFIP1 leucine rich repeat (in FLII) interacting protein 1	TVASLALR	NP_001131022	XP_01151	10123	gtacttcataagcacacaggggcctcgagggagctctgtgtctga	-0.019	0.204
RT_006265.1	MAOB monoamine oxidase B	RERVSVITLSSYCIWDMSLGK	NP_000889.3,	XP_01154	6600	TAAagagagagggtgtctgtaatcacactctcttcttactgtatttgggatatgagtttggggaaagagttgcagtaa	0.26	0.259
						TGAgagctgaatgagggcatcataggccagacaggcccaaggatggatgaatggagaggacaaaggcagcttctgacacaccagccccaggacctggggcgactggaggaagccaggcgagtggggcccaggactggttccagtgagagaaaccaaccacaggcacccaagcactaccagac		
RT_023252.1	MAP3K6 mitogen-activated protein kinase kinase kinase 6	ELNEGIIGQTGPR	NP_001284538		5739	gtgggaggggtatcctgggaagagcccccctaggtctttttaaaaaatttttttgttggccgggcatggtggctcatgcctgtaatcccagcactttgggaggccaaggtgggcagatcacgaggtcaggagatcaagactatcctggctaa	0.059	-0.036
RT_013519.1	MAP7D1 MAP7 domain containing 1	EGLPWIR	NP_060537.3, N	XP_00671	2843	TAAgagggtttgccttggatccgggcacagttgtga	-0.005	-0.214
						TAAgtagcaacgccactccttctcagtcttcatcgatcaatgacatttcatccatgtccactgagcagacgctggcctca		
						gacacagacagcagtcttgatgcctcgacgggacccccttgaaggctgtcgatgataggttagaaatagcaaacctgtcag		
RT_011694.1	MAPK9 mitogen-activated protein kinase 9	VATPLLLSLHR	NP_620707.1, N		3409	cattgaaggaactctcacctccgtgggcctga	0.151	0.396
RT_022686.1	MARK2 MAP/microtubule affinity-regulating kinase 2	QAARSGGGGGGPAGR	NP_001034558	XP_01154	659	TAAcaggctgccaggagcgggggcggcgggggcgggccagctggacgggctgccggccgctgcgccgccccacctgggcgagactgcagcgatggattggtgtgtctcccctgctggcacttctcccctccctggcccttctcagttttctcttacatgtttgtggggggtgggagattgttctccagcacc	0.181	0.152
RT_022228.1	MCAT malonyl-CoA-acyl carrier protein transacylase	LQGAQMR	NP_775738.3	XP_01152	10785	TGActgcagggggctcaaatgcgatga	0.257	0.623
RT_027052.1	MED1 mediator complex subunit 1	EPYFLKETGPEEK	NP_004765.2	XP_00525	10723	TAGgaaccttatttcctaaaagaaacagggccagaggaaaaaaaactattgataagtttatag	-0.112	0.058
RT_014431.1	MED12L mediator complex subunit 12-like	RHDVLCLH	NP_443728.3	XP_00671	5306	TGAatctgcaagaggagaagacatgacgttttatgtttgcactga	0.257	0.44
RT_022201.1	MEI1 meiotic double-stranded break formation protein 1	SSGLEGPEVER	NP_689726.3,	XP_01152	9595	TGAtcctcaggacttgaaggcccagaagtggagagagaatga	-0.087	0.103
						TAGcagctacctgctcagccctgcaccatgctgctggggaagacagtggccccagccccgtcaggcagggccagggacag		
RT_017809.1	MGAT5 mannosyl (alpha-1,6-)-glycoprotein beta-1,6-N-acetyl-glucos	SHAGTLARA	NP_002401.1	XP_00526	2131	aagtcatgcagggactctggcaagagcctga	0.125	0.107
RT_013659.1	MID1IP1 MID1 interacting protein 1	KMVAGDGR	NP_067065.1, NP_001092		1487	TGAggcgtggcgcccgtggctgcccagcaccttcttcgacccatctcaccctctctcattcctcaaagcttttttttttttcctggctggggggcgggaagggcagactgcaaactgggggctgcgtacgtgcaggaggcgcggtggggctgcgtggaggagggggccacgtgtgagagagaagaaaa	0.154	0.119
RT_001122.1	MIS12 MIS12 kinetochore complex component	LLSSQKEEPVK	NP_001245146	XP_00525	2641	TAAttgctcagtagtcaaaaggaggagcctgtcaaaaagtag	0.066	0.116
						TAGcgaatcctccctgcactgcattcgcacaaactgcttcctttcttgattcgtagatttaataacttacctgaaggggt		
RT_019879.1	MITF microphthalmia-associated transcription factor	RILPALHSHK	NP_937802.1, N	XP_01153	3922	tttcttgataattttcctttaa	0.278	0.488
RT_021866.1	MPV17L MPV17 mitochondrial membrane protein-like	EVKDSLK	NP_001121895	XP_01152	10879	TGAgaagtcaaggactctcttaaagggaccacattttttacctaa	0.219	0.281
RT_021358.1	MSL3 male-specific lethal 3 homolog (Drosophila)	NVVGSVR; ATALSSLALWVPGE	NP_523353.2, N	XP_00527	1444	TAAaatgttgttggttctgtaagagcaactgctctgtctagtttggcgctctgggttccaggtgaataa	-0.003	-0.089
RT_010471.1	MTR methionine synthase	FAVLGTMVMK	NP_000245.2, N	XP_00527	10332	not found		
RT_020347.1	MTSS1 metastasis suppressor 1	VHKKCAGGE	NP_055566.3, N	XP_00671	4627	TAGgttcacaagaaatgcgccggtggggaatga	0.134	0.067
						TGAgagccacagctgacaaagtctgcatgtccgaggacggcccctgcactggagctgggcgccagagctgcagagctagt		
						gttcggccctcagagaaggatccagaatcaaaagctcaagagtgacgtgaggtgggcaccggccccaagtgcagagtcaa		
						ggcagggagaggccggctggagccaggccccctcgcacgcagcccccaaatcatggacgcacctgtggggagcaccacat		
RT_025500.1	MYO9B myosin IXB	LKSDVRWAPAPSAESR	NP_004136.2	XP_01152	1548	ctccacctgcggcctcacatctccccactccccttttttgtacgtttaa	0.079	-0.036
RT_006029.1	NCAM2 neural cell adhesion molecule 2	QQYYRGLNNTTK	NP_004531.2,	XP_01152	14703	TAAcaacaatattacaggggcttgaacaacactacgaagagtatttggattgcgtga	0.29	0.468
						TGAgtggggagcaccgaggcaggggtgggggaatgtgtactgaggagccgtgtgtctgctcctggctggcccggcctaat		
						aaagcagtgttgccatctcatcttcccccctaaaaaccccttttatgtacacctgcgcagagaagagggctgcctggcctccctgggccgctggtacagatcacacacacacacagattaaacgcagcccgtttaatgatggggcccagactgcagtggctcaagacaggacactgcggaaagccgccatgt		
RT_009713.1	NCAPH2 non-SMC condensin II complex, subunit H2	LNAARLMMGPR	NP_001171940		1370	taatccgtga	0.233	0.257
						TGAggggctgcgccacgccacggccccgctggagctggggaccacagactggaccggctctcttcatgcccagcccccgg		
RT_014759.1	NCKAP5L NCK-associated protein 5-like	LSSCPAPGDGDPSLK	NP_001032895	XP_00671	3669	agacggggacccctccctgaagggaccaaggaggcaggtggataagaaggtgaaaagggggtccctggcacaccccaccacccactgcttcggcggatga	0.164	0.028
						TGActcagaacagggcggggggggggcggtgtcaggtcccagcgagccacaggaacggccctgcaggagcagggcggctgccgactcccccaaccaaggaaggagcccctgagtccgcctgcgcctccatccatctgtccgtccagagccggcatcctt		
						gcctgtctaaagccttaactaagactcccgccccgggctggccctgtgcagaccttactcaggggatgtttacctggtgctcgggaagggaggggaaggggccggggagggggcacggcaggcgtgtggcagccacacgcaggcggccagggcggccagg		
RT_015970.1	NCOR2 nuclear receptor corepressor 2	HPCLSKALTK	NP_001070729		13497	gacccaaagcaggatgaccacgcacctccacgccactgcctcccccgaatgcatttggaaccaaagtctaaactga	0.265	0.714
						TAGgcatttcaaagcatcacacttgtctgcaggacttacagatcctgcagtcaatgtttcggtttagactctccactgtt		
						acctaagttctcaagctgcctatggtttttctgtgtcaatgtgatttatggtagtaccatcctttctcctttgggtttta		
						aaataagttgcagaacagacactttaaaagcttctgcaatattatttctgtgcctagagtctttctccattataaacatg		
RT_006054.1	NEB nebulin	AFQSITLVCR	NP_004534.2, N	XP_01150	727	ttttaa	0.092	0.07
						TAAgatttgagtccattgcaaaaggttaagccatatgacaatttcaaaatgcatgtgattggcagcttcaaaacagaacg		
						ggttctcccatgggggctccagacattgtattttactttgtgcaatatgaggggactgcatgcaagctcagggtgctccc		
						tcctcagtctttgggggattcaaatgcatgatattgtatgtacctgggaaatttgccgatttcctaagctgttggaaggg		
RT_001896.1	NEFM neurofilament, medium polypeptide	VLPWGLQTLYFTLCNMR	NP_005373.2, N		19012	ggtcacttaagggggggatgtcttgagatgtattatgcaaagtaccaactga	-0.018	-0.182
						TGAccagccccacccccctccccagcagccagagaagcagtggcccggggcagttccgagggcagtgcctgcagtcaagtg		
RT_019335.1	NFASC neurofascin	QWPGAVPRAVPAVK	NP_001005389	XP_00524	284	gccgggtcaggcgtggtga	0.407	0.658
RT_016847.1	NFIC nuclear factor I/C (CCAAT-binding transcription factor)	LPKLFISSPPAAR	NP_995315.1, NP_001231		2713	TAGcaaaggtcttcttccctcgccccttctccatcgtcccaggaatcccagggggcagcacagccggcccccggcccacgttttcggtggaaaattagagtgaacaagaacacccctgccgactcccagcccggccaaaaagacaaaacacatagacgcacacactcaggaggaaaagaaaaaaca	0.104	0.424
RT_008486.1	NLRC5 NLR family, CARD domain containing 5	WPPQDLWNPAK	NP_115582.4,	XP_00672	274	TGAtggccccctcaagacctttggaatccagccaagtga	0.086	0.065
RT_015312.1	NPL N-acetylneuraminate pyruvate lyase (dihydrodipicolinate synthas	EDCLSRRTLNY	NP_001186985.1, NP_001		6156	TGAgaagactgtctctcccgcaggaccctgaattattga	0.22	0.281
						TAGggcgcctgccctctgctggctaggaccgggcccagcccagcccttcctgaagccagaagcgttgctgagtgtgttcc		
RT_016124.1	NPLOC4 NPL4 homolog, ubiquitin recognition factor	GACPLLARTGPSPALPEAR	NP_060391.2,	XP_01152	846	ctgtaactgccccatagtgggcagccctggaggaacaaggggctggctgtcctgggctccctga	0.029	-0.103
						TAGagaggtgggggaggtgacagggagctaggcagccgtgctccctccagtgcggacttgtctccctctgagggaggcaa		
						gaggctggcgatcagggatcttgttgcattgggagcaggggcggctctcctggtccccaggagagatgctttgaggagca		
RT_009589.1	NUDT18 nudix (nucleoside diphosphate linked moiety X)-type motif 1	APSSADLSPSEGGKR	NP_079091.3	XP_01154	600	ttcctctagattgcacaagggacagtgcctttaaccaagcgaggagtccaaagctcaggacctga	0.128	0.215
RT_003245.1	NXN nucleoredoxin	GCMAFAKSLAFGEGSARVSWAE	NP_071908.2, N	XP_00525	391	TAGcgtggctccggcctcctgagacgttatttaaaactcagccttctcctcctccccctccttccttccgcccttggacttacccagcgtgccccgaatcccaccacccaagtgtccagcctctctgtggtgccttgtttctgcagtaaactcctcagccagcaccctggggtgtggaatcagcagcggcagagtc	0.25	0.203
						TAAtgcattacagctgtgagaaagagcactgtggctttggcagctgttctacaggacagtgaggctatagccccttcaca		
RT_016280.1	OLFML1 olfactomedin-like 1	CITAVR	NP_940876.2,	XP_00525	2996	atatagtatccctctaatcacacacaggaagagtgtgtag	-0.12	-0.022
						TGAgtggagacctgtgctccccggagaggggcagcagtgcgggaggggctttgcacagcagctcctgcaactgacccagt		
						ccgcaaatatttattgggggccagcccagggctgggactgggcatgaggtggtcaccaggattgagcttcctcagcaccc		
RT_014428.1	OLFML2A olfactomedin-like 2A	VETCAPRRGAAVR	NP_872293.2, N	XP_00671	974	agtgggtaatacttgcttccacttgcagagcaccgtgccaagcacttcccacacacttacccgtttga	0.315	0.257
						TAAattaaaatcgtactcataatcagctctgcatacatctgaagaacaaaaacatcaacgtcttttgtccagcctctttt		
RT_010142.1	OPA1 optic atrophy 1 (autosomal dominant)	IVLIISSAYI	NP_570850.2, N		1229	tcttctgctgttccacctttctaa	0.054	0.204
						TGAagggggatggcatctggtagggagggaggtatagttgggggacaggggtctgggtttggctccaggtgggaacaagg		
RT_015055.1	PAQR7 progestin and adipoQ receptor family member VII	WEQGLVK	NP_848509.1	XP_01153	3864	cctggtaaagttgtttgtgtctggcccacagtga	0.138	0.12
RT_005430.1	PARP10 poly(ADP-ribose) polymerase family member 10	APEGGVGDGGQQRPR	NP_116178.2,	XP_01151	3568	TAAccgaagggggccaccctctggcctcctgcttcccaggctcccagctccgcacaggctgatgctccccgcccccaactgtggccgcctgagctgtccccggggacgcccctgcctccctctgcgggctccagaaggcggtgtgggggatggcggtcagcagcggccgagggggccgggctaggtccc	0.177	0.163
						TAGttaccgcggggaccacatcaagcttcaggccgacagcttcggcctccacatcgtccccgtctgacccacccccggag		
						ggagggaggaccgacgcgacgcgatgcctcccggccaccgccccagcctcaccccatcccacgacccccgcaacccttca		
						catcacccccctcgaaggtcggacaggacgggtggagccgtgggcgggaccctcaggcccgggcccgccgcccccagccc		
RT_017560.1	PAX2 paired box 2	VGQDGWSRGR	NP_001291498		7788	cgcctgccgcccctccccgcctgcctggactgcgcggcgccgtga	0.08	0.388
RT_014634.1	PCBP2 poly(rC) binding protein 2	NNADSSIIPFCCSPPPMIHLCSF	NP_005007.2, NP_001122		4813	TAGaacaatgcagattcatccataatccctttctgctgttcaccaccacccatgatccatctgtgtagtttctga	-0.054	-0.075
						TAGatagccggctttgccatccgggcatgtggccacactgcccaccaccgacgatgtgggtatggaacccccctctggata		
						cagaaccccttcttttccaaataaaaaaaaaaatcatccagggcttggtgctttgtatcctagcttgtattcctttgaaaagcaacaggaggaaattggccaatatccagcactggggactgaggctttcctgtaaatgcaaggggttgggtggggagtgg		
RT_026547.1	PEBP4 phosphatidylethanolamine-binding protein 4	IAGFAIR	NP_659399.2	XP_01154	6085	ggggaatag	0.365	0.444
						TAAaagattttctgtagtgtttttgaaaagtttgcagcttatgtaatagcagataaaatttctaattgtaaaatgttaaa		
RT_014747.1	PHF14 PHD finger protein 14	FAAYVIADKISNCKMLNCK		XP_01151	10015	ttgtaaaatctaa	-0.055	-0.122
						TGAatacaaaataaaacaaataatttgtatttatggagtggaaaacagcttatttcaaggaaaaatatgactgccttatt		
RT_013650.1	PIAS2 protein inhibitor of activated STAT, 2	NMTALLLLVPYCQVR	NP_775298.1	XP_00672	3105	attgctagtgccatattgtcaggtgcgaatggtgctcaaaagttgtatcattaaaatttga	0.22	0.666
RT_010003.1	PICK1 protein interacting with PRKCA 1	GLGGGRSPSPAPCGRSHCPR	NP_036539.1, N	XP_01152	2783	TGAgtgccccgcggctgtggtgccgggggcagggtgcgtgggaggacggagcctgggagcggggcggggccgccgcgcaagggggcgacgcataaaggcctgctggcttggggcgcctgcctccctgctcctctgtcctcgcacagcgaacctgggctcctgcccaggacaggcaccagggtcatg	0.28	0.257
RT_023912.1	PLAC1 placenta specific 1	SSGLGSPEDAISR	NP_068568.1	XP_01152	1924	TGAtcctcaggtttggggtctcctgaagatgctatttctagaattagtatatag	0.131	0.103
RT_006100.1	PLCB4 phospholipase C, beta 4	THSQTSEHKLHG	NP_000924.3,	XP_00526	5813	TGAagcagcaaacccacaaagcatcaaaagactcactcacaaacttctgaacacaaactccatggatga	0.127	0.211
RT_023238.1	PLCZ1 phospholipase C, zeta 1	QLMINDISLAMHRNK	NP_149114.2,	XP_00525	1169	TAAcagctaatgataaatgacatatcattagctatgcatcgcaataaaacagccaaaatgaatgtctttatgttttctttttaaacataaagaaaaataattactcataa	0.028	0.152
						TAAcatgatgaagaaactgacaggacagctctgtatttcatatttatacataaaggacttgaggagagaaaaaacacttt		
RT_009701.1	PLD5 phospholipase D family, member 5	GLEERKNTLICLFF	NP_689879.2, N	XP_01154	2529	aatatgtctcttttttttag	0.334	0.184
RT_008256.1	PMPCA peptidase (mitochondrial processing) alpha	RSEFPETAK		XP_00526	2176	TAAagagcactcaagttagtaggcgagaagggctcagtagaggactgctacattgtacgtggagtagcagtggctcggaatgccgctgtaccgttgctggctctcgggcaggagtctcagccccaggtttccggggcccccatgagtgaccttaggaggtctgaatttcctgaaactgccaagttgtatgt	0.075	0.259
						TAGaggcctctgctaaagagcccggatgcagcgtcttccgtgggactgtccccaaggctgaggctcctgccaagtcctag		
						gggcctctgtacctgccctgctggaagccctgacttccccggggccccaggctgtgttagggttctctgggcctcttctt		
RT_023570.1	PNPLA7 patatin-like phospholipase domain containing 7	SPDAASSVGLSPR	NP_689499.4, N	XP_00671	2907	tgtaccagcagccctgcatacagggccctgtgagcccccctgcagtcctgtga	0.063	-0.102
						TGAgaccaaaaagtttctcctactccagctggccctgtattccccctggaaggaagggaatcatgccttctatatacaga		
						cagattgccttcagaagagtggaagaaaatctccactatcaatgaacccagactcttgtcttcttcaagagcaagggcct		
RT_009079.1	POU2F3 POU class 2 homeobox 3	ISTINEPR	NP_001231611	XP_01154	4268	ccggagatccaaactgtgattga	-0.045	0.055
RT_020178.1	PPP1R12B protein phosphatase 1, regulatory subunit 12B	LASSISPP		XP_01150	97	TGActtgctagtagcatctcacctccttga	0.383	0.655
						TGAggatatattcaggggtccccaggaactcctcggtccagagaccaagtggtggccttggaaagcagatgtcaggcttt		
RT_020123.1	PPT2 palmitoyl-protein thioesterase 2	GYIQGSPGTPR	NP_619731.2, N		11122	ggtgtgcctgtga	0.174	0.345
						TAAaggctatggtttattgcaggggattgggaggggggcgggaaaacatggagaattaagtcagataatgctggcagcca		
						aaggggcaaaatggcctgtgacattatcctgttcagagcttggagatgtacaagggacataggagcaatttacactgaca		
RT_023177.1	PRRC2C proline-rich coiled-coil 2C	SDNAGSQRGK	NP_055987.2	XP_00524	19643	cacagctgctgtaccagtga	-0.016	-0.058
						TAGctcaggaccttggctgggcctggtcgtcatgtaggtcaggaccttggctggacctggaggccctgcccagccctgct		
RT_027064.1	PTP4A3 protein tyrosine phosphatase type IVA, member 3	HLRALVSEERGAPR	NP_116000.1, N	XP_00525	8250	ctgcccagcccagcaggggctccaggccttggctggccccacatcgccttttcctccccgacacctccgtgcacttgtgtccgaggagcgaggagcccctcgggccctgggtggcctctgggccctttctcctgtctccgccactccctctggcggcgctggccgtggctctgtctctctgaggtgggtcgggcg	0.19	0.491
						TAAgaaagcaaaaagatctgggatatgtgttggaaaactgctttcccttatgttcactgtgccataatgctgctcgcagg		
RT_009826.1	PTPN4 protein tyrosine phosphatase, non-receptor type 4	KNEELK	NP_002821.1,	XP_01150	10201	aaatggcattttacaaaaaaaaaatgaagaactcaaaaaaactttgaaaacttcagcactgttgcactttatgttttaa	0.052	0.103
						TAGctcaatgggatggggaacctgccggagtccagaggctgctgtgaccaagccccctttttgtgtgaatggcagtaactg		
RT_023418.1	PTPRT protein tyrosine phosphatase, receptor type, T	LNGMGNLPESR	NP_008981.4, N	XP_01152	10957	ggctcaggagctctga	0.357	0.491
						TGAcctggacacagacagagacagagccaggcccggccctcccgcccccgacctgaccacgccggcctagggttccagactggttggacttgttcgtctggacgacactggagtggaacactgcctcccactttcttgggacttggagggaggtggaaca		
RT_016814.1	PVRL2 poliovirus receptor-related 2 (herpesvirus entry mediator B)	HWSGTLPPTFLGLGGRWNSTLDFSF	NP_002847.1,	XP_01152	11516	gcacactggacttctcccgtctctag	0.418	0.373
						TGAcgggctttgtctgtggaaggagacagaaggaaattgacccactctcctatctccagacctttacctagcccctccag		
RT_002300.1	RASAL2 RAS protein activator like 2	ALSVEGDR	NP_733793.2, N	XP_01150	7997	gtttacagaatgttgctacttcacaatggcgatgtggtga	0.261	0.394
						TGAcagcctagtgagggctgcaagaccatcctgcccggaccacagaaggagagttggcggtcacagagggctcctctgcc		
						aggcagtgggaagccctgggtttggcctcaggagctgggggtgcagtgggggactgccctagtccttgccaggtcgccag		
						caccctggagaagcatggggcgtagccagctcggaacttgccaggccccaaaggccacgactgcctgttggggacaggag		
RT_014669.1	RASSF7 Ras association (RalGDS/AF-6) domain family (N-terminal) m	SQLGTCQAPK	NP_003466.1	XP_00525	869	atgcatggacagtgtgctcaagctgtgggcatgtgcttgcctgcgggagaggtccttcactgtgtgtacacagcaagagcatgtgtgtgccacttcccctaccccaacgtgaaaacctcaataaactgcccgaagcagcttgagtgtgtgtggaggctgcgctgggcagggtctgaggtcttgaacttgagcc	0.289	0.33
RT_013497.1	RBM14 RNA binding motif protein 14	APIMITCGRLR	NP_001185765.1, NP_001		4196	TAGccgagctctctgattaccgccgtttatcagagtcgcagctttcgttccgccgctcgccgacaaagtcctcgctggattaccgtcgcctgcccgatgcccattccgattacgcacgctattcgggctcctataatgattacctgcgggcggctcagatgcactctggctaccagcgccgcatgtagggccatc	0.152	0.119
						TAAgccgccactgcaaaaaaaaacagggtcagaaaattacttgggtgggtagacttaggaattttctacttggtaaaagc		
RT_024372.1	RFPL3 ret finger protein-like 3	AATAKKNR	NP_006595.1, N		7405	attatacagtcataggagaaagatatgggacatttctataatctatattctaatttga	0.076	-0.112

Biology Open • Supplementary information

RT_016562.1	RGMA repulsive guidance molecule family member a	SPGVSTMAPQNAR	NP_064596.2, N		2448	TAGacgcgtagatgtggagggaggcgcgggctccgtcctctcggcttccccatgtgtgggctgggaccgcccacggggtgcagatctcctggcgtgtccaccatggccccgcagaacgccagggaccgcctgctgccaagggctcaggcacggacccctccccttctagtgcacgtgacaaggttgtggtga	-0.048	-0.155
RT_020305.1	RGN regucalcin	GQVFFPAR	NP_690608.1, N	XP_00672	13379	TGAggacaggtcttctttcctgccagagggagctctgaagacaactag	0.238	0.345
RT_020623.1	RGS4 regulator of G-protein signaling 4	FSPEGRGMKCQDSMLWK	NP_001095915.1, NP_005		2129	TAAttctcacctgaaggcagagggatgaaatgccaagactctatgctctggaaaacctga	0.216	0.207
RT_015540.1	RILPL1 Rab interacting lysosomal protein-like 1	LSDLRMDIEKLTPNSK	NP_847884.2,	XP_00671	15904	TGAccttggcccatctccaccctccaacctggactgcccgccaccagcgcctgcaaccgaactgcagcccaggggtcattgctgcctcaagcctctcggtgcagatgcaccctgaaaactgaccccctcaaacagactgtctgatttgaggatggacattgaaaaactgacgccaaactctaaagaaatgtttatttatacccagggctatcactgtttctaa	0.197	0.373
RT_016579.1	RNF183 ring finger protein 183	EAVLTTELHSK	NP_659488.2	XP_01151	367	TGAgtgctgttcccagacaagaaaccaaaccttttcggttgctgctgggtatggtgactacggagcctcatttggtattgtcttcctttgtagtgttgtttattttacaatccagggattgttcaggccatgtgtttgcttctgggaacaattttaaaaaaaaaacaaaaaaacgaaaagcttgaaggactgggag	0.144	0.257
RT_016183.1	RNF212 ring finger protein 212	SFEVISK		XP_01151	15579	TGAagtttgaagtcatttcaaagacaaagttgtgtttttaccgcgacttccatgcctcctgcgggacctgctcaccttggggcagtgacacctga	0.188	0.153
RT_014053.1	RNF43 ring finger protein 43	SQNFGRPR		XP_01152	14116	TAAtcccagaactttgggaggcctaggtgggtggatcccttgaggccaggagttcaagaccaacctggccaacatggtgaaaccctatctctactaaaaatacaaaaattagccgggtatggtggcgggcacttataa	0.056	-0.075
RT_014228.1	ROBO4 roundabout guidance receptor 4	TVSLRLPR	NP_001288017	XP_00671	6043	TGAaccgtgtccctgagacttcccagacgggaatcagaaccacttctcctgtccacccacaagacctgggctgtggtgtgtgggtcttggcctgtgtttctctgcagctggggtccaccttcccaagcctccagagagttctccctccacgattgtgaaaacaaatga	0.338	0.159
RT_014154.1	RPL13 ribosomal protein L13	SPPGDLESVGSHAGSPR	NP_000968.2, N		3890	TAAagccctcctggggacttggaatcagtcggcagtcatgctgggtctccacgtggtgtgtttcgtgggaacaactgggcctgggatggggcttcactgctgtgac	-0.067	0.033
RT_002043.1	RRAS2 related RAS viral (r-ras) oncogene homolog 2	QMITMLALDEEAG	NP_001170785.1, NP_036		6220	TAGaatcccttcagttttagctaccaacggccaggaaaagccctcatcttctcttctctcctcagtttacatcttgttggtacctttctagccttagacaaatgatcaccatgttagccttagacgaagaagctggctag	-0.013	-0.134
RT_016126.1	SAPCD2 suppressor APC domain containing 2	SLWAAWAPR	NP_848543.2	XP_01151	1984	TAGtccttgtgggccgcgtgggcccccagggccagcctggcactcagcccttcgagggtgggcgccccatcgcacccaccctctctggctggagaccccggcaggcccaggcacagtcccggagtgggcgccttcctgccgcccttgccagatgggctccccaggcctgcccccggctggtccccgcaccgagcgcttga	0.021	-0.119
RT_024884.1	SECISBP2L SECIS binding protein 2-like	LSISSCGR	NP_055516.2, N		5603	TAActcaggaaatgtcggctctctatctccagctgtggaagggttgcagccattaccttttatgcttcatctcaacattttgcactgtccagtatttaa	0.277	0.536
RT_024986.1	SEL1L2 sel-1 suppressor of lin-12-like 2 (C. elegans)	TLLPICHLGDLVPRYE		XP_01152	593	TAGgatgcggatcacaggaaaacctgctcacgggaatcagttcactccaaggtatccccactaaataaacagtttcccatccaaatcaagagtccgctgcagtttcccgctcagggctgtgatgggaaacagacaagacttggggaccatcctagagaaacaacttcagacttcctgcttagtagagaaaacccctttgcgggatgctgggaactggtcacctaagaccttgttaccaatctgtcatcttggtgacctagtcccacgttatgaataa	0.051	-0.226
RT_022798.1	SERINC4 serine incorporator 4	DTQGVISVWAEVNTMNWKK	NP_001244960		5671	TAAgtcctttcacaaactggggttcccctgacattgtactcctagagttggctcaagggggagctgtccagcccagctcaatacctcaaggacacacagggagttatctccgtttgggctgaagtcaatactatgaactggaagaagtggtcaaacacagtctaa	0.178	0.17
RT_023546.1	SETBP1 SET binding protein 1	LHLGPRELTR	NP_056374.2	XP_00525	8012	TAGggcgggtctgggcgtctgcacctggggcctagggaactgacacgtgggaagcgcagtgagccggggcgggggcggaatcccccgctgcagggacacccacgcccttctctccagaagccgggcaggcagaatccggccagacgacggggctga	0.015	-0.103
RT_006972.1	SF1 splicing factor 1	RGEQMVLGK	NP_973724.1, N	XP_01154	143	TGAgttatttttgtggacggaatcggaacacgctggctccatatcgtgaaatttttattaatttttttctttttccttttgttacttctttatcttttcctttcttcagactccgtccaaggagatgctctccccggtcttctgctgcaatttagattcctttcccttctctccagttctccttcccttaccaaggagaggggagcaa	0.323	0.289
RT_014701.1	SGOL2 shugoshin-like 2 (S. pombe)	ESKQKYSIKKMKCLMK	NP_689737.4,	XP_01150	268	TGAagtgaatttatggattctggtttttctgaatttcaaagcataaggaatcaaaacagaaatatagtatcaagaagatgaaatgcttaatgaaaaggtttttttttgtttctttggcctttcatggagtgttgatttgtccattcttaatgtttattaa	0.044	0.153
RT_006725.1	SGSM1 small G protein signaling modulator 1	ERGPGVCSQAWSSVRK	NP_001035037		5791	TGAggggcacctcaccccggcagcctcagccaagctgcccctgccccgctcctctgcttacttttcctcctggctggatgggcaccccgggagcggggtcctggtgtctgttcacaagcgtggagttcagtgcgcaaagaaactaccctgacttttacttctgggcagatggggtggagggagtacccccttcaattcagccttacattttcctgtttga	0.073	0.028
RT_008135.1	SH3RF2 SH3 domain containing ring finger 2	DTNSLASK		XP_00526	13815	je ne trouve pas le peptide		
RT_017390.1	SLC38A8 solute carrier family 38, member 8	WAASAGQEGALR	NP_001073911	XP_01152	918	TGAtgggcagctagtgccgggcaggaaggggccctccgggggctgaccctacgtggctgctgtatgcagccaggagaccgatgccatttcttttcctcataa	0.088	0.065
RT_013448.1	SLC9A3R2 solute carrier family 9, subfamily A (NHE3, cation proton a	TLGLSLPR	NP_001123484	XP_00525	1085	TGAgcccttcctgcctgtctcgggaccctgggaccccctcccgcacggaccttgggcctcagcctgccccgagctcccccagcctcagtggactggagggtggtcctgccattgcccagaaatcagccccagccccggtga	0.091	0.066
RT_018371.1	SLC9A8 solute carrier family 9, subfamily A (NHE8, cation proton ant	GLAEMRASSSPFKT	NP_001247420	XP_01152	5222	TGAcgccaggtgccaaggcttcaggcaggcaggcccaggatgggcgtttgctgcgcacagacactcagcaggggcctcgcagagatgcgtgcatccagcagccccttcaagacataa	0.189	0.485
RT_018499.1	SMOC2 SPARC related modular calcium binding 2	AHTPKAVPR	NP_071421.1, NP_00115		12481	TAAatggctcataccccgaaggcagttcctagacacatgggaaatttccctcaccaaagagcaattaagaaaacaaaaacagaaacacatagtatttgcactttgtactttaaatgtaaattcactttgtag	0.184	0.172
RT_018427.1	SNX29 sorting nexin 29	RHPHGPGCTGELASRGVITHR		XP_01152	6366	TAGcggcatccccatggcccagggtgcacgggggaattagcctctcgcggagtcatcacgcatcgactgaattccctggtgaaaactgagttagccagttgttcctaa	0.251	0.171
RT_021657.1	SPACA7 sperm acrosome associated 7	SLLEPPPTSLRNR	NP_660291.2,	XP_01153	13958	TGAggccgcagccccagacccccctgcgcaggagaggagcctgctagaacccccacccaccagcctccggaacagggcacttgtgtgcacacgcccacgttctctgaaccattccacataa	0.012	0.119
RT_009922.1	SPAG11B sperm associated antigen 11B	AGMLKYPK	NP_478108.2, N	XP_00527	335	TAAaatataagctcccggaaggcagggatgttgaagtatcccaagggcttaaaggaatgtgtggcttatagtaggtgttcaataaatatttgttga	-0.046	-0.089
RT_005096.1	SPAM1 sperm adhesion molecule 1 (PH-20 hyaluronidase, zona pellu	LAEMNNMSILK	NP_001167515	XP_01151	3094	TAAttgcgcaggttagctgaaatgaacaatatgtccatcttaaagtgtgctttttcgactaattaa	0.092	0.116
RT_002817.1	SPATA21 spermatogenesis associated 21	QELLLGFAFVAW	NP_940948.1,	XP_01153	4868	TGAccctctggaggcctgactctaggcagctgctcaaggcctgtagacagatgggcccaccaggcaggagctgctgctgggatttgcttttgtggcctggtga	0.219	0.432
RT_006931.1	SPDYE3 speedy/RINGO cell cycle regulator family member E3	HLYSQLLK		XP_01151	2077	je ne trouve pas le peptide		
RT_019025.1	SPOCK3 sparc/osteonectin, cwcv and kazal-like domains proteoglycar	LMTVEINK	NP_001238896	XP_01153	588	TGAttgatgacagttgaaatcaataaattctacatttctaatatttacaaaaatgatagcctatttaaaattatcttcttccccaataacaaaatgattctaaacctcacatatattttgtataattatttgaaaaattgcagctaa	0.272	0.294
RT_004780.1	SPTB spectrin, beta, erythrocytic	QVGLAGAAESGRAETGQPRPWPPL	NP_001020029	XP_00526	11484	TAGcaggtggggctggcgggcgcggcggagtcgggacgcgcagagaccgggcagcccaggccttggcctcctctcgccgccgcccgccggtcgaatcgagatgagtcgagtcgcgggccgggcccccgggccccacgctgcactgcaaaagctgccttcgcccgcgtgtcctgggccgacgcccccctccatgccctgcccgcgctggctccgctccagggacaggggctcccagctcctctggcgcctttggggcctccaggcctgggggcaggaaacggttctctgaggcacacgccctcctctcccaccctcctcctgagccgagggggcacaggcccccttacccccaaccaaaaaaaaaaaaaaaaaaaaagagctggagaaggctgatggggttcttcaaacgctgcccgttcctaaagcaagtcttgcttcgggtcacctcccacctggtggcagccagggaaaggggaaaggaagaagacactggaaatgcatggccagccccctaggggcatga	0.137	0.107
RT_026612.1	STIL SCL/TAL1 interrupting locus	PFNSLPF	NP_003026.2, N	XP_01154	11171	TAAccttttaactccctgcccttttaa	0.112	0.195
RT_009155.1	SUPT7L SPT7-like STAGA complex gamma subunit	EMFCPDLLDPTEK	NP_001269661	XP_00526	6091	TAAaggaaaagagggagatgttttgtccagacctactagacccaacagaaaaggtttttgtattagaatctgtttccttaaaaattgatttgactcctgttcttaaacacaagtggtttttcctaattccagaggaactggacgtcaccaaacaaggttgcattttactttgcatccagttttatagttttccaca	0.058	-0.122
RT_010002.1	SWI5 SWI5 homologous recombination repair protein	AGSSPLVHSSQGQK	NP_001035100	XP_00525	5056	TGAgcaggctcatcgcccccttgtccacagctcccagggacagaagggtgtggacatgataaacactgagagcccaaccagcacacctacagagtttccagcgagacaatgccagaagcacttttctagagagcccaagcccaatcaggaggaggctagagaagaggcgggcagggaggaagggggcgttcccagctccaaggttcctgaaatgtcgcagtgatacttgtgtaggggtacatgtactttatttgtcaataaacgtatctgtacactga	0.325	0.292
RT_002713.1	SYCE2 synaptonemal complex central element protein 2	CAGHCFRSTDQQR		XP_01152	5976	TGAcaccttggggtcagatgtgcaggccactgcttcagaagtacagaccaacagagatggtga	0.325	0.421
RT_026555.1	SYT15 synaptotagmin XV	PFAQHRGSALGADHPR	NP_114118.2,	XP_01153	12022	TGAcccttcgcccagcaccgcggttccgctttgggagccgaccatcctcgcagttgcatgtga	0.336	0.432
RT_026388.1	T T brachyury transcription factor	AQVPKDAVTFCRGSQW	NP_001257413	XP_01153	4595	TGAagcagcaaggcccaggtcccgaaagatgcagtgacttttttgtcgtggcagccagtggtga	0.237	0.211
RT_019228.1	TCTE3 t-complex-associated-testis-expressed 3	MVWLRLR		XP_00671	20255	TAGctcattacagattcagacaagaagtgcggcggaagcttacacctgcacgtggtgggacagaggcaagaacggatggtctggctgaggctgagagaaccaagctggattaggatcggcttgagggcccggcgtcgtcagtggtga	0.379	0.491
RT_007777.1	TET3 tet methylcytosine dioxygenase 3	AVSVVLLPSYLGAGWGCR	NP_001274420	XP_01153	13737	TAGgtgcccagggagccagcgtacctcagcgtcgggcctggcccgagctgtctctgtggtgcttttgccctcatacctgggggcgggttgggggtgcagaagtctttttatctctatatacatatatagatgcgcatatcatatatatgtatttatggtccaaacctcagaactgacccgcccctcccttacccca	0.008	0.035
RT_026131.1	TEX26 testis expressed 26	IVQMKK	NP_689538.1,	XP_01153	1363	TGAaaagggaaaatagtacaaatgaagaaaatctga	0.169	0.373
RT_013769.1	THUMPD2 THUMP domain containing 2	TVTQMNLELK		XP_00671	7176	not found		
RT_001050.1	TMEM106B transmembrane protein 106B	KLEEMDLK	NP_060844.2, N	XP_00524	1964	TAAaaactggaagagatggatttaaagaagaaatatctattgatatttcctatactctcaatgaagaggtatttcctaataggagaccttaaattgaacaaacctaaagtttacacttctaagagtacagttaaaagtatgtggacctgcagttcttgtaactctccactctgtgttaatgatatatttgtactaggatcttttactga	0.046	0.195
RT_012578.1	TMEM155 transmembrane protein 155	RMTSHSHLQNLK		XP_01152	121	not found		
RT_017166.1	TMEM185A transmembrane protein 185A	WTGTHSLR	NP_001167563		1538	TAGatgccacttccggggacagagcttaagtggactgggacgcactctctccgccttcctctgccccctcgttcaccccgcagaccagaaccagtactggagctgggtctccaggtacgtccatctcatgccttgtttgcatccagcgcctatcagccactcaccacgacgggacgcggaagtggcaggtga	0.008	0.127
RT_019558.1	TMEM54 transmembrane protein 54	TLQATLLCDQARIPGAGLR	NP_277039.1	XP_00527	13000	TGAgagatgatgtcctgcccaggcccgatggccactaggaccctgcaagcaactctgctctgtgaccaggccaggattcctggagctggcctgagagggctcaatggaccctcggggacccaagtggggctttcaaccctctcccccaccacccagcccactgcactgaaatga	0.14	-0.036
RT_022387.1	TMEM92 transmembrane protein 92	VTSCLESCHQQPPPQCLLDQAR	NP_001161687	XP_00672	5318	TGAgtcacctcctgcctggaatcttgccatcagcaacctcctccccagtgcctcctggatcaagctagagactgctggcaccccaggaatgtccctgcccatcctgccgtgtctctgttcattcttggatttaacttattactttttctgcttctgtttccaccccagctgcctctcttgtcctga	0.304	0.348
RT_004139.1	TNFRSF25 tumor necrosis factor receptor superfamily, member 25	RSGGPCRSPK	NP_683871.1, NP_68386		10644	TGAcacggcgcccacttgccacctaggcgctctggtggcccttgcagaagccctaagtacggttacttatgcgtgtag	0.343	0.421
RT_024642.1	TNFSF14 tumor necrosis factor (ligand) superfamily, member 14	GALDMGLTRGELR	NP_742011.2, N	XP_00525	5902	TGAaggaaggagcgtggtgcattggacatgggtctgacacgtggagaactcagagggtgcctcaggggaaagaaaactcacgaagcagaggctgggcgtggtggctctcgcctgtaa	0.123	0.12
RT_019102.1	TNK2 tyrosine kinase, non-receptor, 2	DASGEPEGLPEGIT	NP_005772.3, N	XP_00526	7353	TGAgatgcgtctggagagccagagggcctgcctgaaggaatcacctga	0.092	-0.004
RT_005492.1	TOX4 TOX high mobility group box family member 4	SYLLGKCPR	NP_055643.1, NP_00129		1913	TAGtccttcctgttctccaagccagtgaagagttatctgctgggaaagtgtccaagagcctgtttttga	-0.102	-0.119
RT_015033.1	TPCN1 two pore segment channel 1	APEGPSQGDPSPGSSR	NP_001137291	XP_01153	8686	TAGcccagcgcccgaaagccgtctcttctatgcaataacacaatagtattactctactgcgatgtacggaactgcggtgtgtgtacacatactcacgtatatgcacatatttatatacaggaagaaaaaagacagacaagatggggcttggtttataaccaccttgccctgtcttccttaactccagaagcca	0.143	0.21
RT_019778.1	TPCN2 two pore segment channel 2	LAYTGAR	NP_620714.2,	XP_00527	9965	TGAcgtccgggctgccgtcccagcaggggcggcaggagagagaggctggcctacacaggtgcccgtcatggaagaggcggccatgctgtggccagccaggcaggaagaccctttcctctgacggaccactaagctggggacaggaaccaagtcctttgcgtgtggcccaacaaccatctacagaacagctgctggtgcttcagggaggcgccgtgccctccgctttcttttatagctgcttcagtgagaattccctcgtcgactccacagggacctttcagacaaaaatgcaagaagcagcggcctcccctgtcccctgcagcttccgtggtgcctttgctgccggcagccctt	0.186	0.426
RT_002189.1	TPM1 tropomyosin 1 (alpha)	PWFSLLASCLRARHTLCFLLYR		XP_00525	2616	TGAccctggttctctctcttagcatcctgccttagagccaggcacacactgtgctttctattgtacagaagctcttcgtttcagtgtcaaataaacactgtgtaa	0.262	0.432
RT_024775.1	TPM3 tropomyosin 3	LSPFLLCSGSAPFTPR	NP_689476.2,	XP_00671	2836	TAAttatcaccgtttctgctctgttctggatctgcccccttt actcctcggggaacccaaggccccactctcgctctggattccatttgggtcagcctggctggtccccaaggcattaggatgggggagcaaaaagcaacttatgtattttcttccacccccaccccaaattaa	0.264	0.433
RT_000591.1	TPRA1 transmembrane protein, adipocyte asscociated 1 (lipid metab	EEASRPRVPRGGGPGQGTFCGQ	NP_001136118	XP_01151	66	TGAcatcgcttccatgccctgccacactggcagcatcaacagcacagacagcgagcgctggaaggccatcaatgcctgagggcagctgccagggcctgtggaggacaggccagagaggaggccagcaggcccagagtccccaggggaggaggaccaggtcaagggacgttctgtgggcagtagc	0.262	0.362
RT_020019.1	TRIM68 tripartite motif containing 68	GLELGLAPMGLGGPSH	NP_001291425.1, NP_060		6601	TAAgaaagctaccaccctaaccagagaggcttggaattggcctggcccccatggggcttggaggaccgagccactga	0.15	0.103
RT_006402.1	TRIM7 tripartite motif containing 7	VTVANRAGEQGLWTPEGVPFLRSH	NP_976038.1, NP_97603		3507	TGAggggcactgctggggagctcctgtctctgggctgccggtgggaggggatgtcgcctccccagagatgcctggtccgtcttgggtctgccctccgtgctcctgaccctgctgcccaagagagcctgctacagacacaaccccgaggcaggagagtgactgtggccaaccgagcaggggaacaggg	0.102	0.028
RT_014420.1	TSPEAR thrombospondin-type laminin G domain and EAR repeats	GRGCPAATGVAR	NP_659428.2, N		422	TGAggccgcggctgcccggcagcaactggggtggccaggtggggcgggaccccagcacctcccccaactgaccctggacgtccaggtgggcccagcctagggcatcccgggcctgggcatcaggcagggtgcaggtggggccatctgcggccctggttcgggcagggacatccatccacctctgagtccttctga	0.073	0.119
RT_013052.1	TSPO translocator protein (18kDa)	VPGPPGTAAAPAGAITLVMWWPSF	NP_000705.2, N		1818	TGAgtgcccggcccaccagggactgcagctgcaccagcaggtgccatcacgcttgtgatgtggtggccgtcacgctttcatgaccactgggcctgctag	0.183	0.257
RT_008971.1	TSTA3 tissue specific transplantation antigen P35B	TGSGASGPSAGR	NP_003304.1,	XP_01151	9638	TGAagctggaagacaggatcaggtgccagcggaccatcggctggcagagcccagcggccaccacccgtcaaccctgccaggagctga	-0.078	0.211
RT_021416.1	TTC13 tetratricopeptide repeat domain 13	LFFKTWNYK	NP_001116307	XP_00671	3290	TAGttctgctgctgtatttatacaagtaaagggccggacctcttgcttcttaagttatttttaaaacatggaattataaagaaattaggtcctctattacttttgataccaattttataggaattga	0.125	0.162
RT_021425.1	TTC39B tetratricopeptide repeat domain 39B	WKNDLVNK	NP_001161813	XP_01151	4525	TGAtcagaacttgaaggatggaatttttcctcaattagcatcggcatctgctatagattcaaccttatattaaagtggaaaaatgatcttgtgaataagagacaaaaaaacagttttttggtcaatattttctatcatctgagagatttactgttggtctacataatttttttcctatggaatggcattctatattatc	0.324	0.329
RT_003336.1	TTC7A tetratricopeptide repeat domain 7A	QQGTWVR; REGGAGQR	NP_001275882	XP_01153	17188	TGAcgacgctgcagccgcagggagggaggggctggccagagggagaggcagcagggaacgtgggtcagggtggggcaacagtggcatcaggtgcggggcctcagggaaatacatctttag	0.4	0.71
RT_022743.1	TUBGCP2 tubulin, gamma complex associated protein 2	KGVGSAGTGANGSR	NP_001243547		6900	TGAgccctggctgtgacaggaaggaagggtgtggggtcagcagggactggtgcaaatgggtccagaattttcaaatcgaatgctctgtgttctgtctttgcagtttaa	0.052	0.066
RT_023684.1	TUFT1 tuftelin 1	AISIFLQ	NP_064512.1, N	XP_01150	16246	TGAgctgcctggagatggttgctgccattgctgctgcctctgcctcggagaagcccactgccccttgttggctgttaacactgcctttgacttcctgactgtcccctggctgcacccaggacttcgggctcctgtgtctcaccattcccaagcccctggccactctaagctgggcagacggagcacgagcacctattcaaggcactgcagccctttggaagacattgtcctgcaagcaggagccagggcaatatctatattcctacagtga	0.196	0.008

Biology Open • Supplementary information

RT_007176.1	UBA2 ubiquitin-like modifier activating enzyme 2	TEMPLNRTLLLFSLSGQNQIVMSFV	NP_005490.1,	XP_00525	2127	TGAacagaaatgcctctaaacagaaccctcttactatttagtttatctgggcagaaccagattgttatgtcctttgttcc<br>aaagggaaaaaattgacagcagtga	0.25	0.384
RT_009758.1	UBL5 ubiquitin-like 5	RILIFLPR	NP_001041706.1, NP_077		9141	TAGatgagaatcctcatcttcctgccccgctttcctctcccatcctcatcccccacactgggatag	0.097	0.127
RT_022085.1	uncharacterized LOC105371419	APPGHSDRGRGVR		XP_01152	4152	TAAcagccacacccgaccagtgacagcagggccctggccagggctcggggggtcacctggggcaagggacctccagagaggaatctgagggcccctcctgggcacagtgacagagggcggggtgtgaggaagccgactggaagagccgctgttcacacaa<br>attcccagagggaagggcagggaaggacagcgtag	0.172	0.152
RT_000368.1	UNG uracil DNA glycosylase	SSAEGWPLR	NP_550433.1, NP_00335		4500	TGAtcatcagctgaggggtggcctttgagaagctgctgttaa	0.157	0.329
RT_023844.1	UVSSA UV-stimulated scaffold protein A	AGPSALAISTFSLCQCLR	NP_065945.2,	XP_01151	5089	TAGagagcggggcccagtgcactggccatcagcactttctccctctgccagtgtctcaggacagcagagtgggcgtgggt<br>ctgggcagtaaccatgctttgtctattactgtgtttgatgtaaa	0.028	0.215
RT_012400.1	VASP vasodilator-stimulated phosphoprotein	TTQECIVPTPHPTWK	NP_003361.1,	XP_00525	921	TGAccacagggacccagaagacccgcttctcctttccgcacacccggcctgtcaccctgctttccctgcctctacttgacttggaattggctgaagactacacaggaatgcatcgttcccactccccatcccacttggaaaactccaaggggggtgtggcttccctgctcacacccacactggctgctga	0.494	0.658
RT_001270.1	VDAC3 voltage-dependent anion channel 3	ASDLSLEVKRNEPTMFWP	NP_001129166	XP_00671	4076	TAAtgtggtttgaggaaagcatcagatttgtccctggaagtgaagagaaatgaacccactatgttttggccttaa	0.149	-0.081
RT_022463.1	VMO1 vitelline membrane outer layer 1 homolog (chicken)	AAVVSRR	NP_001138413.1, NP_001		21723	TGAgccgctgtggtgtcgcggcggcgcctacctagtggctttctcgcttcgcgtggaggcacccacgaccctcggtga	0.064	0.066
RT_017770.1	VPREB1 pre-B lymphocyte 1	SAEEAEPLSISWSCSVAVNPHWR	NP_009059.1, NP_00129(		4587	TGAactgaagacagcagaggcacgcatccccttggagagactgtcatggaagagggtggagtcgccgcccgaagcgccgaggaggctgagccactcagcatctcctggtcctgcagtgttgctgtaaatccccattggagactgcattag	0.129	0.1
RT_013079.1	WDFY3 WD repeat and FYVE domain containing 3	FNKLSGDHGL	NP_055806.2,	XP_01153	7738	TGAagattcaacaagctgagtggagaccatggtctgtag	0.237	0.437
RT_019157.1	WDR19 WD repeat domain 19	ADTMLLR	NP_079408.3,	XP_01151	8174	TGAttggcacgtgcagatacaatgctcctgagaagacagcattttccacaggaggctgtttcctcccctggtggatttaa<br>gagacggtcctttctggatacagagaaatga	0.269	0.294
RT_004152.1	WNT7A wingless-type MMTV integration site family, member 7A	MLSLSFLLR	NP_004616.2	XP_01153	7432	TGAgccccgtgtgcacaccaccctcccgctgcaagtcagattgctgggaggactggaccgtttccaagctgcgggctccc<br>tggcaggatgctgagcttgtcttttctgctgaggagggtacttttcctgggtttcctgcaggcatccgtgggggaaaaaa<br>aatctctcagagccctcaactattctgttccacacccaatgctgctccaccctcccccagacacagcccaggtccctccg<br>cggctggagcgaagccttctgcagcaggaactctggacccctgggcctcatcacagcaatatttaac	-0.001	0.066
RT_022489.1	ZCCHC5 zinc finger, CCHC domain containing 5	GGPHVGQSTNMRPPLFQYPCLIHY(	NP_689907.1	XP_01152	9291	TAAgggggaccccacgtgggccaatctacaaatatgcgtcccccctctcttccaatatccatgtctcatacactatggcttactgttgaaatccaactgggaagaagataattctttgagcaagcaatggtag	0.007	-0.15
RT_023327.1	ZNF140 zinc finger protein 140	NYECMEFFKK	NP_001287707	XP_01153	894	TGAatttacactgcaaagaaaaactatgaatgtatggaattttttaaaaagaagtataatgccttacttcagagaactct<br>tggaaagaagccttatgtgaaagtgatgactgtgaagtaa	0.218	0.382
RT_027047.1	ZNF282 zinc finger protein 282	GAERPRAPPSHLLECPGTTGPQMV	NP_003566.1,	XP_00671	5072	TAGggctgggctgggggagggcagggccggacggagtggatcgggggcggcctgagcaccaaccaccttgccgggtgtcctcagccaccgtctggaaatcggcaacaggcattgcactccggttgggggtcccccagggtggggcagggatcccccagat<br>ctgtctggtctgaatggacgcccagctcatctagggtggacccagctgctggggaagagccagggggaccgcgaggagcc<br>gagcgtcctcgggcaccgccctcacacctcctcgagtgccctgggaccactgggccacagatggtcatcaggggaagcca<br>ccagggagtcccgaagcccttctga	-0.116	-0.103
RT_014705.1	ZNF283 zinc finger protein 283	VVKEYFVCVYRQLIIIRTLTLEK	NP_001284681	XP_00525	4338	TGAttgaaagttgtaaaagaatattttgtgtgtgtgtatagacaacttatcataataagaactcttactcttgagaaaccttgtgaatgtaagggttgtgcaaaagccattcatttctgtttatgggcaattatcttgctatccagcaattcatactagtga	0.227	0.294
RT_010661.1	ZNF34 zinc finger protein 34	FIAADLR	NP_001273699.1, NP_085		7548	TAAcagtggcgcggtgtccgagggcagagtccagctgagcacttcctgcatgcgcccccggcacctgactctgccctttatgtattatccacacgatgttttcacagagtgaaaggacgtttctcattaaacaaacctcttttcttaaatcaaaagcagtgcatgttcattttagagaaattgagacagaaaag	0.201	0.152
RT_002045.1	ZNF410 zinc finger protein 410 (glycosylation candidate)	ATLSDLKMR	NP_067011.1, NP_00122		5300	TGAgcgtgggtgctgactcctggaagagcaactctatctgatctcaaaatgcgtatactgggaacaggatgccttagcccacaacagaaccagaatgaatctttgaaggcacaagactctgcttttgccactcttcctctttcctggtatagaagatggatgtag	-0.098	-0.025
RT_023991.1	ZNF512B zinc finger protein 512B	VALPLLR	NP_065764.1,	XP_00526	3859	TGAgcatggtgcgtggggcctggcccccatgcagccgccacaccgccctctgttcaaggcagggccagctccgggacctc<br>tgtcctccagttctccatcccccacccccgcctcttcatccgtccagtggggcagccagccactctcccttccctgaaggtggcccttcccctgttaaggctgccacagggcacacctgggttggggctccccaaggtgtgcccagcagaagtgcaatag	0.074	0.292
RT_010258.1	ZNF580 zinc finger protein 580	RGVGAPAPGGGTSALGSR		XP_01152	1229	not found		
RT_005360.1	ZNF584 zinc finger protein 584	RATESSSTRTLIVR		XP_00525	8735	TAAagagctacagagtcatccagcaccaggacactcatagtgaggggaaaccaagaaggcacactgagcatggggcagct<br>ttcccacctggttccagttgtgggcaacagcaagaagtccatgtggcagagaagctgttcaaatgcagtgactgtgggaa<br>ggtgttcttaa	0.176	0.259

Biology Open • Supplementary information

Table S4.

Relative Fluc fold change

UGA Fig 1F

Condition	N1 Norm Fluc/Rluc	N1 SD	N2 Norm Fluc/Rluc	N2 SD	N3 Norm Fluc/Rluc	N3 SD
Vector	1.0000	0.2409	1	0.2587	1	0.3428
H-Ras V12-D5	1.0940	0.3269	0.5199	0.2041	0.9202	0.1283
H-Ras V12-D12	0.5663	0.1330	0.3747	0.1497	0.4701	0.0662
H-Ras V12-D20	0.4043	0.0241	0.2683	0.1672	0.3423	0.1405

AQP4 Fig 1F

Condition	N1 Norm Fluc/Rluc	N1 SD	N2 Norm Fluc/Rluc	N2 SD	N3 Norm Fluc/Rluc	N3 SD
Vector	1.0000	0.1645	1	0.1701	1	0.1370
H-Ras V12-D5	1.7850	0.6203	0.8558	0.1782	1.828	0.3805
H-Ras V12-D12	0.7813	0.0213	0.3300	0.0806	0.7042	0.1719
H-Ras V12-D20	0.3594	0.0807	0.2036	0.0492	0.4357	0.1050

UGA Fig 2A

Condition	N1 Norm Fluc/Rluc	N1 SD	N2 Norm Fluc/Rluc	N2 SD	N3 Norm Fluc/Rluc	N3 SD
Vector	1.0000	0.1771	1	0.1162	1.0000	0.0238
H-Ras V12	0.4557	0.1307	0.2343	0.1541	0.6687	0.1065
Vector Gen	1.9220	0.5004	11.3100	1.07700	5.5810	0.0345
H-Ras V12 Gen	1.1860	0.1066	7.9870	1.61800	4.3510	0.6618

AQP4 Fig 2A

Condition	N1 Norm Fluc/Rluc	N1 SD	N2 Norm Fluc/Rluc	N2 SD	N3 Norm Fluc/Rluc	N3 SD
Vector	1.0000	0.0864	1	0.2858	1.0950	0.1338
H-Ras V12	0.3274	0.1348	0.5268	0.0705	0.2753	0.0467
Vector Gen	2.4020	0.4240	1.5830	0.0189	2.6290	0.6564
H-Ras V12 Gen	0.9056	0.0795	1.4380	0.3512	0.9912	0.0870

MMuLV Fig2A

Condition	N1 Norm Fluc/Rluc	N1 SD	N2 Norm Fluc/Rluc	N2 SD	N3 Norm Fluc/Rluc	N3 SD
Vector	0.7927	0.07473	1.0000	0.1428	1.1450	0.2049
H-Ras V12	1	0.00017	1.3520	0.000165	1.3150	0.147
Vector Gen	0.7581	0.07355	1.0250	0.07029	1.1050	0.3146
H-Ras V12 Gen	1.046	0.1354	1.3480	0.09148	2.0100	0.0386

UGA Fig 2B

Condition	N1 Norm Fluc/Rluc	N1 SD	N2 Norm Fluc/Rluc	N2 SD	N3 Norm Fluc/Rluc	N3 SD
Vector	1.1310	0.1031	0.8210	0.0169	1.0000	0.1838
PML-ER	1.0000	0.2771	1.0000	0.1238	0.9085	0.2310
Vector 4OHT	1.1430	0.1341	0.8441	0.1567	0.9875	0.0128
PML-ER 4OHT	0.0657	0.1138	0.0000	0.0000	0.9542	0.3150
Vector Gen	15.9600	4.3580	17.1100	3.2800	17.9200	2.8720
PML-ER Gen	11.0100	1.1130	17.9700	3.8380	16.8300	2.5950
Vector 4OHT Gen	14.6700	1.6520	19.1300	4.4710	11.5500	0.3844
PML-ER 4OHT Gen	2.2040	1.2050	3.9580	0.6262	6.0300	0.6035

AQP4 Fig 2C

Condition	N1 Norm Fluc/Rluc	N1 SD	N2 Norm Fluc/Rluc	N2 SD
Vector	1.0000	0.0554	1.0000	0.1457
PML-ER	0.8622	0.0583	0.9104	0.1158
Vector 4OHT	1.1640	0.2160	1.0570	0.1785
PML-ER 4OHT	0.6573	0.0927	0.7367	0.1292
Vector Gen	3.7630	0.7502	2.5350	0.2966
PML-ER Gen	2.9520	0.3866	2.9780	0.2403
Vector 4OHT Gen	2.7590	0.4541	2.4750	0.1161
PML-ER 4OHT Gen	1.8230	0.1799	2.0780	0.6261

MMuLV Fig 2D

Condition	N1 Norm Fluc/Rluc	N1 SD	N2 Norm Fluc/Rluc	N2 SD
Vector	1.0000	0.1017	1.0000	0.07772
PML-ER	0.8475	0.0784	0.8101	0.1826
Vector 4OHT	1.0120	0.1778	1.1320	0.2045
PML-ER 4OHT	0.8001	0.1193	0.8046	0.09078
Vector Gen	1.4700	0.1510	1.5350	0.4673
PML-ER Gen	1.3340	0.0510	1.2730	0.01816
Vector 4OHT Gen	1.5010	0.0460	1.4530	0.04499
PML-ER 4OHT Gen	0.8767	0.1930	0.9071	0.1145

Relative Fluc fold change

UGA Fig 3B

Condition	N1 Norm Fluc/Rluc	N1 SD	N2 Norm Fluc/Rluc	N2 SD
Vector D12	1.0532	0.2135	1.0000	0.1817
H-Ras V12 D12	0.6578	0.1054	0.7406	0.1223
Vector D35	1.0000	0.0579	1.0000	0.0579
H-Ras V12 D35	0.9209	0.0639	1.6336	0.1719

AQP4 Fig 3B

Condition	N1 Norm Fluc/Rluc	N1 SD	N2 Norm Fluc/Rluc	N2 SD	N3 Norm Fluc/Rluc	N3 SD
Vector D12	1.0000	0.0864	1.0000	0.0479	1.0000	0.2210
H-Ras V12 D12	0.3274	0.1348	0.5905	0.0455	0.4175	0.1630
Vector D35	1.0000	0.0537	1.0000	0.1802	1.0000	0.1230
H-Ras V12 D35	1.5119	0.3644	1.8362	0.2394	1.2496	0.2135

VASP Fig 3B

Condition	N1 Norm Fluc/Rluc	N1 SD	N2 Norm Fluc/Rluc	N2 SD	N3 Norm Fluc/Rluc	N3 SD
Vector D12	1.0000	0.0120	1.1411	0.3336	1.0000	0.3467
H-Ras V12 D12	0.7189	0.0187	1.0000	0.2572	0.5411	0.0613
Vector D35	1.0000	0.2016	1.0000	0.3699	1.0000	0.2288
H-Ras V12 D35	2.9382	0.3314	2.2841	0.2431	2.9616	0.8335

HPN Fig 3B

Condition	N1 Norm Fluc/Rluc	N1 SD	N2 Norm Fluc/Rluc	N2 SD	N3 Norm Fluc/Rluc	N3 SD
Vector D12	1.0000	0.0505	1.0000	0.1064	1.0000	0.1580
H-Ras V12 D12	0.5396	0.1724	0.4845	0.6852	0.8584	0.1746
Vector D35	1.0000	0.0299	1.2092	0.4646	1.0000	0.3842
H-Ras V12 D35	3.3707	0.3539	1.6540	0.3531	1.6540	0.3531

UGA Fig 4A

Condition	N1 Norm Fluc/Rluc	N1 SD	N2 Norm Fluc/Rluc	N2 SD	N3 Norm Fluc/Rluc	N3 SD
Vector	1.0000	0.1751	1.0000	0.0968	1.0000	0.2026
H-Ras V12	0.3895	0.2037	0.6659	0.1597	0.1657	0.0743
H-Ras V12/E6	0.4181	0.0877	0.8446	0.1520	0.8052	0.1544
H-Ras V12/E7	2.3978	0.4075	3.9306	0.4586	3.6125	0.1093
H-Ras V12/E6/E7	5.8209	1.1219	2.0074	0.0243	1.6591	0.3204

AQP4Fig 4B

Condition	N1 Norm Fluc/Rluc	N1 SD	N2 Norm Fluc/Rluc	N2 SD	N3 Norm Fluc/Rluc	N3 SD
Vector	1	0.1443	1.045	0.1559	1	0.1168
H-Ras V12	0.2937	0.076	0.8011	0.07103	0.6803	0.1386
H-Ras V12/E6	0.3333	0.07278	0.9688	0.0749	0.6761	0.1346
H-Ras V12/E7	0.5688	0.09934	1.133	0.13	1.147	0.05639
H-Ras V12/E6/E7	0.9557	0.3249	1.374	0.04987	1.375	0.2305

VASP Fig 4C

Condition	N1 Norm Fluc/Rluc	N1 SD	N2 Norm Fluc/Rluc	N2 SD	N3 Norm Fluc/Rluc	N3 SD
Vector	1	0.3191	1	0.2039	1	0.1326
H-Ras V12	0.4874	0.02815	0.4709	0.0702	0.7801	0.07476
H-Ras V12/E6	0.6765	0.01425	0.4316	0.01853	0.9158	0.05154
H-Ras V12/E7	1.844	0.3842	0.8383	0.1091	1.342	0.1684
H-Ras V12/E6/E7	1.536	0.11	1.517	0.0321	1.507	0.242

AQP4 Fig 4D

Condition	N1 Norm Fluc/Rluc	N1 SD	N2 Norm Fluc/Rluc	N2 SD	N3 Norm Fluc/Rluc	N3 SD
Vector	1	0.1713	1	0.1036	1.045	0.1559
H-Ras V12	0.3133	0.06554	0.5884	0.05405	0.8011	0.07103
H-Ras V12 /E7	0.5997	0.1068	0.8384	0.0461	1.133	0.13
H-Ras V12/ E7Δ21-24	0.2692	0.0311	0.3682	0.0506	0.8253	0.08496

AQP4 Fig 4E

Condition	N1 Norm Fluc/Rluc	N1 SD	N2 Norm Fluc/Rluc	N2 SD	N3 Norm Fluc/Rluc	N3 SD
Vector	1	0.06915	1	0.2102	1	0.0437
H-Ras V12	0.5729	0.07417	0.7011	0.05802	0.66	0.01713
Vector /CDK4	0.9616	0.2299	1.127	0.3828	1.032	0.139
H-Ras V12/CDK4	0.9731	0.08102	1.397	0.1968	0.6475	0.1597

Relative Fluc fold change

UGA Fig 5A

Condition	N1 Norm Fluc/Rluc	N1 SD	N2 Norm Fluc/Rluc	N2 SD	N3 Norm Fluc/Rluc	N3 SD
Vector	1	0.08361	1	0.3002	1.076	0.2082
Vector Palbo	1.372	0.1402	0.8938	0.1501	0.9886	0.1107
Vector Gen	20.4	2.396	10.59	1.821	7.828	1.838
Vector Palbo + Gen	11.23	1.73	1.708	0.1734	5.699	1.172

AQP4 Fig 5B

Condition	N1 Norm Fluc/Rluc	N1 SD	N2 Norm Fluc/Rluc	N2 SD	N3 Norm Fluc/Rluc	N3 SD
Vector	1.0000	0.1549	1.0000	0.1802	1.0450	0.1230
Vector Palbo	0.7022	0.0392	0.9950	0.2310	0.7346	0.0666
Vector Gen	1.5200	0.2612	4.8140	0.5472	3.9550	0.5800
Vector Palbo + Gen	1.3210	0.1188	5.2490	0.7914	1.8240	0.3738

AQP4 Fig 5C

Condition	N1 Norm Fluc/Rluc	N1 SD	N2 Norm Fluc/Rluc	N2 SD	N3 Norm Fluc/Rluc	N3 SD
Vector D35	1	0.1425	1	0.123	1	0.2231
H-Ras V12 D35	1.466	0.1292	1.25	0.2135	1.178	0.147
Vector D35 Palbo	0.573	0.2292	0.7346	0.06662	0.2108	0.02081
H-Ras V12 D35 Palbo	0.5395	0.08137	0.7351	0.1591	0.3108	0.05771

UGA Fig S1

Condition	N1 Norm Fluc/Rluc	N1 SD	N2 Norm Fluc/Rluc	N2 SD
Vector D5	1.0000	0.2828	1.0820	0.1161
Vector D12	0.8620	0.3315	0.9551	0.1077
Vector D20	1.0000	0.1937	0.8995	0.2043

AQP4 FigS1

Condition	N1 Norm Fluc/Rluc	N1 SD	N2 Norm Fluc/Rluc	N2 SD
Vector D5	0.9991	0.2331	0.931	0.1098
Vector D12	0.7887	0.1582	1.004	0.2469
Vector D20	0.7829	0.1559	1.066	0.2165

AQP4 FigS1E

Condition	N1 Norm Fluc/Rluc	N1 SD	N2 Norm Fluc/Rluc	N2 SD
Vector/Starv	1	0.06281	1	0.04788
H Ras V12/Starv	0.4751	0.05073	0.5905	0.04545
Vector /FBS	0.928	0.2528	0.8961	0.213
H Ras V12/FBS	0.5003	0.1221	0.669	0.01713

Nat. Rth candidates Ctrl Fig S

Condition	N1 Norm Fluc/Rluc	N1 SD	N2 Norm Fluc/Rluc	N2 SD	N3 Norm Fluc/Rluc	N3 SD
HPN-Vector	1	0.075251	0.9999999	0.050515	1	0.253091
HPN-H-Ras V12	0.3838983	0.384538	0.7287598	0.218933	0.5758567	0.060332
VASP-Vector	0.9999998	0.008484	0.9999998	0.292373	1	0.008485
VASP-H-Ras V12	0.7492877	0.05589	0.8763331	0.225433	0.7188824	0.018714
ASPH-Vector	0	0	0	0	0	0
ASPH-H-Ras V12	0	0	0	0	0	0
FBL-Vector	1	0.178778	0.9999999	0.197851	1	0.144868
FBL-H-Ras V12	0.6416453	0.164848	0.8305688	0.006193	0.9574316	0.076049

Nat. Rth candidates Gen Fig

Condition	N1 Norm Fluc/Rluc	N1 SD	N2 Norm Fluc/Rluc	N2 SD
HPN-Vector	8.0240	0.9762	3.6987	0.4123
HPN-H-Ras V12	7.9446	2.6363	2.4933	0.1470
VASP-Vector	19.0749	1.8238	4.4482	0.3532
VASP-H-Ras V12	8.2412	1.1154	3.1289	1.0182
ASPH-Vector	3.6987	0.4123	1.0000	0.4716
ASPH-H-Ras V12	2.4933	0.1470	0.1653	0.0323
FBL-Vector	3.1437	0.4392	1.8573	0.3116
FBL-H-Ras V12	1.7089	0.1023	1.0265	0.2637

AQP4 FigS3

Condition	N1 Norm Fluc/Rluc	N1 SD	N2 Norm Fluc/Rluc	N2 SD	N3 Norm Fluc/Rluc	N3 SD
Vector	1	0.1713	1.045	0.1559	1	0.1168
Vector /E7	1.204	0.2172	1.121	0.2537	1.147	0.05639
Vector/ E7Δ21-24	0.8017	0.1072	1.321	0.0621	0.8594	0.01346

Fluc/Rluc

CGA Fig S1C

Condition	N1 Fluc/Rluc	N1 SD	N2 Fluc/Rluc	N2 SD
Vector D5	0.1513	0.01336	0.1522	0.0328
H-Ras V12-D5	0.1915	0.03523	0.1917	0.02881
Vector D12	0.177	0.02829	0.2036	0.05782
H-Ras V12-D12	0.1877	0.05967	0.1653	0.01256
Vector D20	0.1976	0.01948	0.1937	0.03903
H-Ras V12-D20	0.2119	0.02777	0.2063	0.04968

CGA Fig S2A

Condition	N1 Fluc/Rluc	N1 SD	N2 Fluc/Rluc	N2 SD	N3 Fluc/Rluc	N3 SD
Vector	0.1586	0.03595	0.1653	0.0114	0.1709	0.03088
H Ras V12	0.1553	0.007879	0.1812	0.06017	0.1478	0.02945
Vector Gen	0.1961	0.05043	0.2048	0.04365	0.1997	0.03816
H Ras V12 Gen	0.229	0.009903	0.2198	0.006688	0.1437	0.005979

UGA Fig S2B

Condition	N1 Fluc/Rluc	N1 SD	N2 Fluc/Rluc	N2 SD	N3 Fluc/Rluc	N3 SD
Vector	1.000	0.066	1.000	0.154	1.000	0.255
H Ras V12	0.625	0.162	0.748	0.063	0.620	0.034
Vector CDX5-1	0.973	0.099	1.045	0.094		
H Ras V12 CDX5-1	0.837	0.178	0.860	0.020		
Vector Gen	7.038	0.741	5.910	0.425	5.430	1.054
H Ras V12 Gen	3.702	0.460	3.875	0.442	3.067	1.222
Vector CDX5-1 + Gen	16.928	3.800	14.750	1.686	14.111	0.822
H Ras V12 CDX5-1 + Gen	4.896	0.400	6.764	0.990	5.191	0.769

Biology Open • Supplementary information

Table S5. Real time PCR primers

Gene Name	5' forward primer (5'-3')	3' reverse primer (5'-3')
BRCA1	GGTTGTTGATGTGGAGGGGCAA	CCAGATTCCAGGTAAGGGGTTC
CDKN1A/p21	ACCCTTGTGCCTCGCTCAGG	GCGTTTGGAGTGGTAGAAATCTGT
CDK4	CAGTGCAGTCGGTGGTACCTGA	GGCAGAGATTCGCTTGTGTGG
CENPA	AATGGATTCTGCGATGCTGTCTGG	TTTTCAGGCCTTTGGAACGGTGTT
FANCD2	CCTTAGTAGCCGACTGAAACAGG	TGCAAGTAATGGACGCTCTG
GADD45A	TTGCAATATGACTTTGGAGGAA	CATCCCCCACCTTATCCAT
HMBS	GGCAATGCGGCTGCAA	GGGTACCCACGCGAATCAC
H-RAS	TTGCCATCAACAACACCAAGTC	ATCCGAGTCCTTCACCCGTTTG
KI67	AGAAGACAGTACCGCAGATGA	CGGCTCACTAATTTAACGCTGG
MCM6	ATCCCTCTTGCCAAGGATTT	GAAAAGTTCCGCTCACAAGC
ORC1L	CCCGGCTCAAGCATCYAAAGG	TCGTGCATCTCCAGACAGTCT
TBP	GCTGGCCCATAGTGATCTTTGC	CTTCACACGCCAAGAAACAGTGA
MCM6	ATCCCTCTTGCCAAGGATTT	GAAAAGTTCCGCTCACAAGC
IL-6	CCAGGAGCCCAGCTATGAACTC	AAGGCAGCAGGCAACACCAG
CXCL8/IL-8	GGCACAAACTTTCAGAGACAGCA	GGCAAAACTGCACCTTCACACA
